# Supplementary material for: Glucose-derived glutamate drives neuronal terminal differentiation in vitro
Source: EMBO Rep. 2024 Jan 19;25(3):10. doi: 10.1038/s44319-023-00048-8 (PMC10933318; doi:10.1038/s44319-023-00048-8)
Supplement: Supplementary file 1 — Appendix Figures [file 44319_2023_48_MOESM1_ESM.pdf]

Appendix for

Glucose-derived glutamate drives neuronal terminal differentiation in *vitro*

Laura D'Andrea, Matteo Audano, Silvia Pedretti, Silvia Pelucchi, Ramona Stringhi, Gabriele Imperato, Giulia De Cesare, Clara Cambria, Marine H Laporte, Nicola Zamboni, Flavia Antonucci, Monica Di Luca, Nico Mitro, Elena Marcello.

Index

Appendix Figure S1……………………………………………………………….. pag.2
Appendix Figure S2 ………………………………………………………………. pag.3
Appendix Figure S3 ……………………………………………………………..... pag.4
Appendix Figure S4 ………………………………………………………………. pag.5

Appendix Figure S1

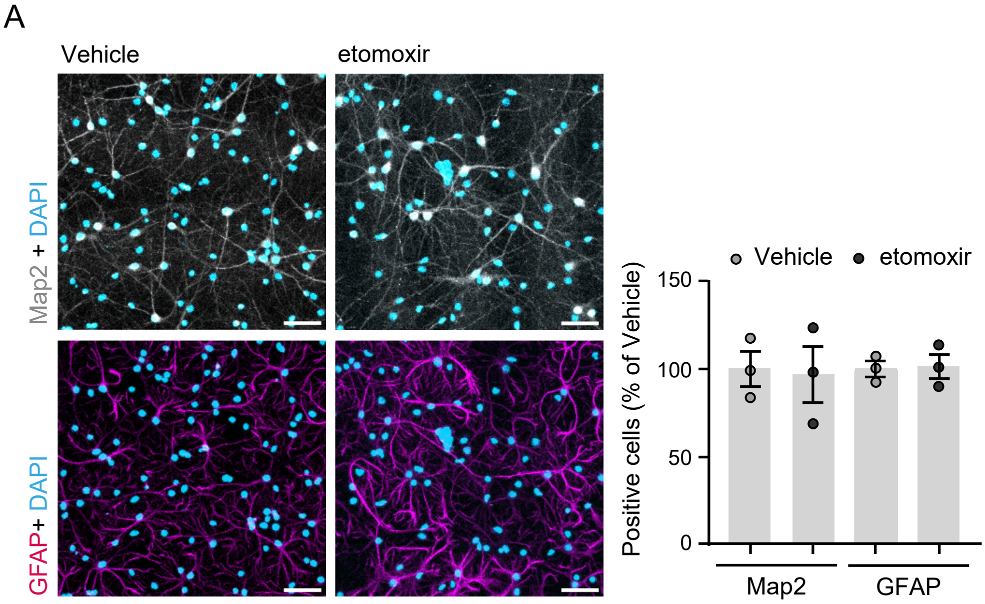

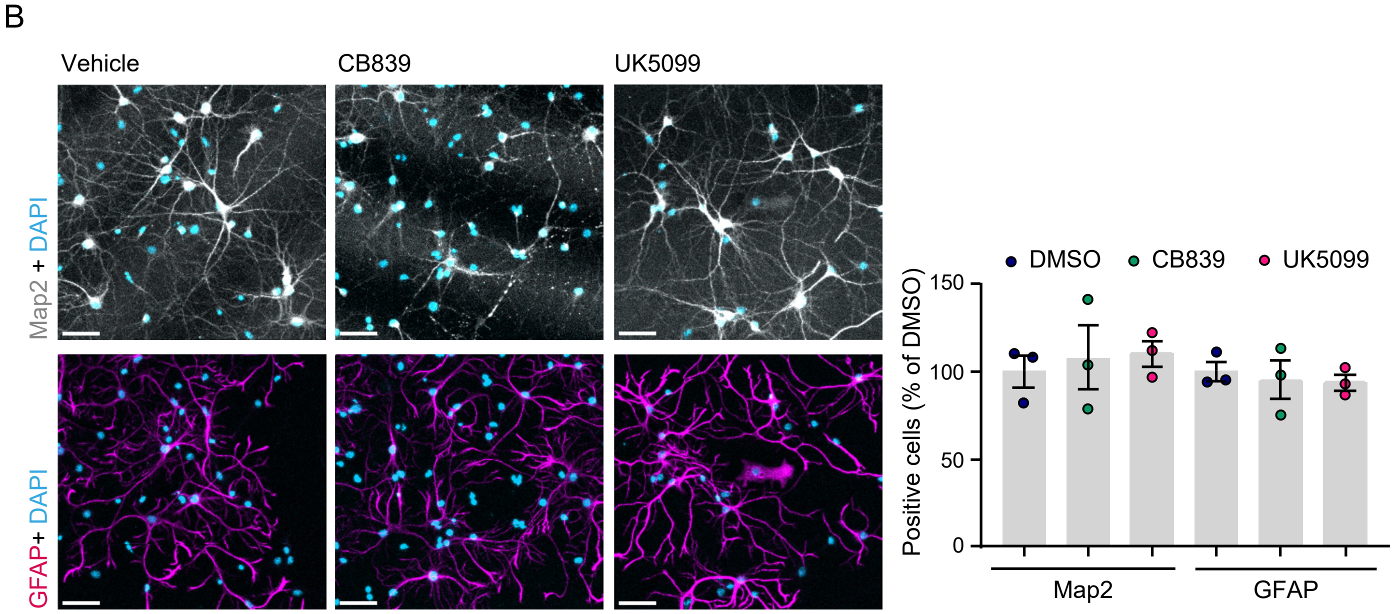

Etomoxir, CB839 and UK5099 treatments do not affect the proportion of Map2 and GFAP positive cells

A-B Representative confocal images of Map2, GFAP and DAPI immunostaining in primary cultures exposed at DIV1 to etomoxir (A) and CB839 or UK5099 (B) compared to controls (Vehicle in A, DMSO in B). The graphs on the right show the quantification of Map2 and GFAP positive cells upon the different treatments administration, expressed as percentage of control. Data are presented as mean +/- SE (n=3 independent experiments; Etomoxir vs Vehicle: paired t-test Map2: t=0.5450, df=2 p=0.6404; GFAP: t=0.5669, df=2, p=0.6279; CB839 vs DMSO: paired t-test: Map2: t=0.2881, df=2 p=0.8004; GFAP: t=0.2880, df=2, p=0.8004; UK5099 vs DMSO: paired t-test: Map2: t=2.412, df=2 p=0.1373; GFAP: t=0.2413, df=2, p=0.1373. Scale Bar 50 µm; Map2: grey, GFAP: magenta, DAPI: cyan).

Appendix Figure S2

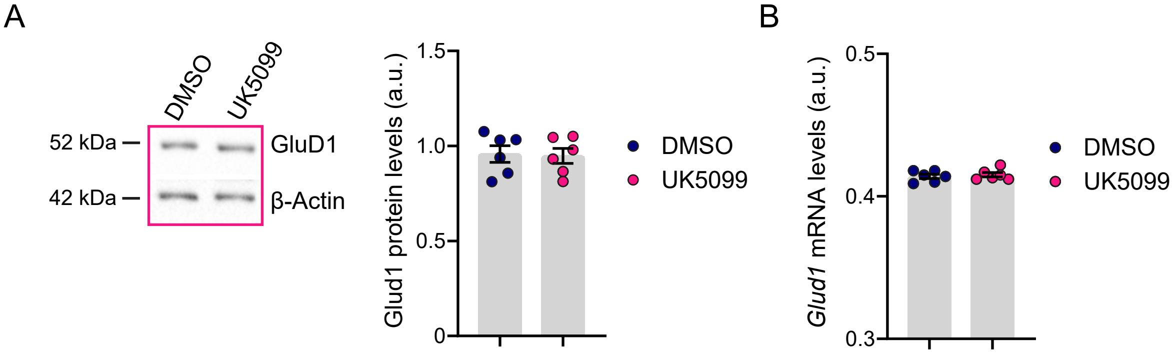

Blocking Mpc1 activity does not affect glutamate dehydrogenase expression

A Representative western blot and quantification of Glud1 protein levels in total extract of DIV15 rat primary hippocampal neurons treated at DIV1 with UK5099 or control (DMSO) (n= 6 independent experiments, mean +/- SE, paired t-test, p=0.8863, t=0.1504, df=5).

B RT-qPCR analysis of *Glud1* mRNA extracted from DIV15 rat primary hippocampal neurons exposed at DIV1 to UK5099 compared to DMSO. *Glud1* levels were normalised on the geometric mean of *Tubulin* and *Histone H3* and expressed as $2^{\wedge -\Delta Ct}$ (n=6 independent experiments, mean +/- SE, paired t-test, p=0.6940, t=0.4170, df=5)

Appendix Figure S3

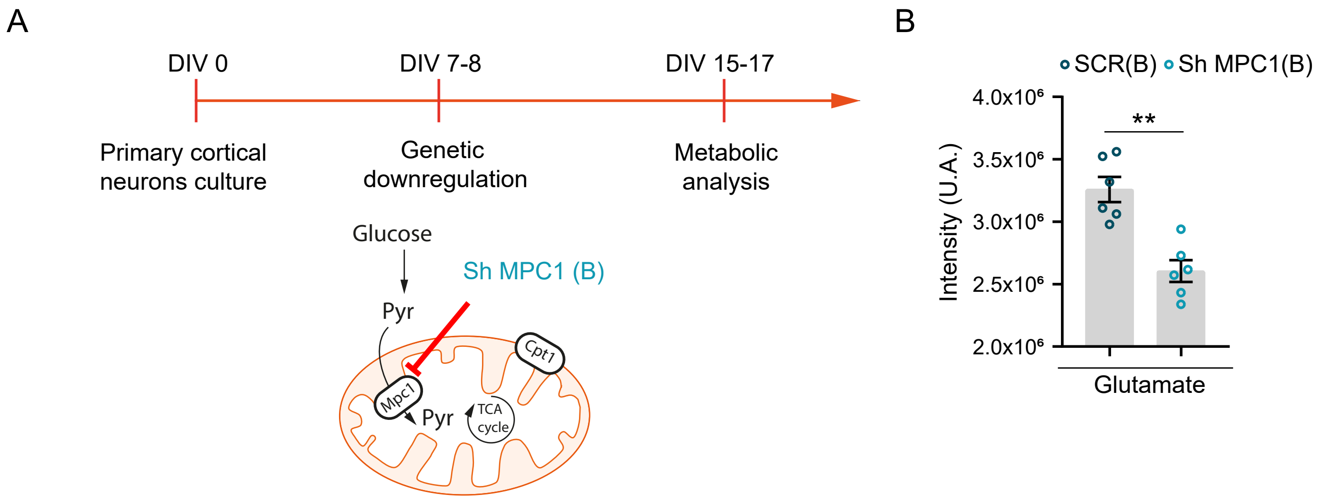

Mpc1 downregulation reduces glutamate levels in cortical neurons

A Time line of the experimental procedure. Primary cortical neurons were isolated and plated (DIV0). Neurons were transduced with lentiviral particles containing shRNA targeting Mpc1 [shMpc1 (B)] or a control sequence [SCR (B)] at DIV7-8. Analysis was performed at DIV15-17.

B The steady-state abundance of glutamate in cultured cortical neurons treated with control or Mpc1 shRNA was determined by untargeted metabolomics (n=6 independent neuronal culture per condition. Mean +/- SE SCR: 3258573 ±100818 A.U.; ShMpc1(B): 2604845 ± 87296 A.U., unpaired t-test p=0.0006).

Appendix Figure S4

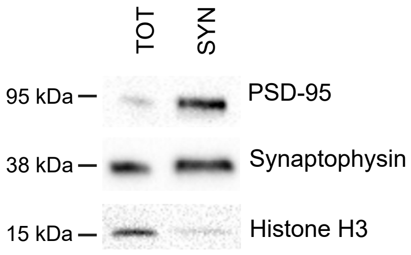

Synaptoneurosomes preparation purity

Representative western blotting for the synaptic markers PSD-95 and synaptophysin and for the nuclear marker histone H3 (Tot: total extract, SYN: synaptoneurosomes)
