# Supplementary material for: Glucose-derived glutamate drives neuronal terminal differentiation in vitro
Source: EMBO Rep. 2024 Jan 19;25(3):10. doi: 10.1038/s44319-023-00048-8 (PMC10933318; doi:10.1038/s44319-023-00048-8)
Supplement: Supplementary file 2 — Table EV1 [file 44319_2023_48_MOESM2_ESM.pdf]

Table EV1. Statistics of Sholl analysis in neurons exposed to UK5099 alone or in presence of αKG.
Two-way ANOVA, Tukey' s multiple comparison test.

Tukey's multiple comparisons test	Mean Diff	95% CI of diff	Significant?	Summary	Adjusted P Value
0 µm					
DMSO vs. UK5099	0	-1.938 to 1.938	No	ns	>0.9999
DMSO vs. UK5099+αKG	0	-1.938 to 1.938	No	ns	>0.9999
UK5099 vs. UK5099+αKG	0	-1.938 to 1.938	No	ns	>0.9999
5 µm					
DMSO vs. UK5099	-0.06429	-2.002 to 1.874	No	ns	0.9966
DMSO vs. UK5099+αKG	0.004464	-1.933 to 1.942	No	ns	>0.9999
UK5099 vs. UK5099+αKG	0.06875	-1.869 to 2.007	No	ns	0.9961
10 µm					
DMSO vs. UK5099	-0.9634	-2.901 to 0.9746	No	ns	0.4709
DMSO vs. UK5099+αKG	-0.07411	-2.012 to 1.864	No	ns	0.9955
UK5099 vs. UK5099+αKG	0.8893	-1.049 to 2.827	No	ns	0.5261
15 µm					
DMSO vs. UK5099	-1.731	-3.669 to 0.2067	No	ns	0.0906
DMSO vs. UK5099+αKG	-0.4625	-2.4 to 1.475	No	ns	0.84
UK5099 vs. UK5099+αKG	1.269	-0.6692 to 3.207	No	ns	0.2724
20 µm					
DMSO vs. UK5099	-1.972	-3.91 to -0.03436	Yes	*	0.045
DMSO vs. UK5099+αKG	-0.6295	-2.567 to 1.308	No	ns	0.7243
UK5099 vs. UK5099+αKG	1.343	-0.5951 to 3.281	No	ns	0.2334
25 µm					
DMSO vs. UK5099	-1.558	-3.496 to 0.3799	No	ns	0.1421
DMSO vs. UK5099+αKG	-0.3473	-2.285 to 1.591	No	ns	0.9063
UK5099 vs. UK5099+αKG	1.211	-0.7272 to 3.149	No	ns	0.3056
30 µm					
DMSO vs. UK5099	-0.4723	-2.41 to 1.466	No	ns	0.8337
DMSO vs. UK5099+αKG	0.3214	-1.617 to 2.259	No	ns	0.9192
UK5099 vs. UK5099+αKG	0.7937	-1.144 to 2.732	No	ns	0.5992
35 µm					
DMSO vs. UK5099	-0.1429	-2.081 to 1.795	No	ns	0.9835
DMSO vs. UK5099+αKG	0.6545	-1.283 to 2.592	No	ns	0.7056
UK5099 vs. UK5099+αKG	0.7973	-1.141 to 2.735	No	ns	0.5964
40 µm					
DMSO vs. UK5099	-0.000893	-1.939 to 1.937	No	ns	>0.9999
DMSO vs. UK5099+αKG	1.546	-0.3915 to 3.484	No	ns	0.1462
UK5099 vs. UK5099+αKG	1.547	-0.3906 to 3.485	No	ns	0.1459
45 µm					
DMSO vs. UK5099	0.4964	-1.442 to 2.434	No	ns	0.8181
DMSO vs. UK5099+αKG	1.685	-0.2531 to 3.623	No	ns	0.1026
UK5099 vs. UK5099+αKG	1.188	-0.7496 to 3.126	No	ns	0.319
50 µm					
DMSO vs. UK5099	0.983	-0.9549 to 2.921	No	ns	0.4566
DMSO vs. UK5099+αKG	2.303	0.3647 to 4.241	Yes	*	0.0151
UK5099 vs. UK5099+αKG	1.32	-0.6183 to 3.258	No	ns	0.2452
55 µm					
DMSO vs. UK5099	0.8786	-1.059 to 2.817	No	ns	0.5342
DMSO vs. UK5099+αKG	2.328	0.3897 to 4.266	Yes	*	0.0138
UK5099 vs. UK5099+αKG	1.449	-0.4889 to 3.387	No	ns	0.1842
60 µm					
DMSO vs. UK5099	1.232	-0.7058 to 3.17	No	ns	0.2931
DMSO vs. UK5099+αKG	2.681	0.7433 to 4.619	Yes	**	0.0036
UK5099 vs. UK5099+αKG	1.449	-0.4889 to 3.387	No	ns	0.1842
65 µm					
DMSO vs. UK5099	1.024	-0.9139 to 2.962	No	ns	0.4273
DMSO vs. UK5099+αKG	2.621	0.6826 to 4.558	Yes	**	0.0046
UK5099 vs. UK5099+αKG	1.596	-0.3415 to 3.534	No	ns	0.1291
70 µm					
DMSO vs. UK5099	1.244	-0.6942 to 3.182	No	ns	0.2864
DMSO vs. UK5099+αKG	2.447	0.5094 to 4.385	Yes	**	0.0089
UK5099 vs. UK5099+αKG	1.204	-0.7344 to 3.142	No	ns	0.3099
75 µm					
DMSO vs. UK5099	1.626	-0.3121 to 3.564	No	ns	0.1197
DMSO vs. UK5099+αKG	2.91	0.9719 to 4.848	Yes	**	0.0014
UK5099 vs. UK5099+αKG	1.284	-0.654 to 3.222	No	ns	0.2641
80 µm					
DMSO vs. UK5099	1.379	-0.5594 to 3.317	No	ns	0.2159
DMSO vs. UK5099+αKG	2.651	0.7129 to 4.589	Yes	**	0.0041
UK5099 vs. UK5099+αKG	1.272	-0.6656 to 3.21	No	ns	0.2704
85 µm					
DMSO vs. UK5099	2.201	0.2629 to 4.139	Yes	*	0.0215
DMSO vs. UK5099+αKG	2.992	1.054 to 4.93	Yes	***	0.001
UK5099 vs. UK5099+αKG	0.7911	-1.147 to 2.729	No	ns	0.6013
90 µm					
DMSO vs. UK5099	2.031	0.09329 to 3.969	Yes	*	0.0375
DMSO vs. UK5099+αKG	3.059	1.121 to 4.997	Yes	***	0.0007
UK5099 vs. UK5099+αKG	1.028	-0.9103 to 2.966	No	ns	0.4248
95 µm					
DMSO vs. UK5099	2.05	0.112 to 3.988	Yes	*	0.0353
DMSO vs. UK5099+αKG	2.827	0.8888 to 4.765	Yes	**	0.002
UK5099 vs. UK5099+αKG	0.7768	-1.161 to 2.715	No	ns	0.6123
100 µm					
DMSO vs. UK5099	2.128	0.1897 to 4.066	Yes	*	0.0275
DMSO vs. UK5099+αKG	2.748	0.8103 to 4.686	Yes	**	0.0027
UK5099 vs. UK5099+αKG	0.6205	-1.317 to 2.558	No	ns	0.7309
