# Supplementary material for: Glucose-derived glutamate drives neuronal terminal differentiation in vitro
Source: EMBO Rep. 2024 Jan 19;25(3):10. doi: 10.1038/s44319-023-00048-8 (PMC10933318; doi:10.1038/s44319-023-00048-8)
Supplement: Supplementary file 3 — Table EV2 [file 44319_2023_48_MOESM3_ESM.pdf]

Table EV2. Statistics of Sholl analysis in neurons exposed to UK5099 alone or in presence of Glutamate.
Two-way ANOVA, Tukey' s multiple comparison test

Tukey's multiple comparisons test	Mean Diff.	95% CI of diff.	Significant?	Summary	Adjusted P Value
0					
DMSO vs. UK5099	0	-1.527 to 1.527	No	ns	>0.9999
DMSO vs. UK5099+Glu	0	-1.527 to 1.527	No	ns	>0.9999
UK5099 vs. UK5099+Glu	0	-1.527 to 1.527	No	ns	>0.9999
5					
DMSO vs. UK5099	-0.02143	-1.549 to 1.506	No	ns	0.9994
DMSO vs. UK5099+Glu	-0.08214	-1.609 to 1.445	No	ns	0.9911
UK5099 vs. UK5099+Glu	-0.06071	-1.588 to 1.466	No	ns	0.9951
10					
DMSO vs. UK5099	-0.5663	-2.093 to 0.9608	No	ns	0.6558
DMSO vs. UK5099+Glu	-0.2021	-1.729 to 1.325	No	ns	0.9475
UK5099 vs. UK5099+Glu	0.3641	-1.163 to 1.891	No	ns	0.8396
15					
DMSO vs. UK5099	-0.4972	-2.024 to 1.03	No	ns	0.7221
DMSO vs. UK5099+Glu	-0.5036	-2.031 to 1.024	No	ns	0.7161
UK5099 vs. UK5099+Glu	-0.006349	-1.533 to 1.521	No	ns	>0.9999
20					
DMSO vs. UK5099	-0.3929	-1.92 to 1.134	No	ns	0.8159
DMSO vs. UK5099+Glu	-0.6786	-2.206 to 0.8485	No	ns	0.5462
UK5099 vs. UK5099+Glu	-0.2857	-1.813 to 1.241	No	ns	0.8979
25					
DMSO vs. UK5099	-0.1298	-1.657 to 1.397	No	ns	0.978
DMSO vs. UK5099+Glu	-0.5493	-2.076 to 0.9778	No	ns	0.6723
UK5099 vs. UK5099+Glu	-0.4195	-1.947 to 1.108	No	ns	0.793
30					
DMSO vs. UK5099	0.6127	-0.9144 to 2.14	No	ns	0.6105
DMSO vs. UK5099+Glu	0.1207	-1.406 to 1.648	No	ns	0.9809
UK5099 vs. UK5099+Glu	-0.492	-2.019 to 1.035	No	ns	0.7271
35					
DMSO vs. UK5099	0.9468	-0.5803 to 2.474	No	ns	0.31
DMSO vs. UK5099+Glu	0.2279	-1.299 to 1.755	No	ns	0.9338
UK5099 vs. UK5099+Glu	-0.719	-2.246 to 0.8081	No	ns	0.5074
40					
DMSO vs. UK5099	0.9143	-0.6128 to 2.441	No	ns	0.3353
DMSO vs. UK5099+Glu	0.7114	-0.8157 to 2.239	No	ns	0.5146
UK5099 vs. UK5099+Glu	-0.2029	-1.73 to 1.324	No	ns	0.9471
45					
DMSO vs. UK5099	1.146	-0.381 to 2.673	No	ns	0.1814
DMSO vs. UK5099+Glu	0.6893	-0.8378 to 2.216	No	ns	0.5359
UK5099 vs. UK5099+Glu	-0.4567	-1.984 to 1.07	No	ns	0.7597
50					
DMSO vs. UK5099	1.399	-0.1279 to 2.926	No	ns	0.08
DMSO vs. UK5099+Glu	1.066	-0.4607 to 2.594	No	ns	0.2273
UK5099 vs. UK5099+Glu	-0.3328	-1.86 to 1.194	No	ns	0.8641
55					
DMSO vs. UK5099	1.615	0.088 to 3.142	Yes	*	0.0354
DMSO vs. UK5099+Glu	0.7307	-0.7964 to 2.258	No	ns	0.4963
UK5099 vs. UK5099+Glu	-0.8844	-2.411 to 0.6427	No	ns	0.3595
60					
DMSO vs. UK5099	2.161	0.6336 to 3.688	Yes	**	0.0029
DMSO vs. UK5099+Glu	1.474	-0.05279 to 3.001	No	ns	0.061
UK5099 vs. UK5099+Glu	-0.6864	-2.214 to 0.8407	No	ns	0.5386
65					
DMSO vs. UK5099	1.862	0.3344 to 3.389	Yes	*	0.0123
DMSO vs. UK5099+Glu	1.369	-0.1585 to 2.896	No	ns	0.089
UK5099 vs. UK5099+Glu	-0.4929	-2.02 to 1.034	No	ns	0.7262
70					
DMSO vs. UK5099	1.944	0.4174 to 3.472	Yes	**	0.0084
DMSO vs. UK5099+Glu	1.144	-0.3828 to 2.671	No	ns	0.1823
UK5099 vs. UK5099+Glu	-0.8002	-2.327 to 0.7269	No	ns	0.4321
75					
DMSO vs. UK5099	2.397	0.8697 to 3.924	Yes	***	0.0008
DMSO vs. UK5099+Glu	1.679	0.1515 to 3.206	Yes	*	0.0273
UK5099 vs. UK5099+Glu	-0.7183	-2.245 to 0.8088	No	ns	0.5081
80					
DMSO vs. UK5099	1.748	0.2213 to 3.275	Yes	*	0.0203
DMSO vs. UK5099+Glu	1.081	-0.4457 to 2.609	No	ns	0.2181
UK5099 vs. UK5099+Glu	-0.667	-2.194 to 0.8601	No	ns	0.5575
85					
DMSO vs. UK5099	2.191	0.6642 to 3.718	Yes	**	0.0025
DMSO vs. UK5099+Glu	1.404	-0.1235 to 2.931	No	ns	0.0788
UK5099 vs. UK5099+Glu	-0.7877	-2.315 to 0.7394	No	ns	0.4434
90					
DMSO vs. UK5099	2.217	0.6896 to 3.744	Yes	**	0.0022
DMSO vs. UK5099+Glu	1.432	-0.09494 to 2.959	No	ns	0.0711
UK5099 vs. UK5099+Glu	-0.7845	-2.312 to 0.7426	No	ns	0.4463
95					
DMSO vs. UK5099	1.728	0.2007 to 3.255	Yes	*	0.0222
DMSO vs. UK5099+Glu	0.8957	-0.6314 to 2.423	No	ns	0.3502
UK5099 vs. UK5099+Glu	-0.8321	-2.359 to 0.695	No	ns	0.4039
100					
DMSO vs. UK5099	1.771	0.244 to 3.298	Yes	*	0.0184
DMSO vs. UK5099+Glu	0.8986	-0.6285 to 2.426	No	ns	0.3479
UK5099 vs. UK5099+Glu	-0.8725	-2.4 to 0.6546	No	ns	0.3693
