# Supplementary material for: Glucose-derived glutamate drives neuronal terminal differentiation in vitro
Source: EMBO Rep. 2024 Jan 19;25(3):10. doi: 10.1038/s44319-023-00048-8 (PMC10933318; doi:10.1038/s44319-023-00048-8)
Supplement: Supplementary file 4 — Table EV3 [file 44319_2023_48_MOESM4_ESM.pdf]

Table EV3. Statistics of Sholl analysis in neurons exposed to UK5099 alone or in presence of DHPG.
Two-way ANOVA, Tukey' s multiple comparison test.

Tukey's multiple comparisons test	Mean Diff,	95,00% CI of diff,	Significant?	Summary	Adjusted P Value
0					
DMSO vs. UK5099	-0,0625	-2,665 to 2,540	No	ns	0,9982
DMSO vs. UK5099 + DHPG	2,67E-15	-2,602 to 2,602	No	ns	>0,9999
UK5099 vs. UK5099 + DHPG	0,0625	-2,540 to 2,665	No	ns	0,9982
5					
DMSO vs. UK5099	-0,2223	-2,824 to 2,380	No	ns	0,9778
DMSO vs. UK5099 + DHPG	-0,5571	-3,159 to 2,045	No	ns	0,8688
UK5099 vs. UK5099 + DHPG	-0,3348	-2,937 to 2,267	No	ns	0,9504
10					
DMSO vs. UK5099	-0,7036	-3,306 to 1,899	No	ns	0,7992
DMSO vs. UK5099 + DHPG	-0,6812	-3,283 to 1,921	No	ns	0,8104
UK5099 vs. UK5099 + DHPG	0,02232	-2,580 to 2,624	No	ns	0,9998
15					
DMSO vs. UK5099	0,3577	-2,244 to 2,960	No	ns	0,9436
DMSO vs. UK5099 + DHPG	0,2068	-2,395 to 2,809	No	ns	0,9808
UK5099 vs. UK5099 + DHPG	-0,1509	-2,753 to 2,451	No	ns	0,9897
20					
DMSO vs. UK5099	0,875	-1,727 to 3,477	No	ns	0,7072
DMSO vs. UK5099 + DHPG	-0,7643	-3,366 to 1,838	No	ns	0,7676
UK5099 vs. UK5099 + DHPG	-1,639	-4,241 to 0,9628	No	ns	0,2991
25					
DMSO vs. UK5099	1,297	-1,305 to 3,899	No	ns	0,4683
DMSO vs. UK5099 + DHPG	-0,3777	-2,980 to 2,224	No	ns	0,9374
UK5099 vs. UK5099 + DHPG	-1,675	-4,277 to 0,9271	No	ns	0,2838
30					
DMSO vs. UK5099	1,284	-1,318 to 3,886	No	ns	0,4757
DMSO vs. UK5099 + DHPG	-0,8878	-3,490 to 1,714	No	ns	0,7001
UK5099 vs. UK5099 + DHPG	-2,171	-4,774 to 0,4307	No	ns	0,1223
35					
DMSO vs. UK5099	1,887	-0,7155 to 4,489	No	ns	0,2033
DMSO vs. UK5099 + DHPG	-0,7196	-3,322 to 1,882	No	ns	0,7909
UK5099 vs. UK5099 + DHPG	-2,606	-5,208 to -0,004145	Yes	*	0,0495
40					
DMSO vs. UK5099	2,196	-0,4063 to 4,798	No	ns	0,1167
DMSO vs. UK5099 + DHPG	-0,9122	-3,514 to 1,690	No	ns	0,6864
UK5099 vs. UK5099 + DHPG	-3,108	-5,710 to -0,5059	Yes	*	0,0145
45					
DMSO vs. UK5099	2,883	0,2809 to 5,485	Yes	*	0,0258
DMSO vs. UK5099 + DHPG	-0,7562	-3,358 to 1,846	No	ns	0,7719
UK5099 vs. UK5099 + DHPG	-3,639	-6,241 to -1,037	Yes	**	0,0032
50					
DMSO vs. UK5099	2,805	0,2027 to 5,407	Yes	*	0,0312
DMSO vs. UK5099 + DHPG	-1,009	-3,611 to 1,593	No	ns	0,6314
UK5099 vs. UK5099 + DHPG	-3,813	-6,415 to -1,211	Yes	**	0,0019
55					
DMSO vs. UK5099	2,43	-0,1723 to 5,032	No	ns	0,0728
DMSO vs. UK5099 + DHPG	-1,054	-3,656 to 1,548	No	ns	0,6053
UK5099 vs. UK5099 + DHPG	-3,484	-6,086 to -0,8818	Yes	**	0,0051
60					
DMSO vs. UK5099	3,044	0,4419 to 5,646	Yes	*	0,0172
DMSO vs. UK5099 + DHPG	-1,21	-3,813 to 1,392	No	ns	0,5163
UK5099 vs. UK5099 + DHPG	-4,254	-6,857 to -1,652	Yes	***	0,0004
65					
DMSO vs. UK5099	3,078	0,4756 to 5,680	Yes	*	0,0157
DMSO vs. UK5099 + DHPG	-0,4214	-3,024 to 2,181	No	ns	0,9226
UK5099 vs. UK5099 + DHPG	-3,499	-6,101 to -0,8970	Yes	**	0,0049
70					
DMSO vs. UK5099	3,187	0,5851 to 5,789	Yes	*	0,0118
DMSO vs. UK5099 + DHPG	0,04345	-2,559 to 2,646	No	ns	0,9991
UK5099 vs. UK5099 + DHPG	-3,144	-5,746 to -0,5416	Yes	*	0,0132
75					
DMSO vs. UK5099	2,879	0,2768 to 5,481	Yes	*	0,0261
DMSO vs. UK5099 + DHPG	0,08869	-2,513 to 2,691	No	ns	0,9964
UK5099 vs. UK5099 + DHPG	-2,79	-5,392 to -0,1881	Yes	*	0,0323
80					
DMSO vs. UK5099	3,36	0,7580 to 5,962	Yes	**	0,0073
DMSO vs. UK5099 + DHPG	1,575	-1,027 to 4,177	No	ns	0,3278
UK5099 vs. UK5099 + DHPG	-1,785	-4,387 to 0,8173	No	ns	0,2398
85					
DMSO vs. UK5099	2,863	0,2610 to 5,465	Yes	*	0,0271
DMSO vs. UK5099 + DHPG	1,686	-0,9158 to 4,288	No	ns	0,2791
UK5099 vs. UK5099 + DHPG	-1,177	-3,779 to 1,425	No	ns	0,5353
90					
DMSO vs. UK5099	2,393	-0,2089 to 4,995	No	ns	0,0786
DMSO vs. UK5099 + DHPG	1,989	-0,6134 to 4,591	No	ns	0,1707
UK5099 vs. UK5099 + DHPG	-0,4045	-3,007 to 2,198	No	ns	0,9285
95					
DMSO vs. UK5099	1,838	-0,7637 to 4,440	No	ns	0,2201
DMSO vs. UK5099 + DHPG	1,619	-0,9834 to 4,221	No	ns	0,3082
UK5099 vs. UK5099 + DHPG	-0,2196	-2,822 to 2,382	No	ns	0,9784
100					
DMSO vs. UK5099	1,699	-0,9027 to 4,302	No	ns	0,2736
DMSO vs. UK5099 + DHPG	1,703	-0,8991 to 4,305	No	ns	0,2722
UK5099 vs. UK5099 + DHPG	0,003571	-2,599 to 2,606	No	ns	>0,9999
105					
DMSO vs. UK5099	0,825	-1,777 to 3,427	No	ns	0,7349
DMSO vs. UK5099 + DHPG	1,032	-1,571 to 3,634	No	ns	0,6183
UK5099 vs. UK5099 + DHPG	0,2065	-2,396 to 2,809	No	ns	0,9808
110					
DMSO vs. UK5099	0,3083	-2,294 to 2,910	No	ns	0,9578
DMSO vs. UK5099 + DHPG	1,339	-1,263 to 3,941	No	ns	0,4459
UK5099 vs. UK5099 + DHPG	1,03	-1,572 to 3,632	No	ns	0,6189
