# Supplementary figures and images for: Glucose-derived glutamate drives neuronal terminal differentiation in vitro
Source: EMBO Rep. 2024 Jan 19;25(3):10. doi: 10.1038/s44319-023-00048-8 (PMC10933318; doi:10.1038/s44319-023-00048-8)

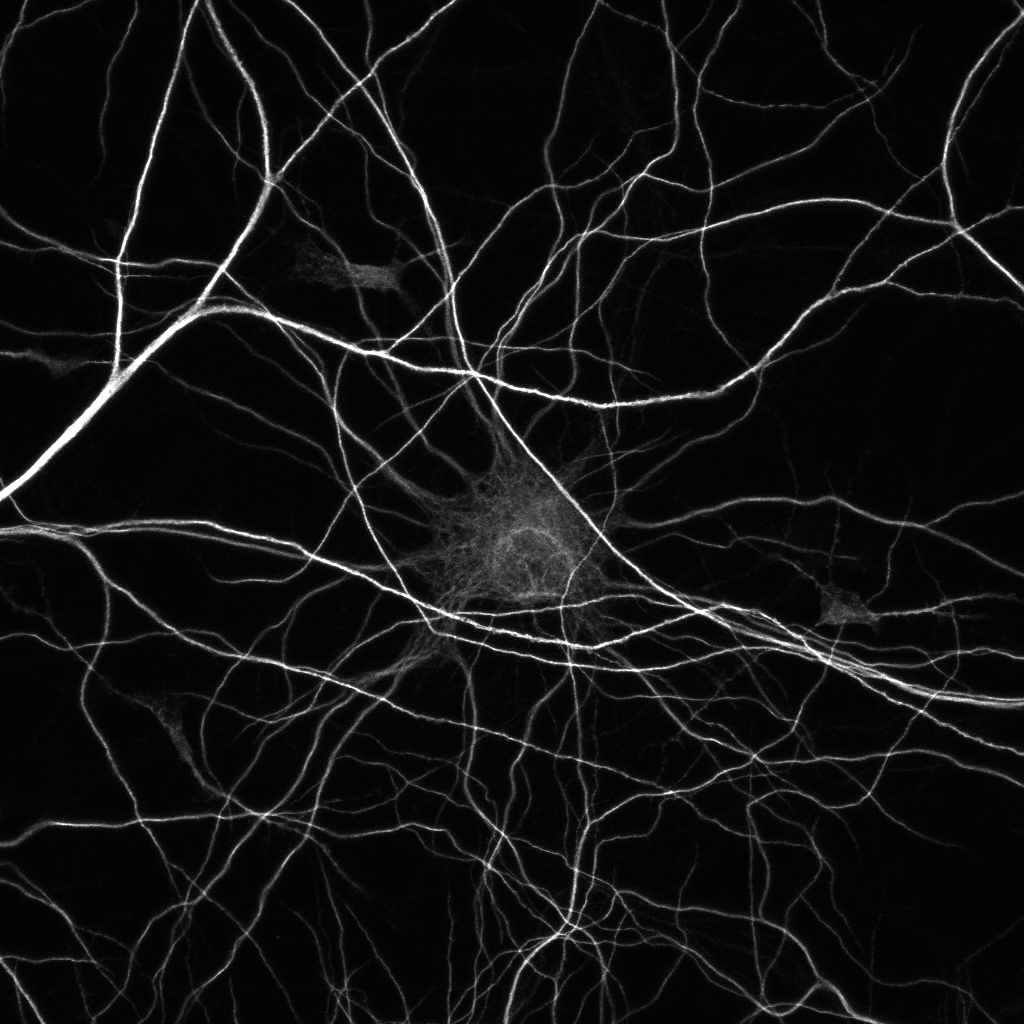

Supplement: Supplementary file 6 — Source Data Fig. 2 [file 44319_2023_48_MOESM6_ESM.zip › Figure 2/Figure 2A/Map2 Etomoxir.tif]

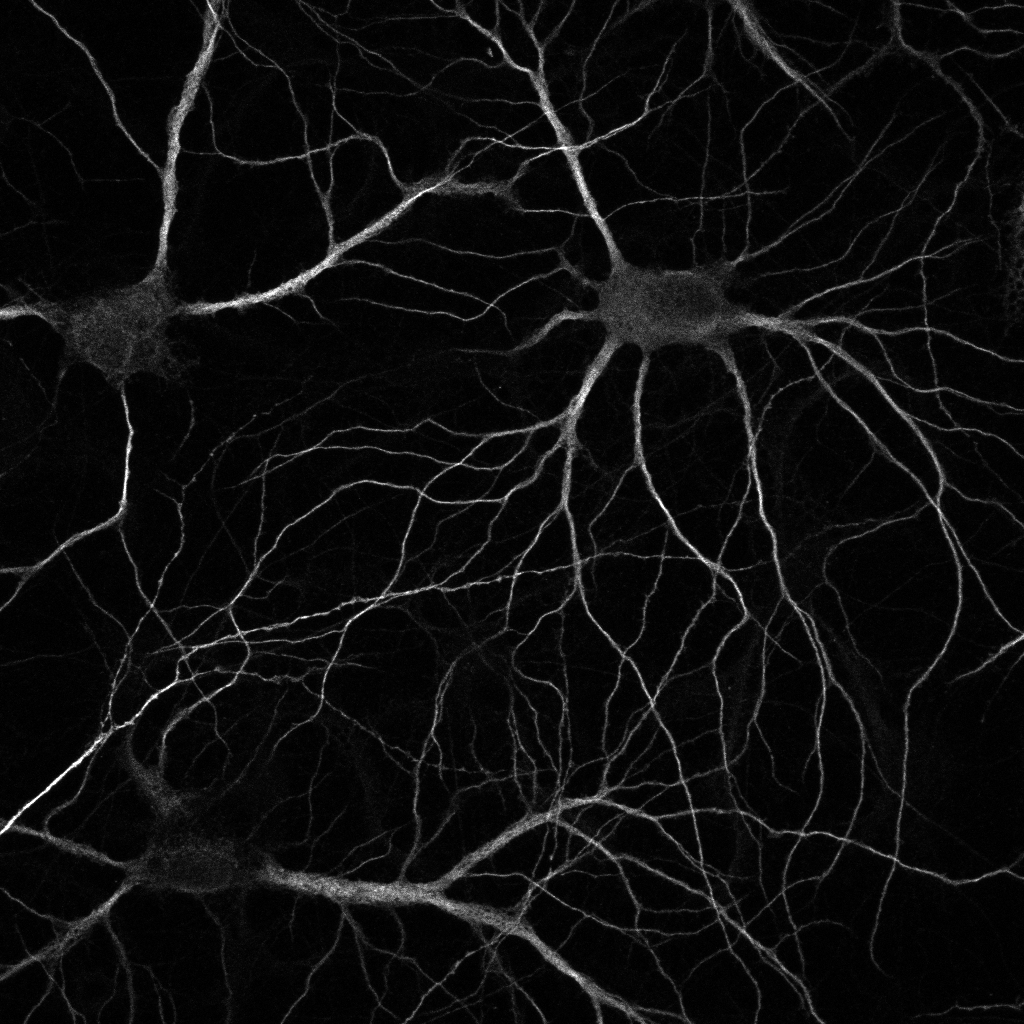

Supplement: Supplementary file 6 — Source Data Fig. 2 [file 44319_2023_48_MOESM6_ESM.zip › Figure 2/Figure 2A/Map2 DMSO.tif]

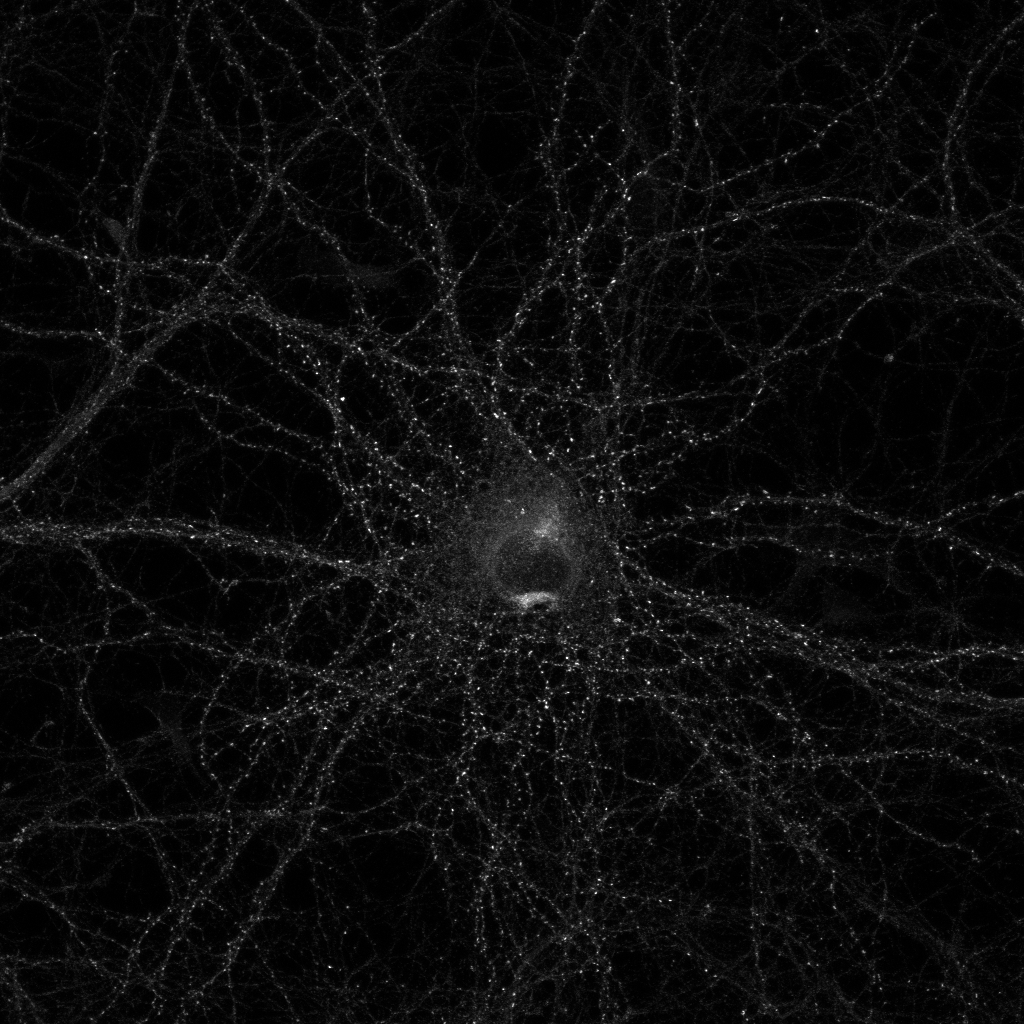

Supplement: Supplementary file 6 — Source Data Fig. 2 [file 44319_2023_48_MOESM6_ESM.zip › Figure 2/Figure 2A/Psd95 etomoxir.tif]

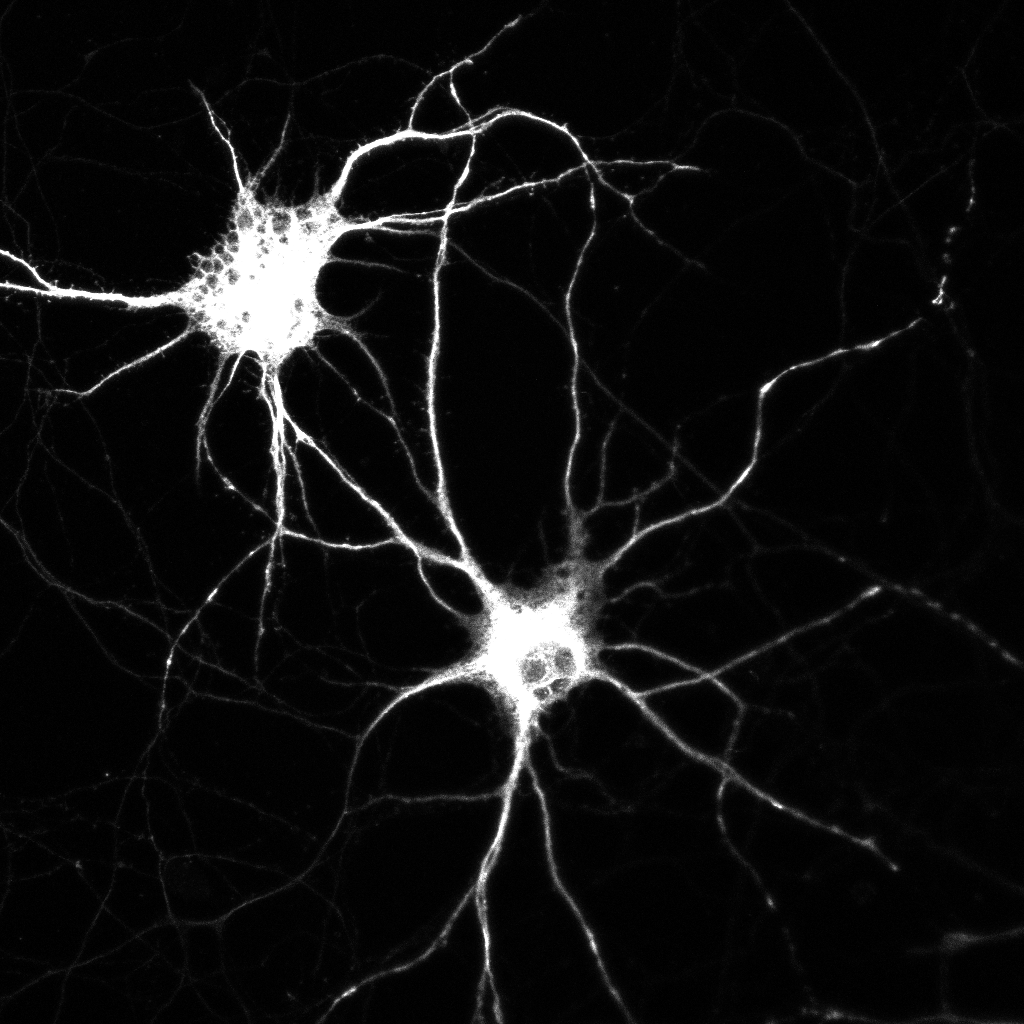

Supplement: Supplementary file 6 — Source Data Fig. 2 [file 44319_2023_48_MOESM6_ESM.zip › Figure 2/Figure 2A/Map2 UK5099.tif]

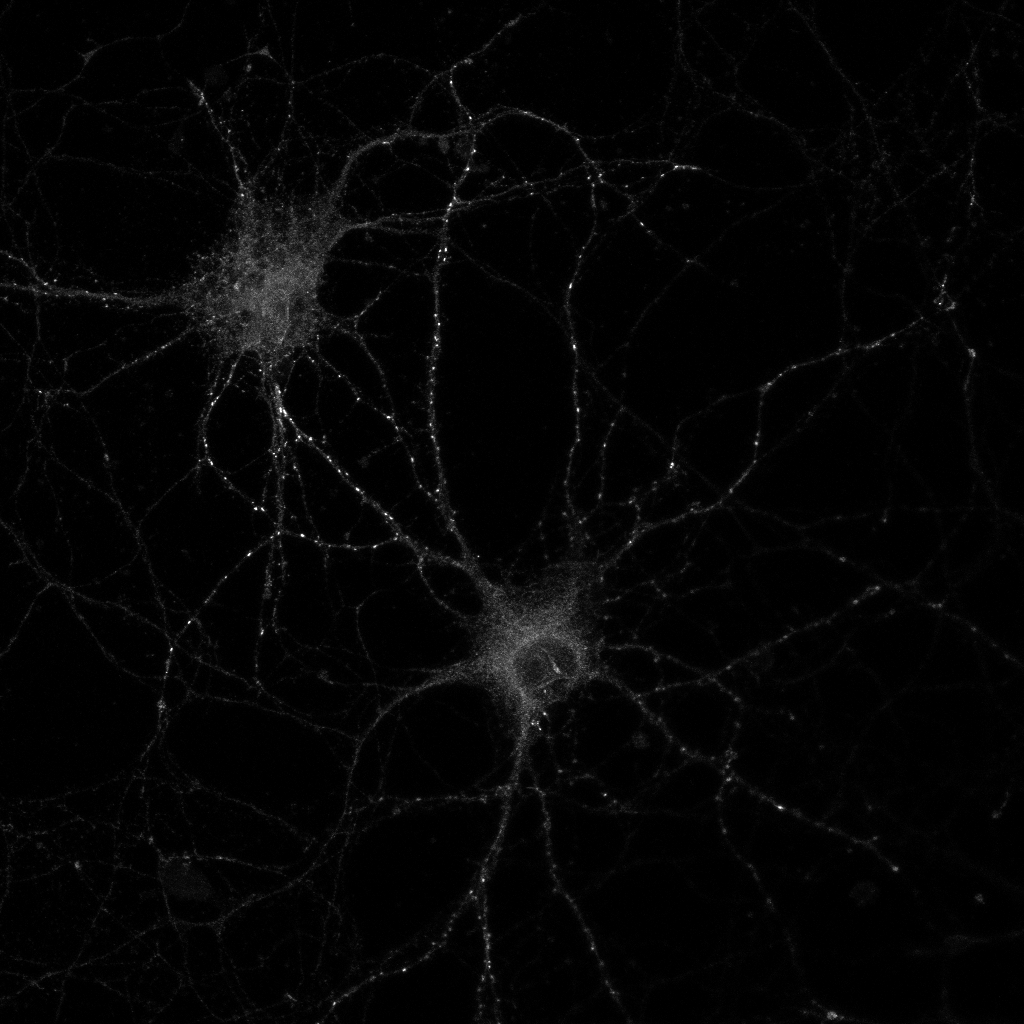

Supplement: Supplementary file 6 — Source Data Fig. 2 [file 44319_2023_48_MOESM6_ESM.zip › Figure 2/Figure 2A/PSD95 UK5099.tif]

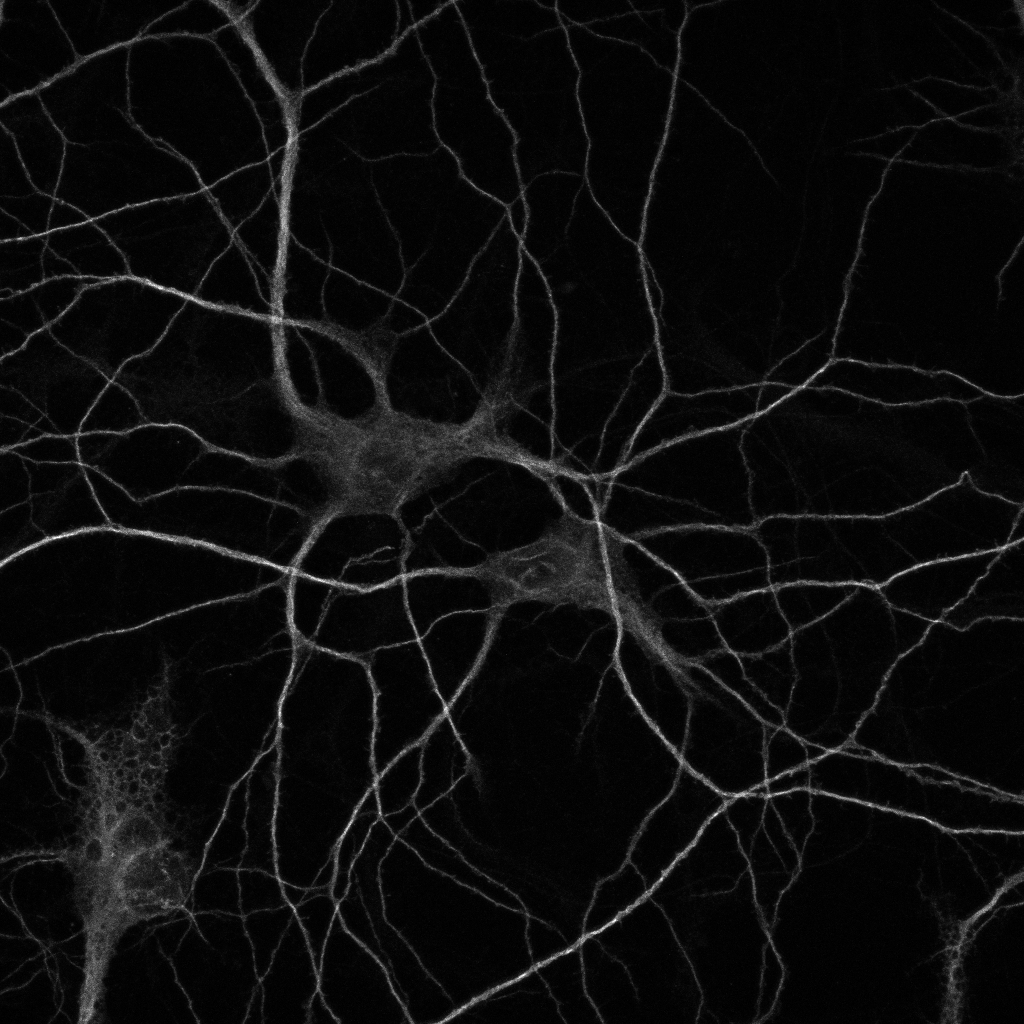

Supplement: Supplementary file 6 — Source Data Fig. 2 [file 44319_2023_48_MOESM6_ESM.zip › Figure 2/Figure 2A/Map2 CB839.tif]

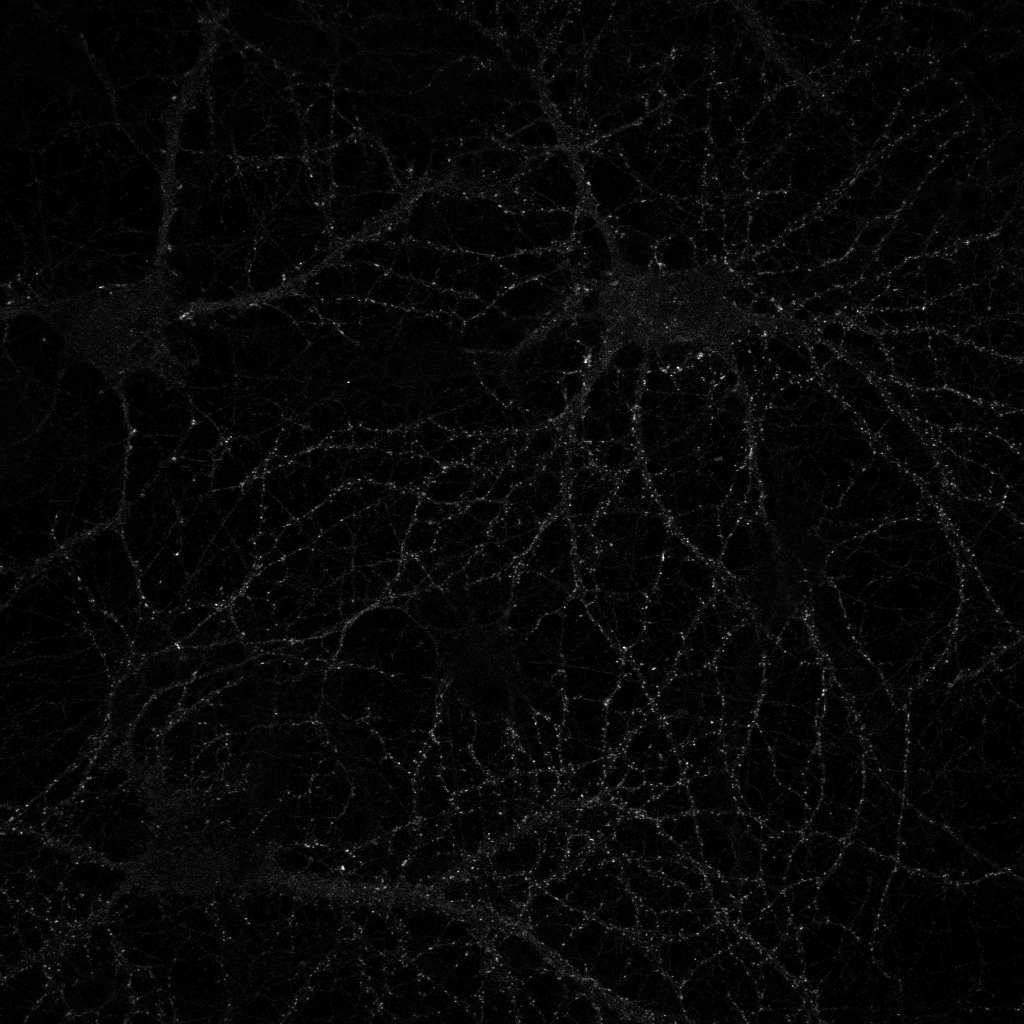

Supplement: Supplementary file 6 — Source Data Fig. 2 [file 44319_2023_48_MOESM6_ESM.zip › Figure 2/Figure 2A/PSD95 DMSO.tif]

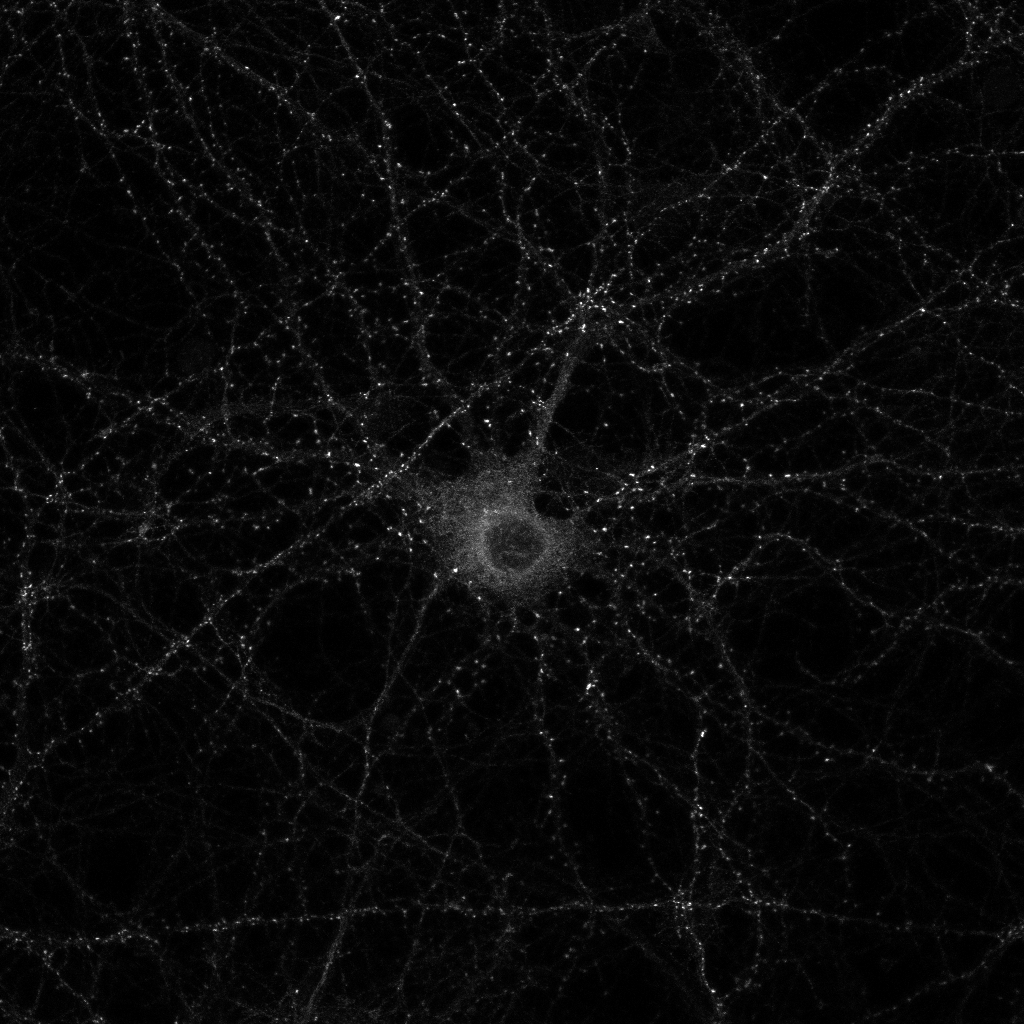

Supplement: Supplementary file 6 — Source Data Fig. 2 [file 44319_2023_48_MOESM6_ESM.zip › Figure 2/Figure 2A/PSD95 Vehicle.tif]

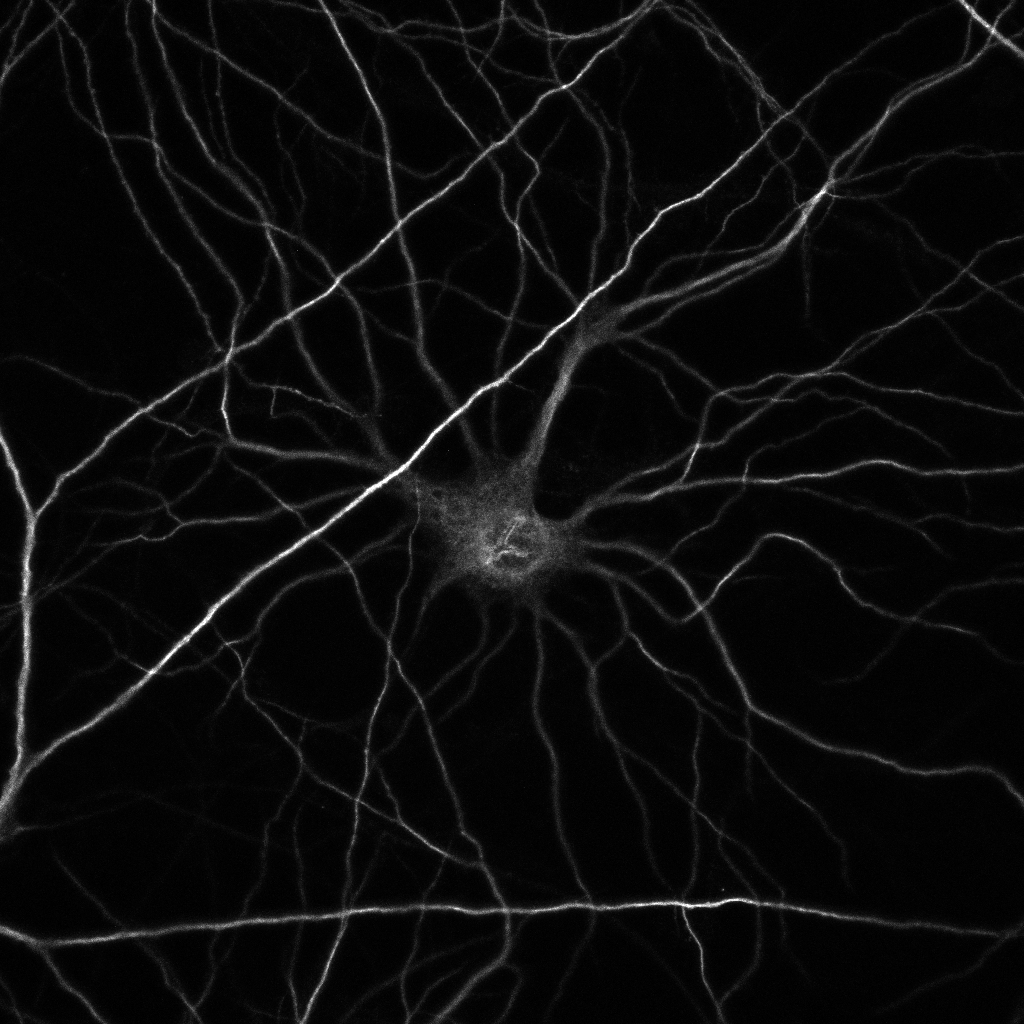

Supplement: Supplementary file 6 — Source Data Fig. 2 [file 44319_2023_48_MOESM6_ESM.zip › Figure 2/Figure 2A/Map2 Vehicle.tif]

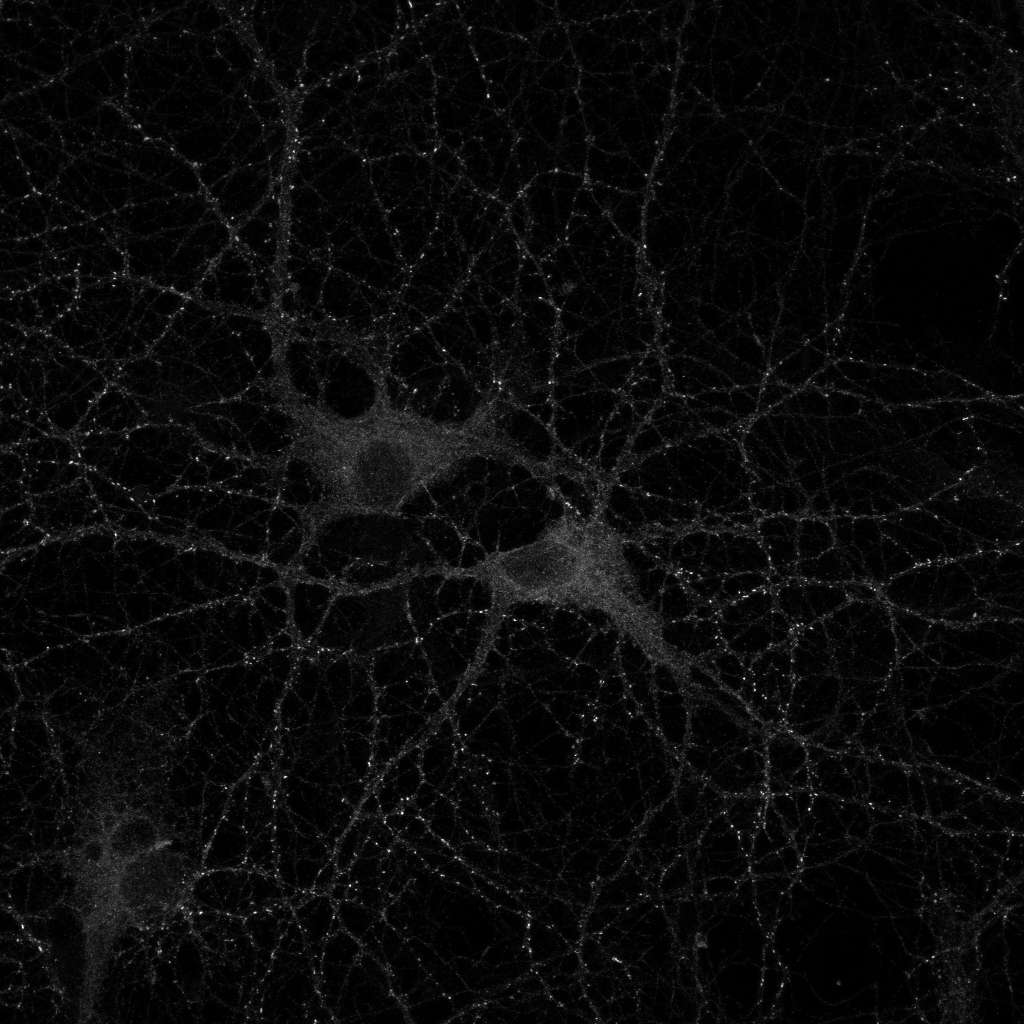

Supplement: Supplementary file 6 — Source Data Fig. 2 [file 44319_2023_48_MOESM6_ESM.zip › Figure 2/Figure 2A/PSD95 CB839.tif]

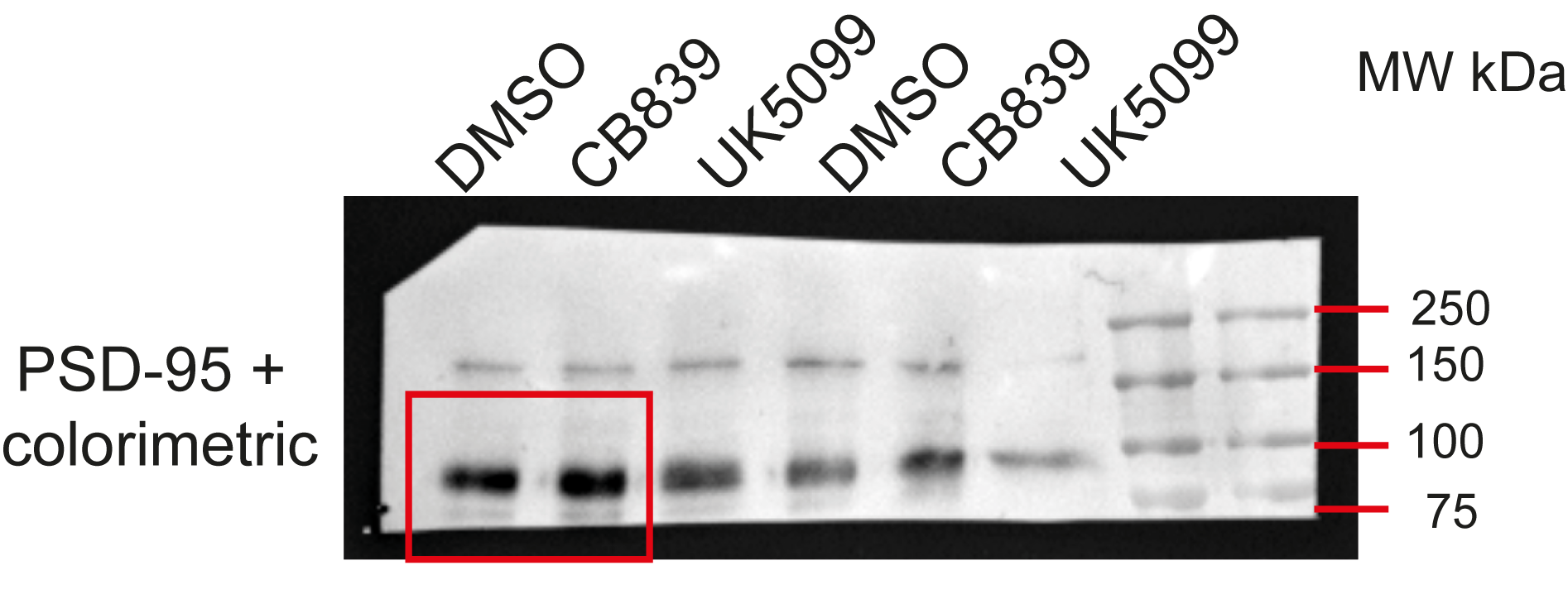

Supplement: Supplementary file 6 — Source Data Fig. 2 [file 44319_2023_48_MOESM6_ESM.zip › Figure 2/Figure 2C/Western PSD95 CB839.tif]

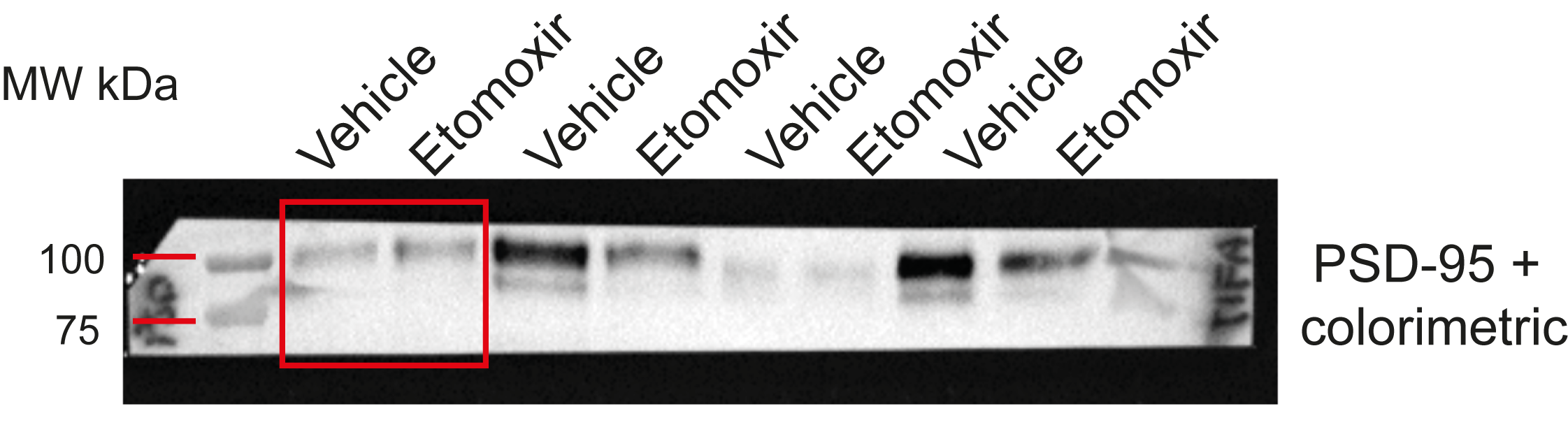

Supplement: Supplementary file 6 — Source Data Fig. 2 [file 44319_2023_48_MOESM6_ESM.zip › Figure 2/Figure 2C/Western PSD95 Etomoxir.tif]

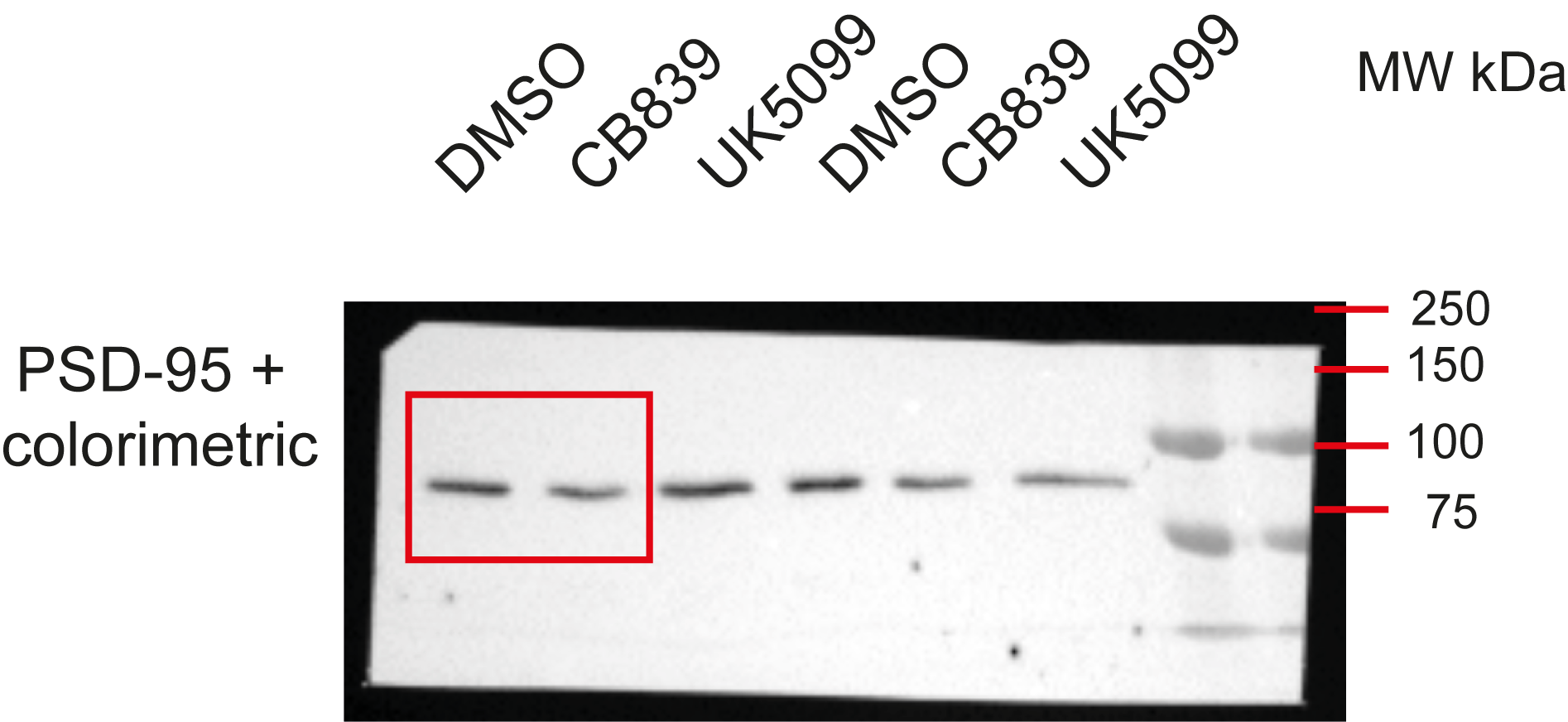

Supplement: Supplementary file 6 — Source Data Fig. 2 [file 44319_2023_48_MOESM6_ESM.zip › Figure 2/Figure 2C/Western Actin CB839.tif]

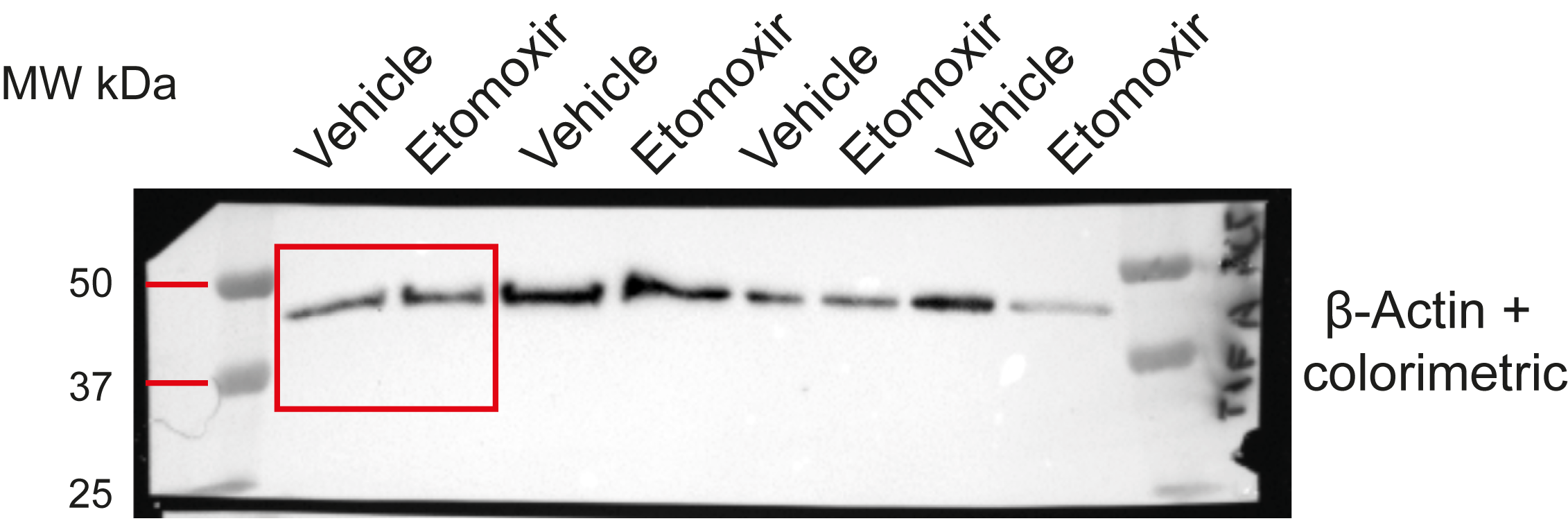

Supplement: Supplementary file 6 — Source Data Fig. 2 [file 44319_2023_48_MOESM6_ESM.zip › Figure 2/Figure 2C/Western Actin Etomoxir.tif]

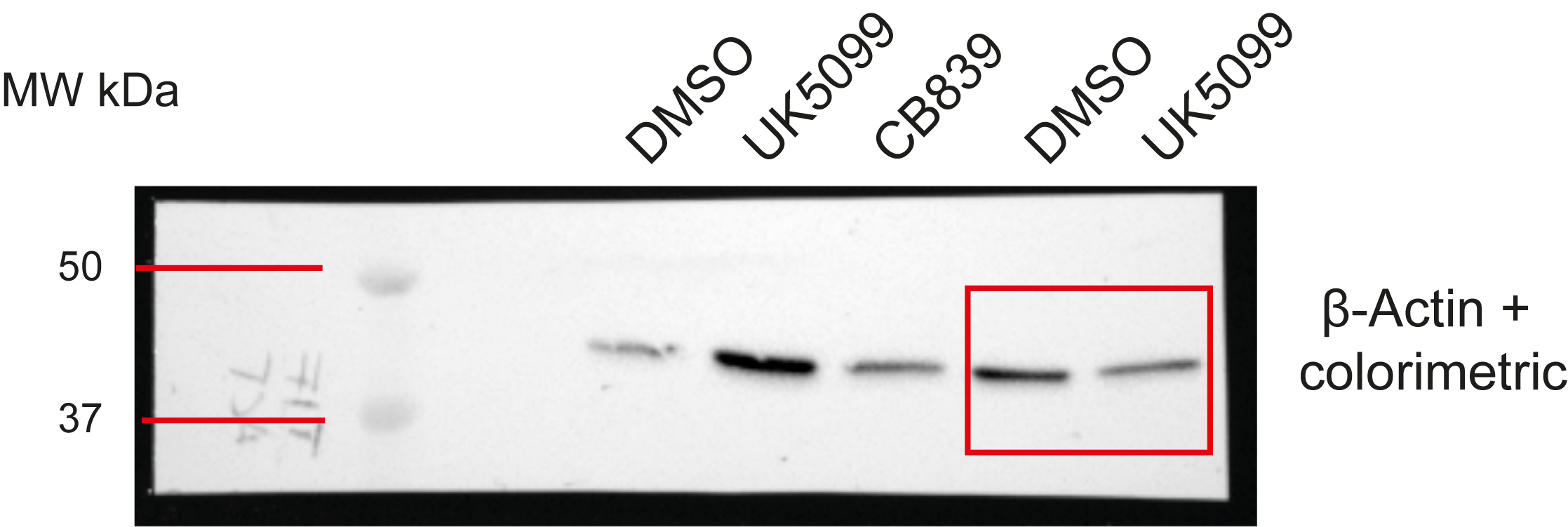

Supplement: Supplementary file 6 — Source Data Fig. 2 [file 44319_2023_48_MOESM6_ESM.zip › Figure 2/Figure 2C/Western Actin UK5099.tif]

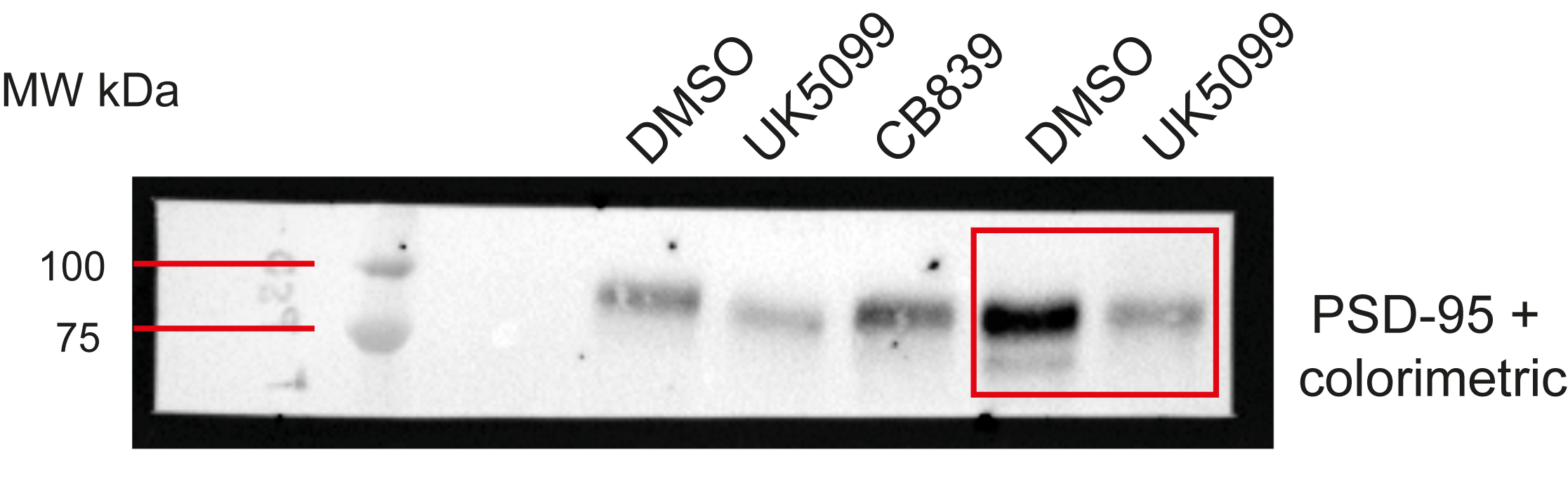

Supplement: Supplementary file 6 — Source Data Fig. 2 [file 44319_2023_48_MOESM6_ESM.zip › Figure 2/Figure 2C/Western PSD95 UK5099.tif]

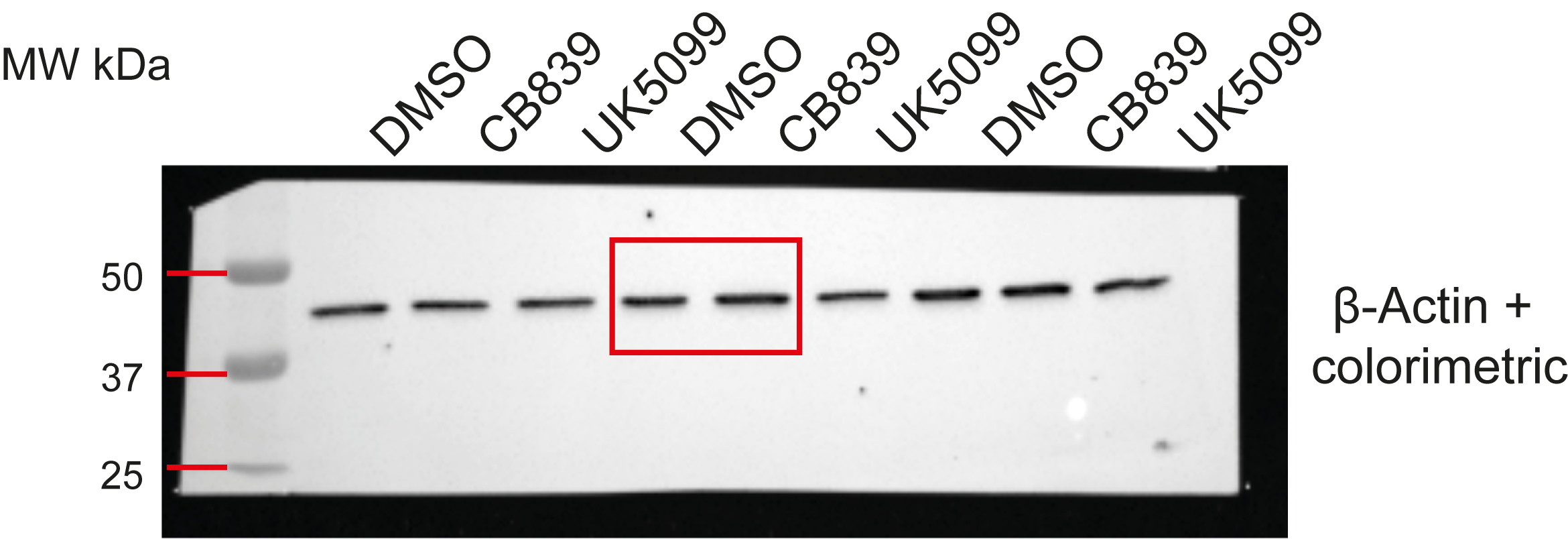

Supplement: Supplementary file 6 — Source Data Fig. 2 [file 44319_2023_48_MOESM6_ESM.zip › Figure 2/Figure 2B/Western blot Actin CB839.tif]

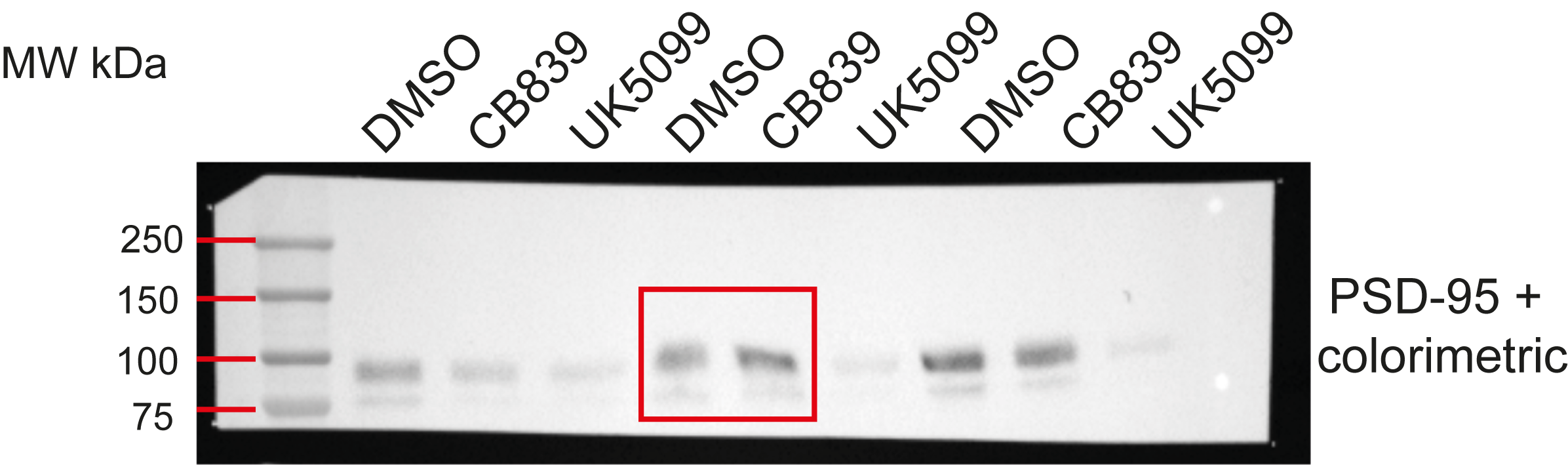

Supplement: Supplementary file 6 — Source Data Fig. 2 [file 44319_2023_48_MOESM6_ESM.zip › Figure 2/Figure 2B/Western PSD95 CB839.tif]

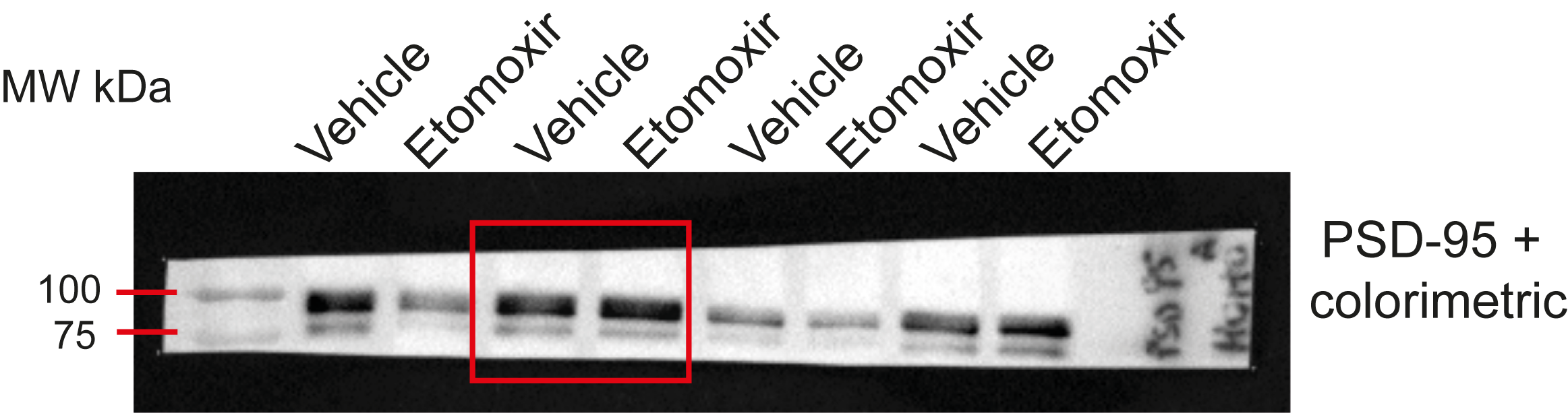

Supplement: Supplementary file 6 — Source Data Fig. 2 [file 44319_2023_48_MOESM6_ESM.zip › Figure 2/Figure 2B/Western PSD95 Etomoxir.tif]

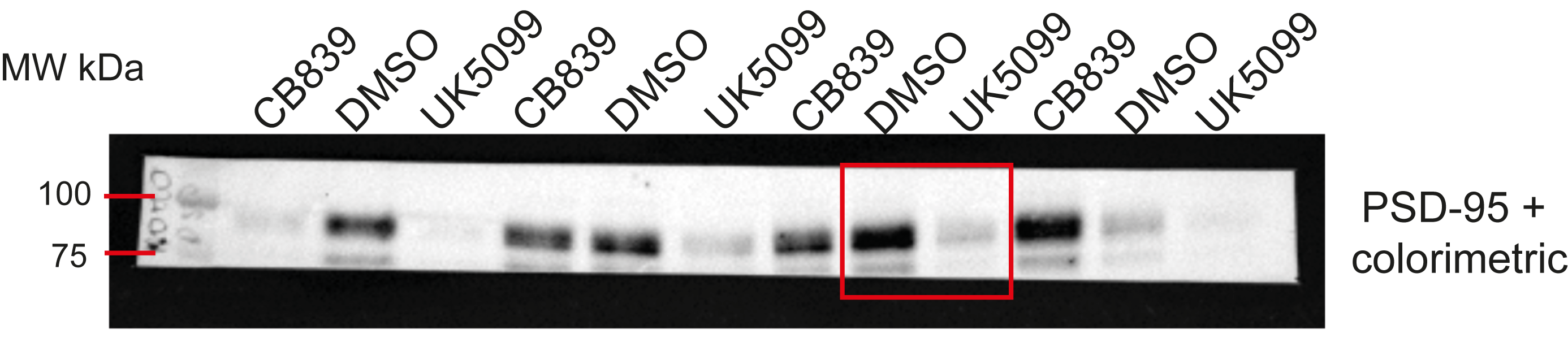

Supplement: Supplementary file 6 — Source Data Fig. 2 [file 44319_2023_48_MOESM6_ESM.zip › Figure 2/Figure 2B/Western blot PSD95 UK5099.tif]

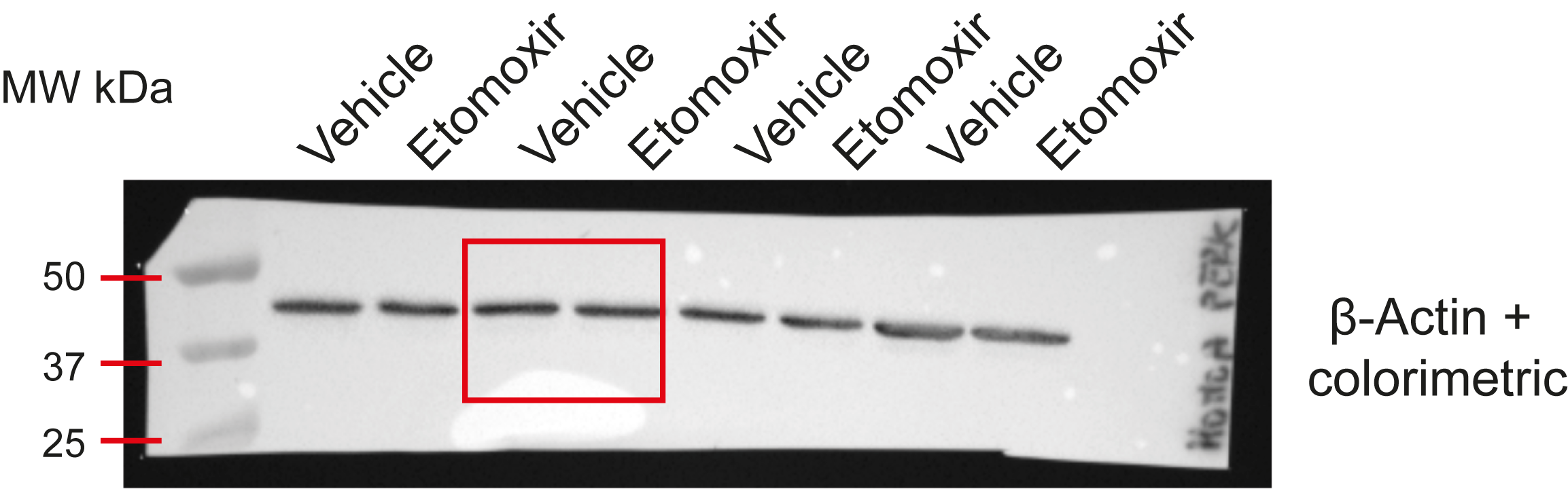

Supplement: Supplementary file 6 — Source Data Fig. 2 [file 44319_2023_48_MOESM6_ESM.zip › Figure 2/Figure 2B/Western Actin Etomoxir.tif]

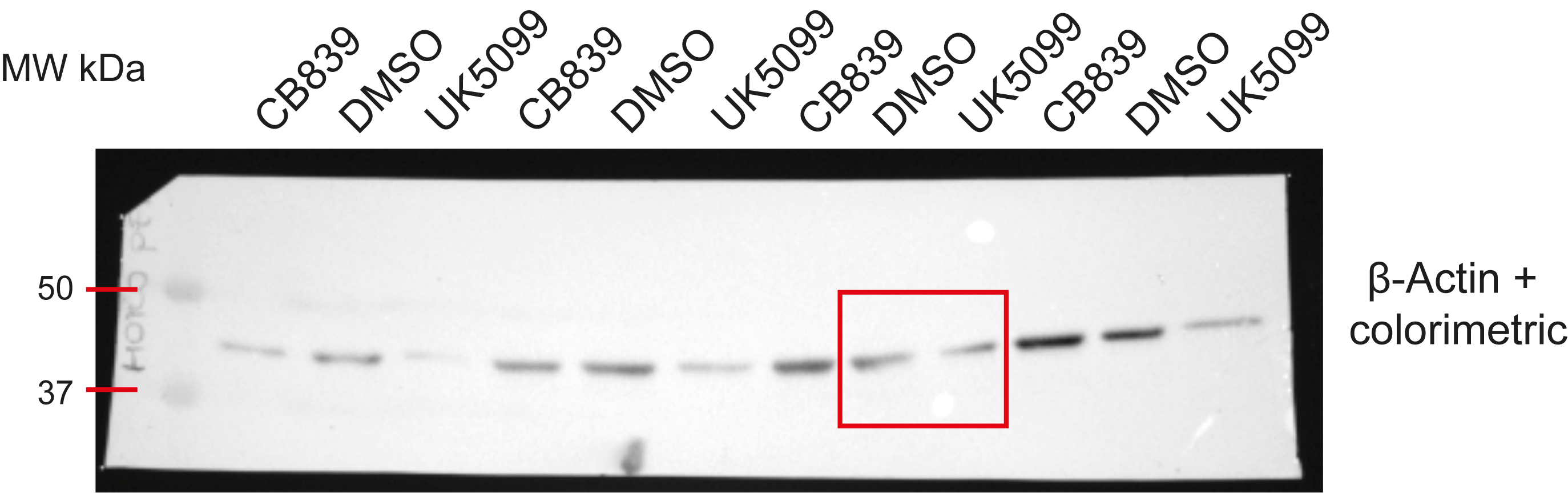

Supplement: Supplementary file 6 — Source Data Fig. 2 [file 44319_2023_48_MOESM6_ESM.zip › Figure 2/Figure 2B/Western blot Actin UK5099.tif]

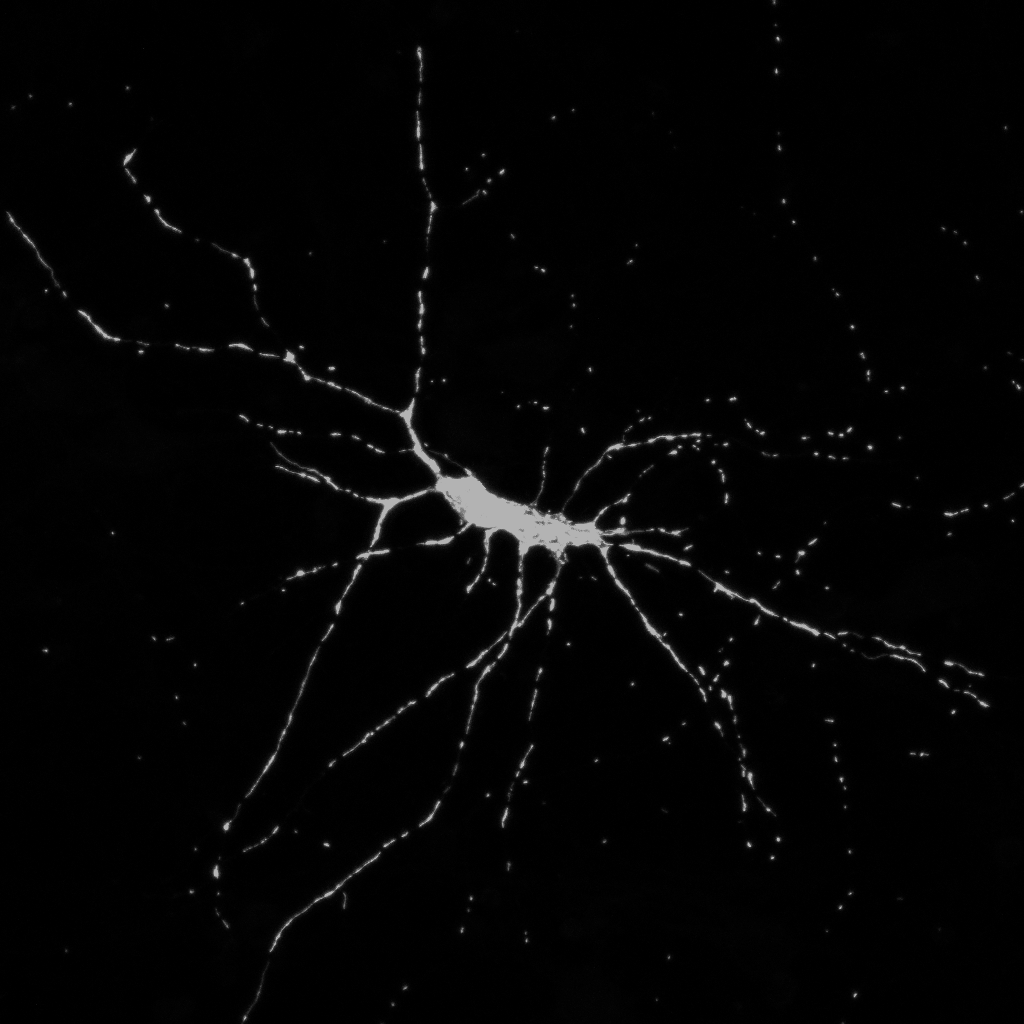

Supplement: Supplementary file 7 — Source Data Fig. 3 [file 44319_2023_48_MOESM7_ESM.zip › Figure 3/Figure 3E/DMSO sv9.tif]

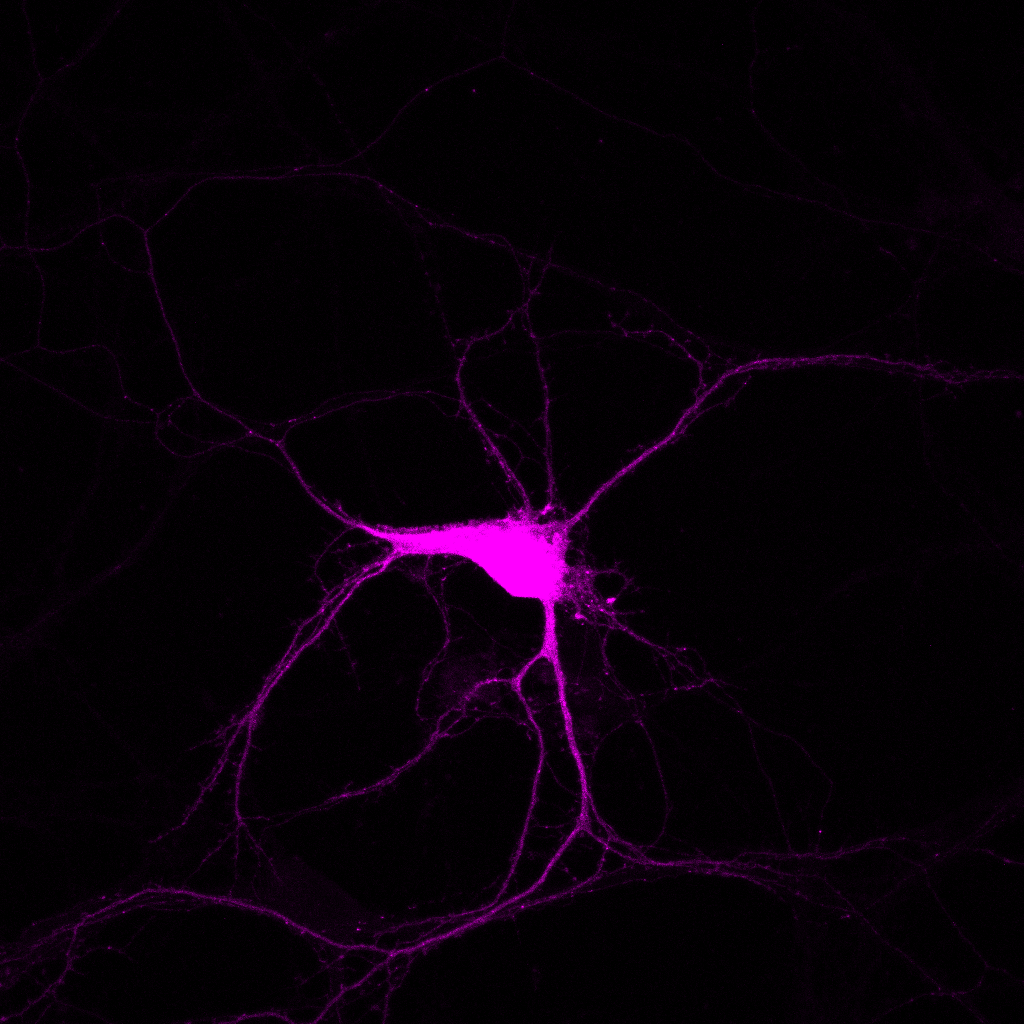

Supplement: Supplementary file 7 — Source Data Fig. 3 [file 44319_2023_48_MOESM7_ESM.zip › Figure 3/Figure 3E/UK mcherry.tif]

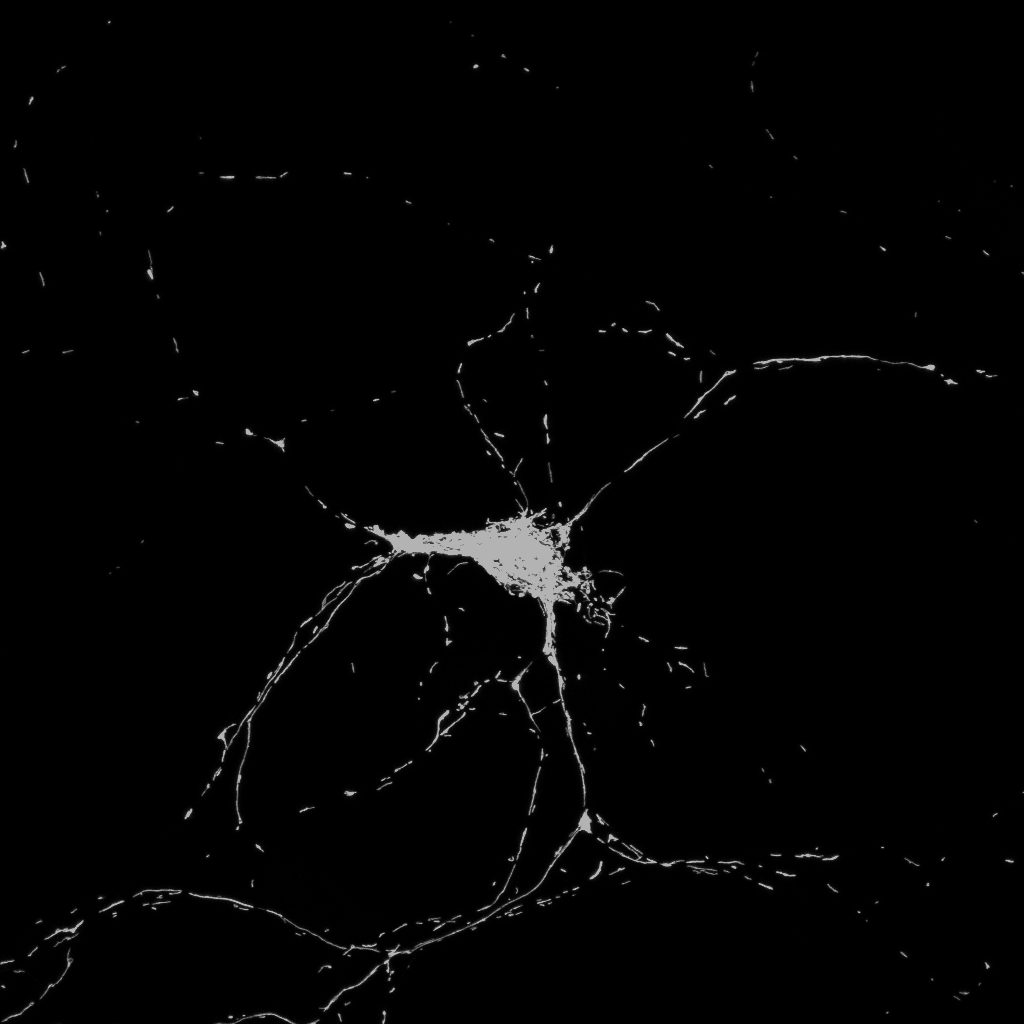

Supplement: Supplementary file 7 — Source Data Fig. 3 [file 44319_2023_48_MOESM7_ESM.zip › Figure 3/Figure 3E/UK sv9.tif]

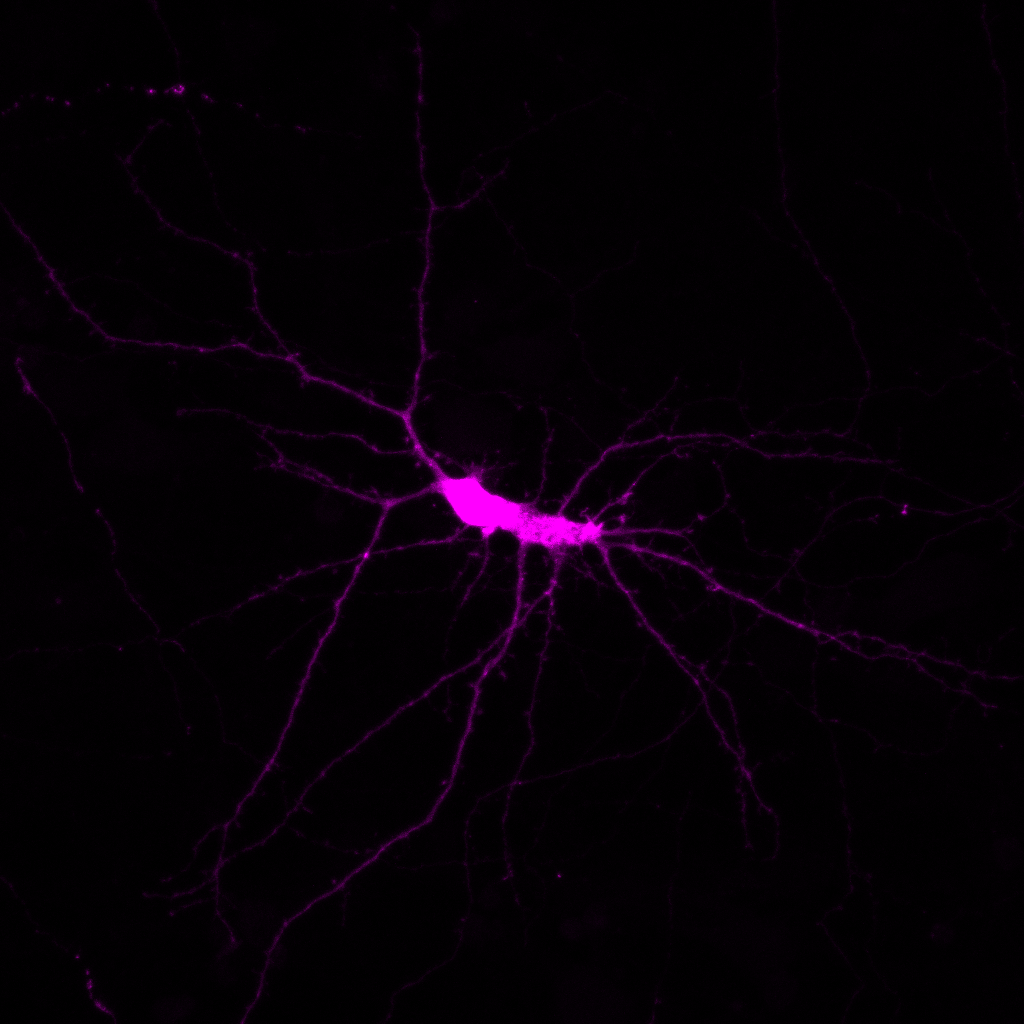

Supplement: Supplementary file 7 — Source Data Fig. 3 [file 44319_2023_48_MOESM7_ESM.zip › Figure 3/Figure 3E/DMSO mCherry.tif]

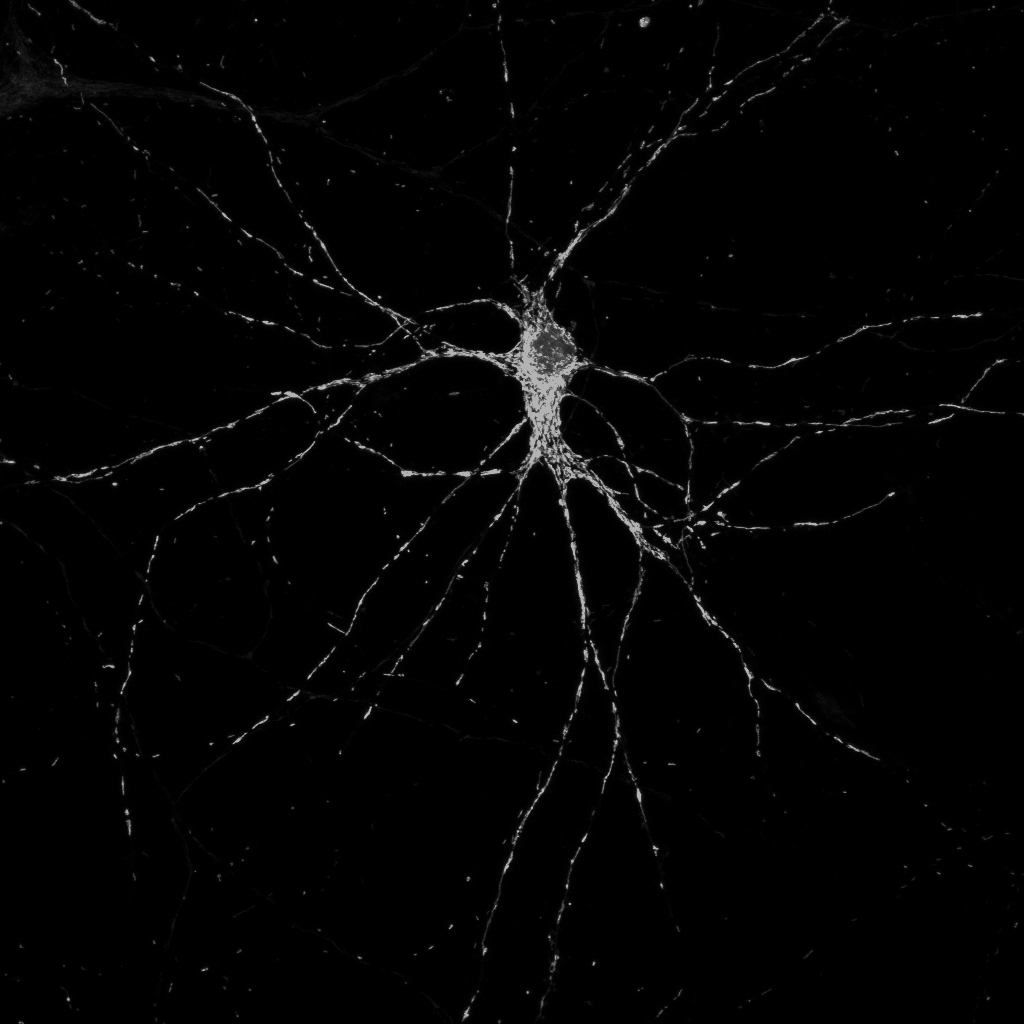

Supplement: Supplementary file 7 — Source Data Fig. 3 [file 44319_2023_48_MOESM7_ESM.zip › Figure 3/Figure 3C/sv9 Vehicle.tif]

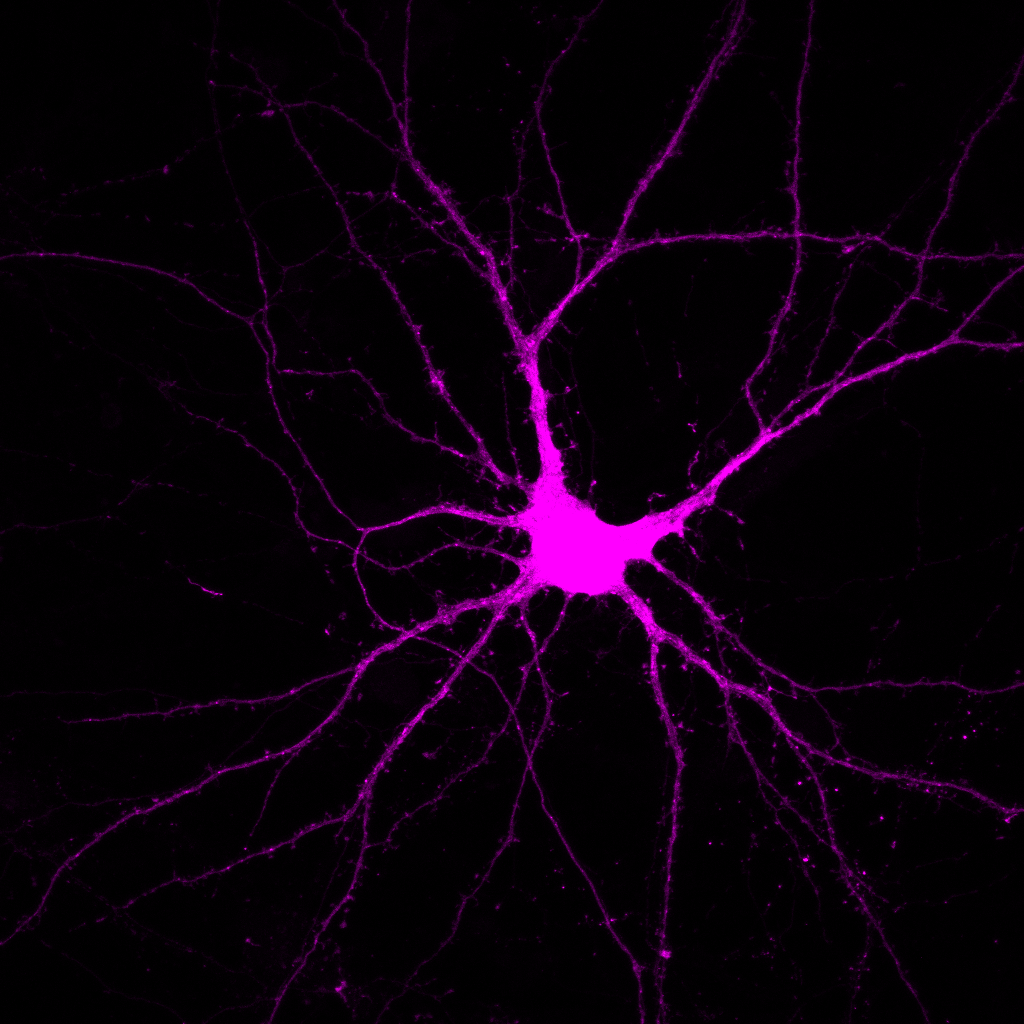

Supplement: Supplementary file 7 — Source Data Fig. 3 [file 44319_2023_48_MOESM7_ESM.zip › Figure 3/Figure 3C/mcherry Etomoxir.tif]

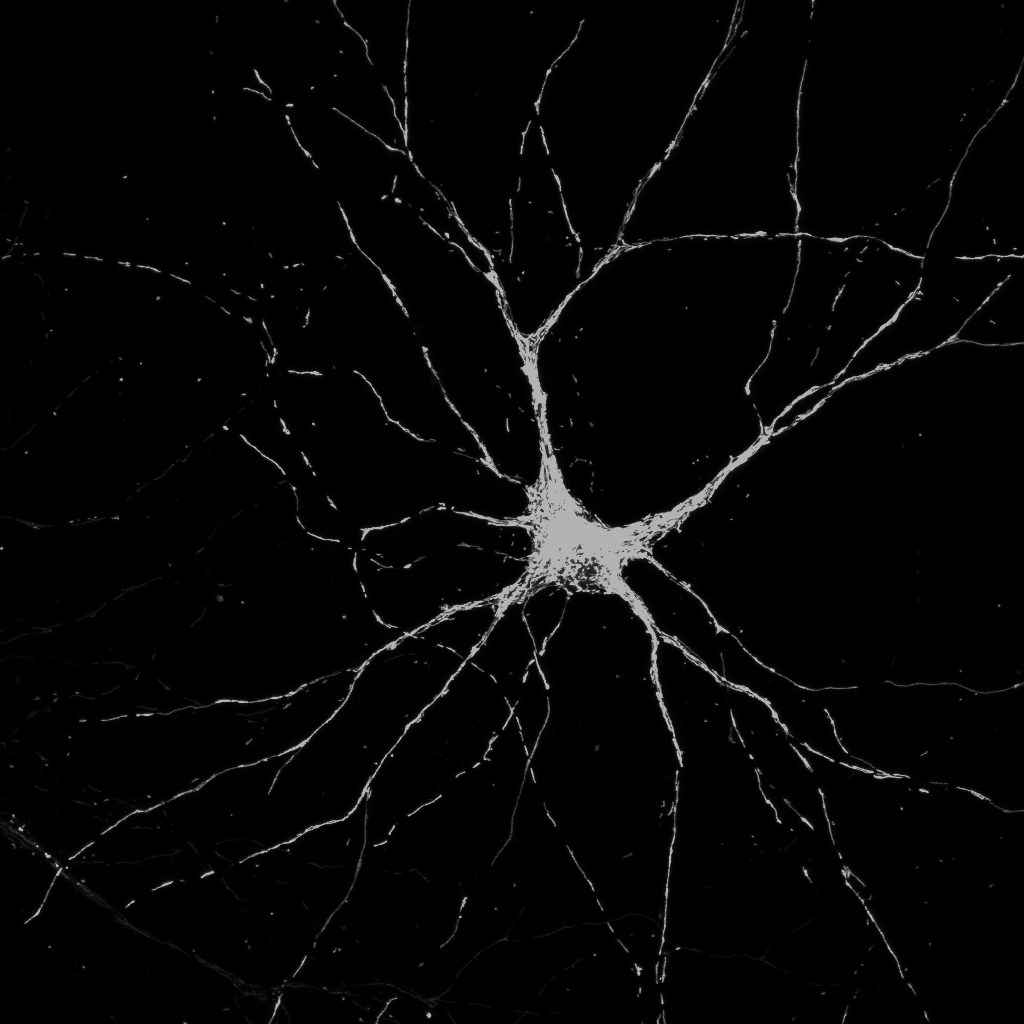

Supplement: Supplementary file 7 — Source Data Fig. 3 [file 44319_2023_48_MOESM7_ESM.zip › Figure 3/Figure 3C/sv9 Etomoxir.tif]

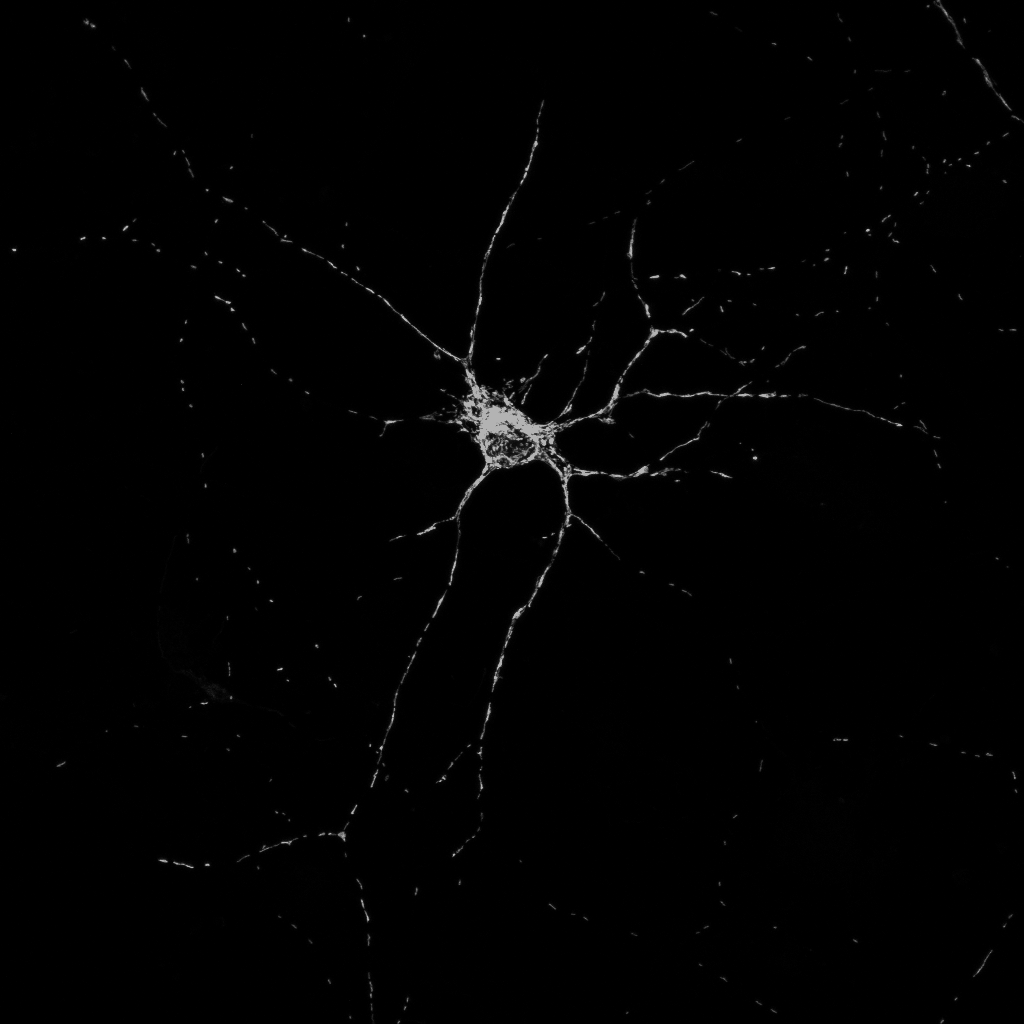

Supplement: Supplementary file 7 — Source Data Fig. 3 [file 44319_2023_48_MOESM7_ESM.zip › Figure 3/Figure 3D/DMSO sv9.tif]

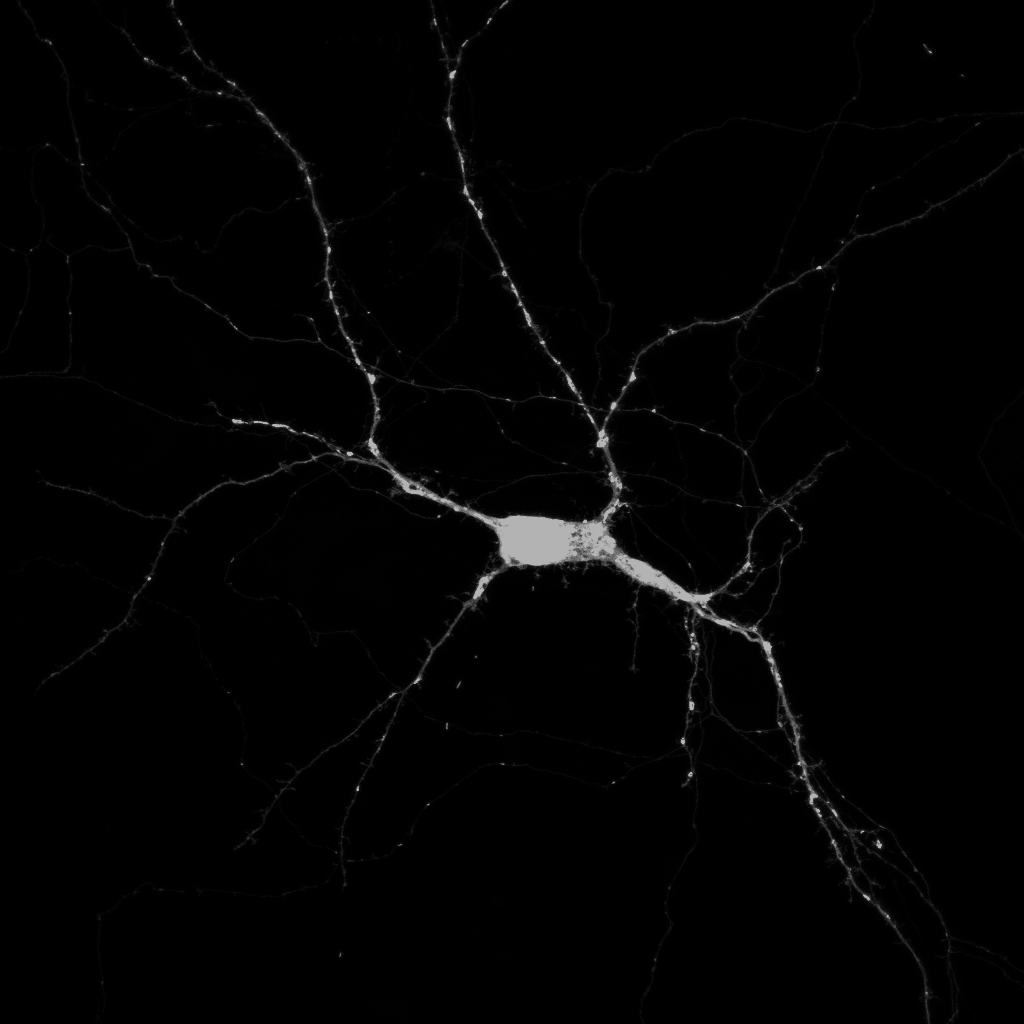

Supplement: Supplementary file 7 — Source Data Fig. 3 [file 44319_2023_48_MOESM7_ESM.zip › Figure 3/Figure 3D/CB839 SV9.tif]

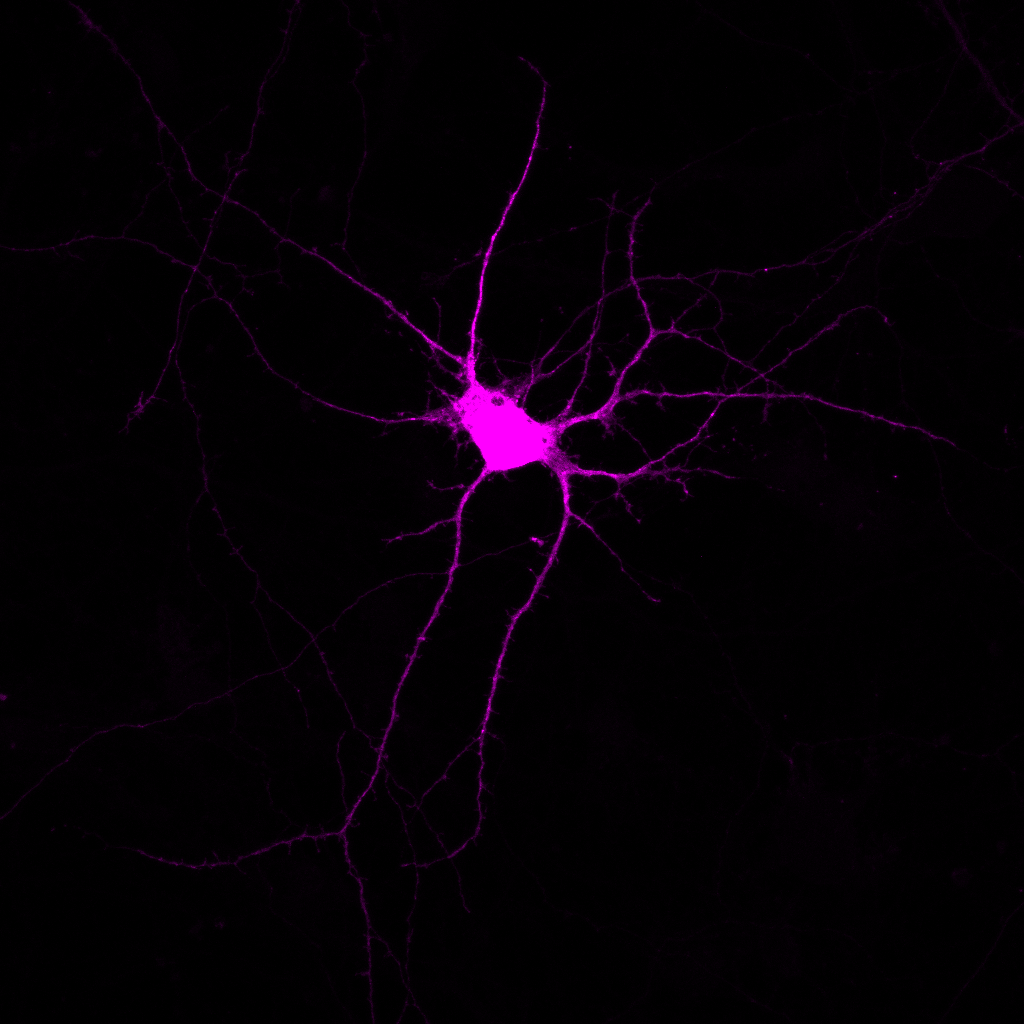

Supplement: Supplementary file 7 — Source Data Fig. 3 [file 44319_2023_48_MOESM7_ESM.zip › Figure 3/Figure 3D/DMSO mcherry.tif]

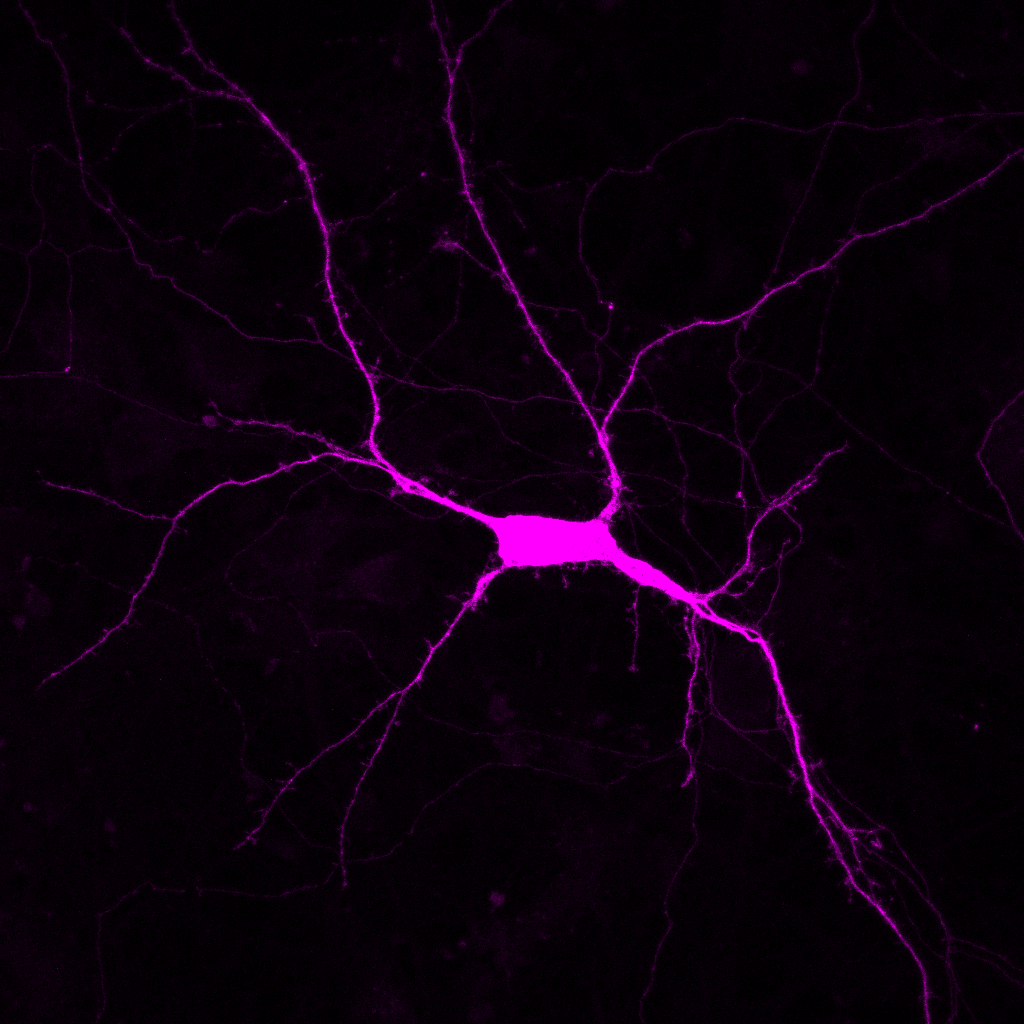

Supplement: Supplementary file 7 — Source Data Fig. 3 [file 44319_2023_48_MOESM7_ESM.zip › Figure 3/Figure 3D/CB839 mcherry.tif]

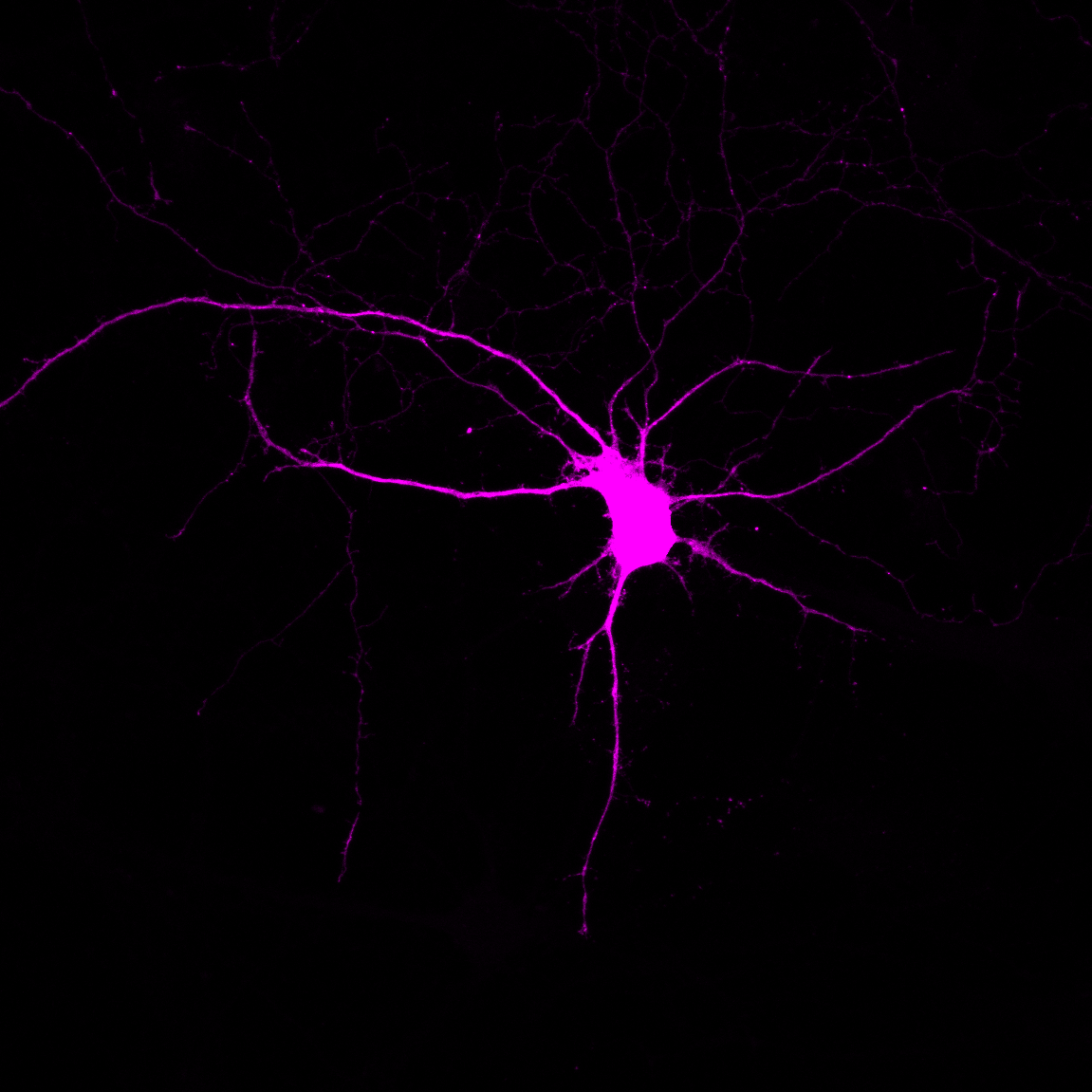

Supplement: Supplementary file 8 — Source Data Fig. 4 [file 44319_2023_48_MOESM8_ESM.zip › Figure 4/Figure 4H/UK mcherry.tif]

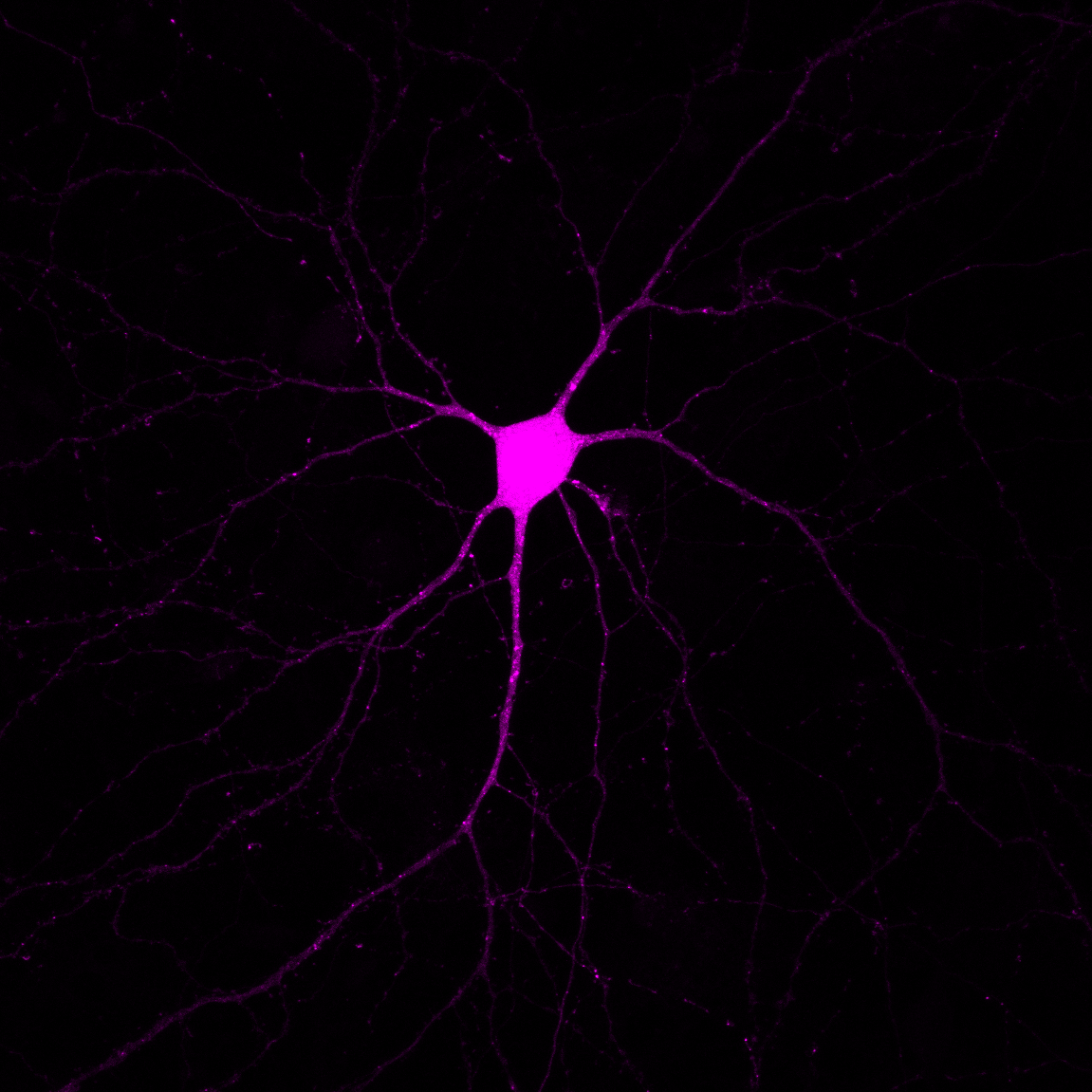

Supplement: Supplementary file 8 — Source Data Fig. 4 [file 44319_2023_48_MOESM8_ESM.zip › Figure 4/Figure 4H/DMSO mcherry.tif]

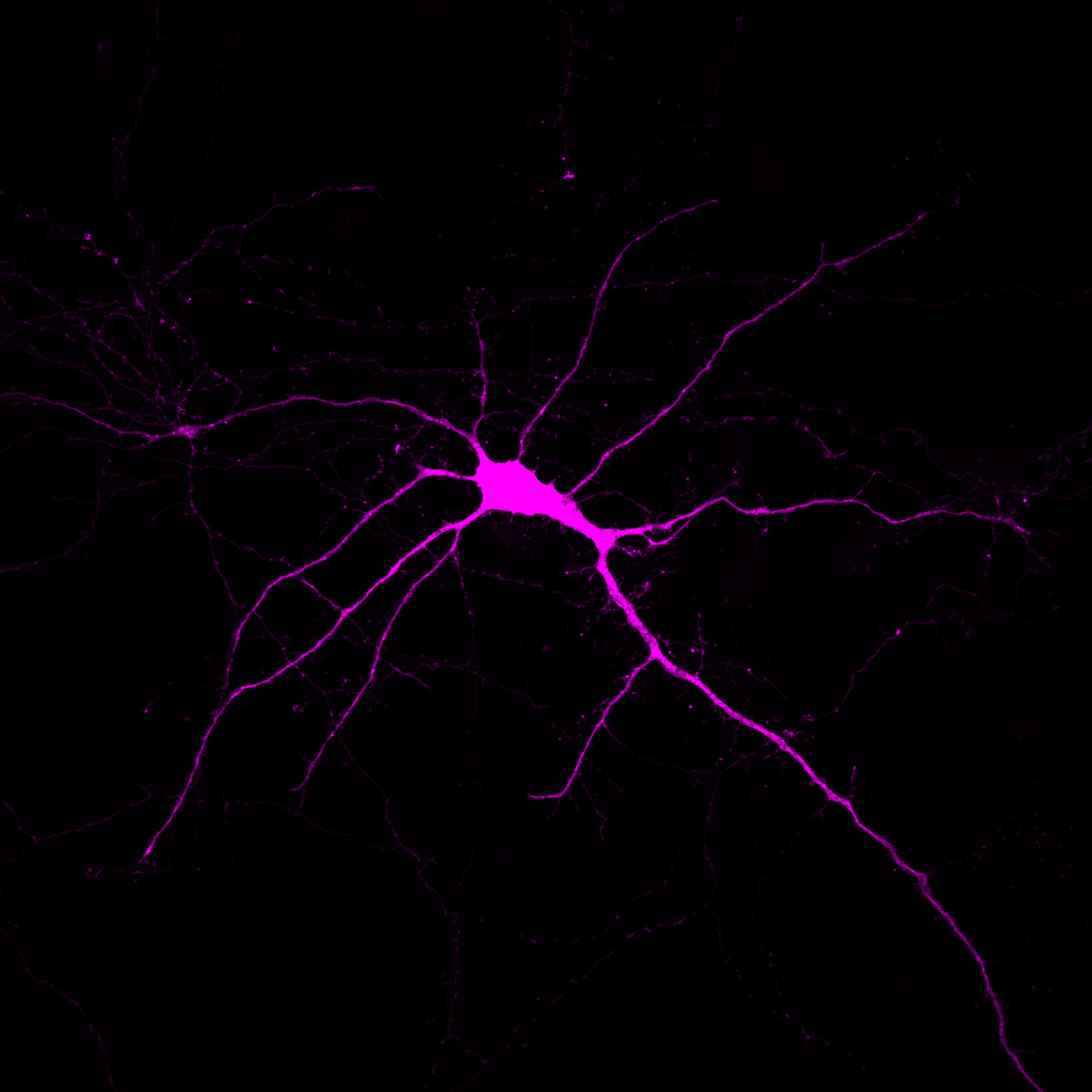

Supplement: Supplementary file 8 — Source Data Fig. 4 [file 44319_2023_48_MOESM8_ESM.zip › Figure 4/Figure 4H/UK piu aKG mcherry.tif]

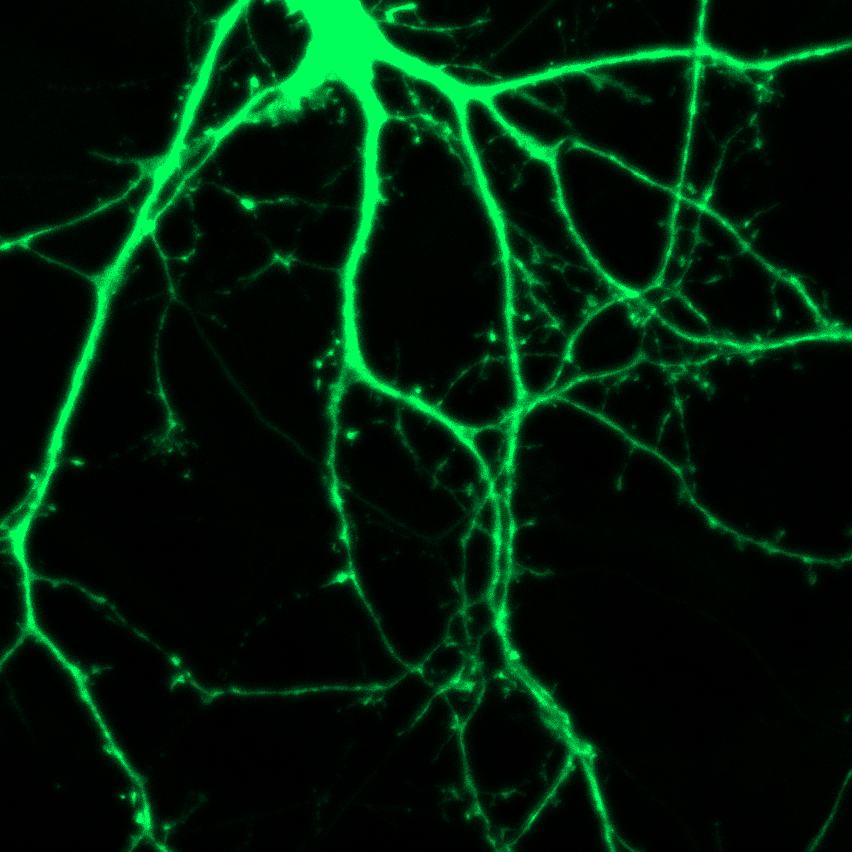

Supplement: Supplementary file 9 — Source Data Fig. 5 [file 44319_2023_48_MOESM9_ESM.zip › Figure 5/Figure 5C/UK5099 + Glu GFP.tif]

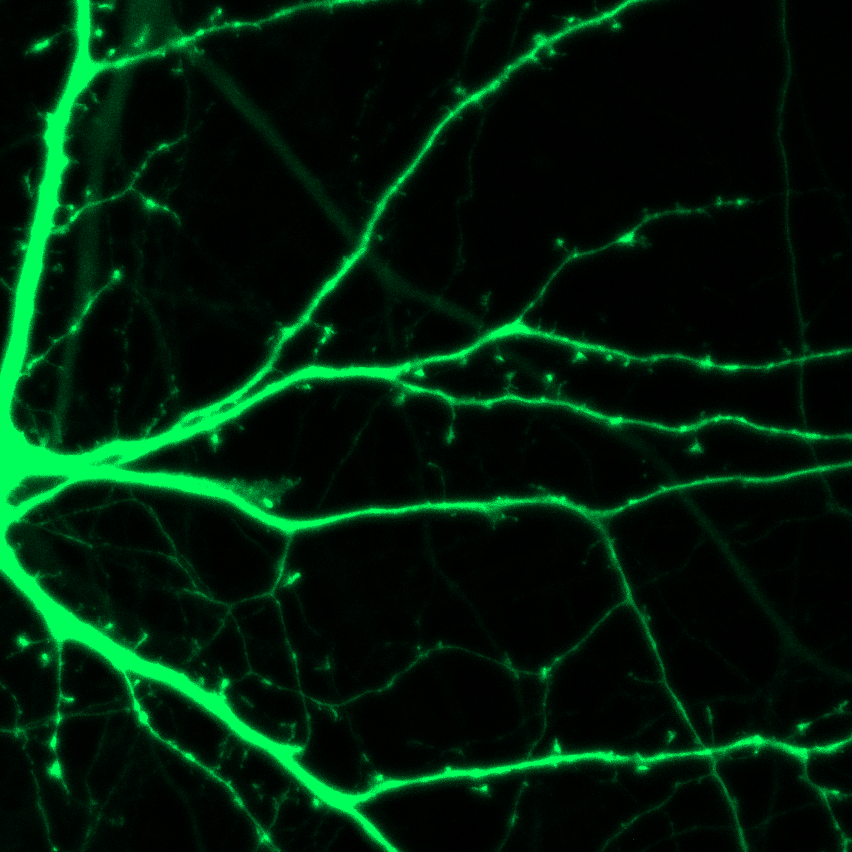

Supplement: Supplementary file 9 — Source Data Fig. 5 [file 44319_2023_48_MOESM9_ESM.zip › Figure 5/Figure 5C/UK5099 GFP.tif]

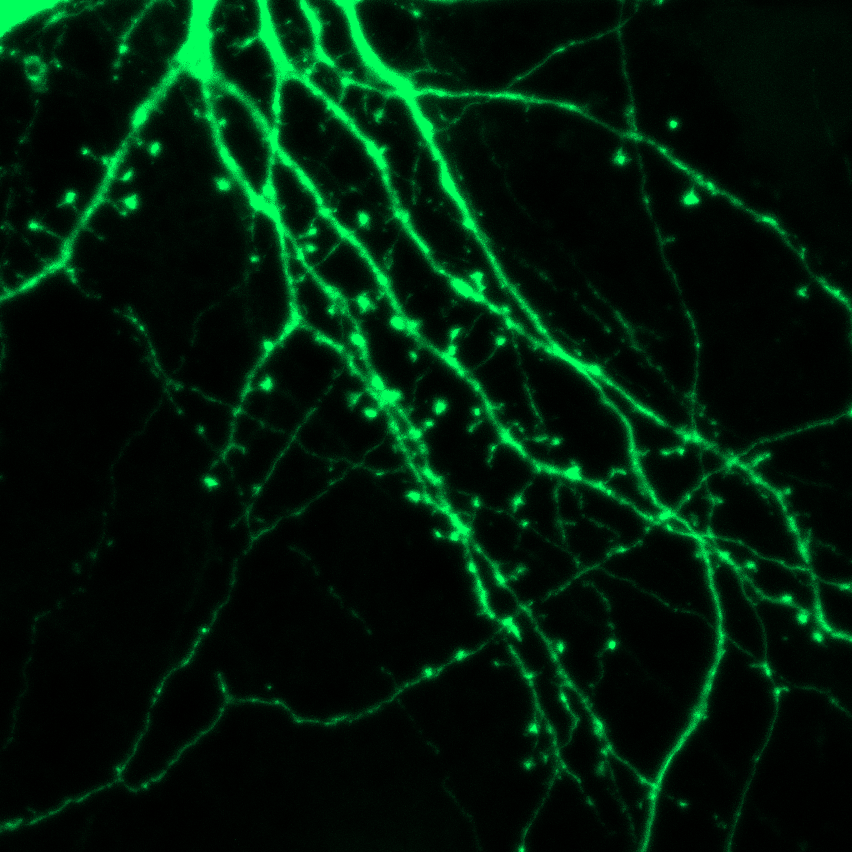

Supplement: Supplementary file 9 — Source Data Fig. 5 [file 44319_2023_48_MOESM9_ESM.zip › Figure 5/Figure 5C/DMSO GFP.tif]

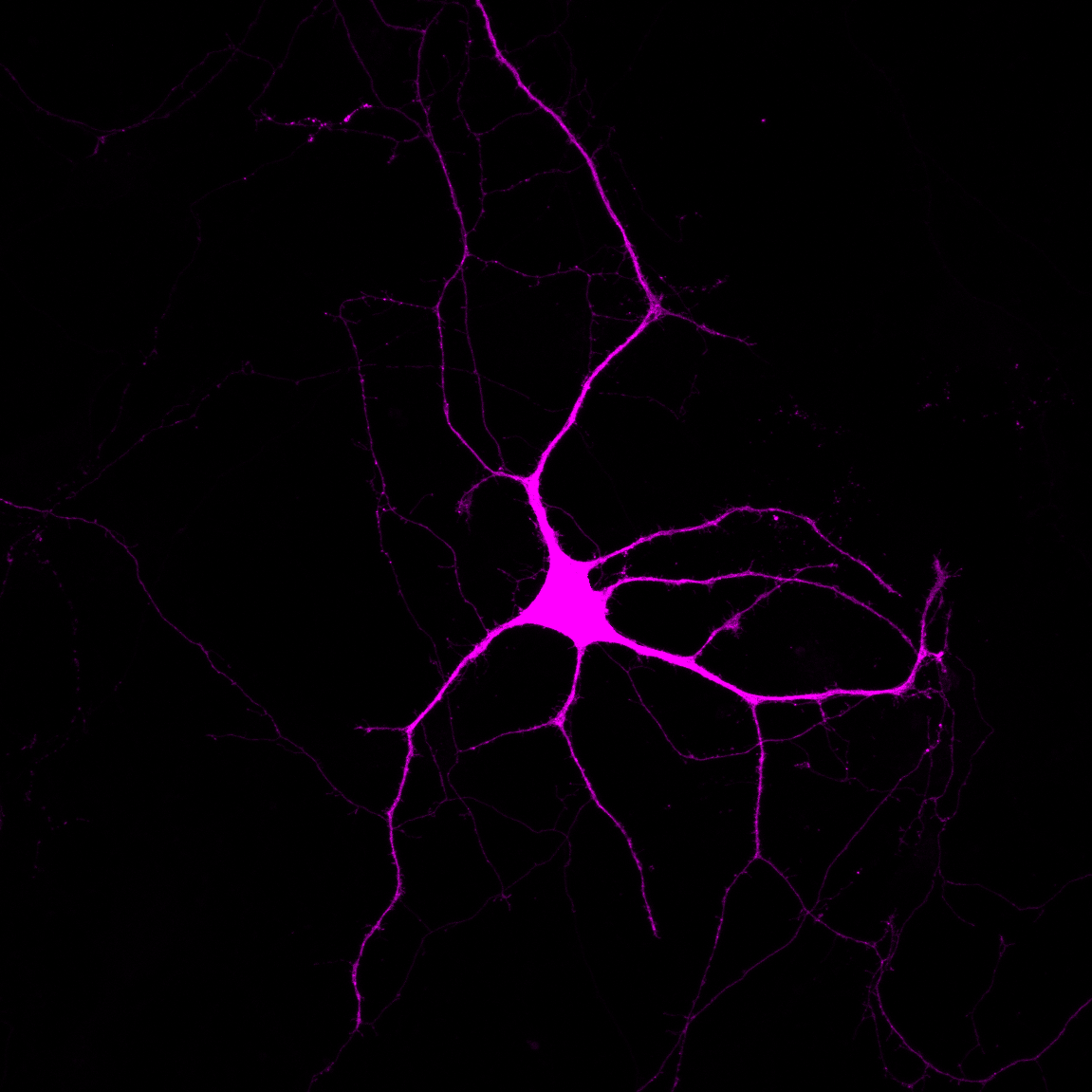

Supplement: Supplementary file 9 — Source Data Fig. 5 [file 44319_2023_48_MOESM9_ESM.zip › Figure 5/Figure 5B/UK mcherry.tif]

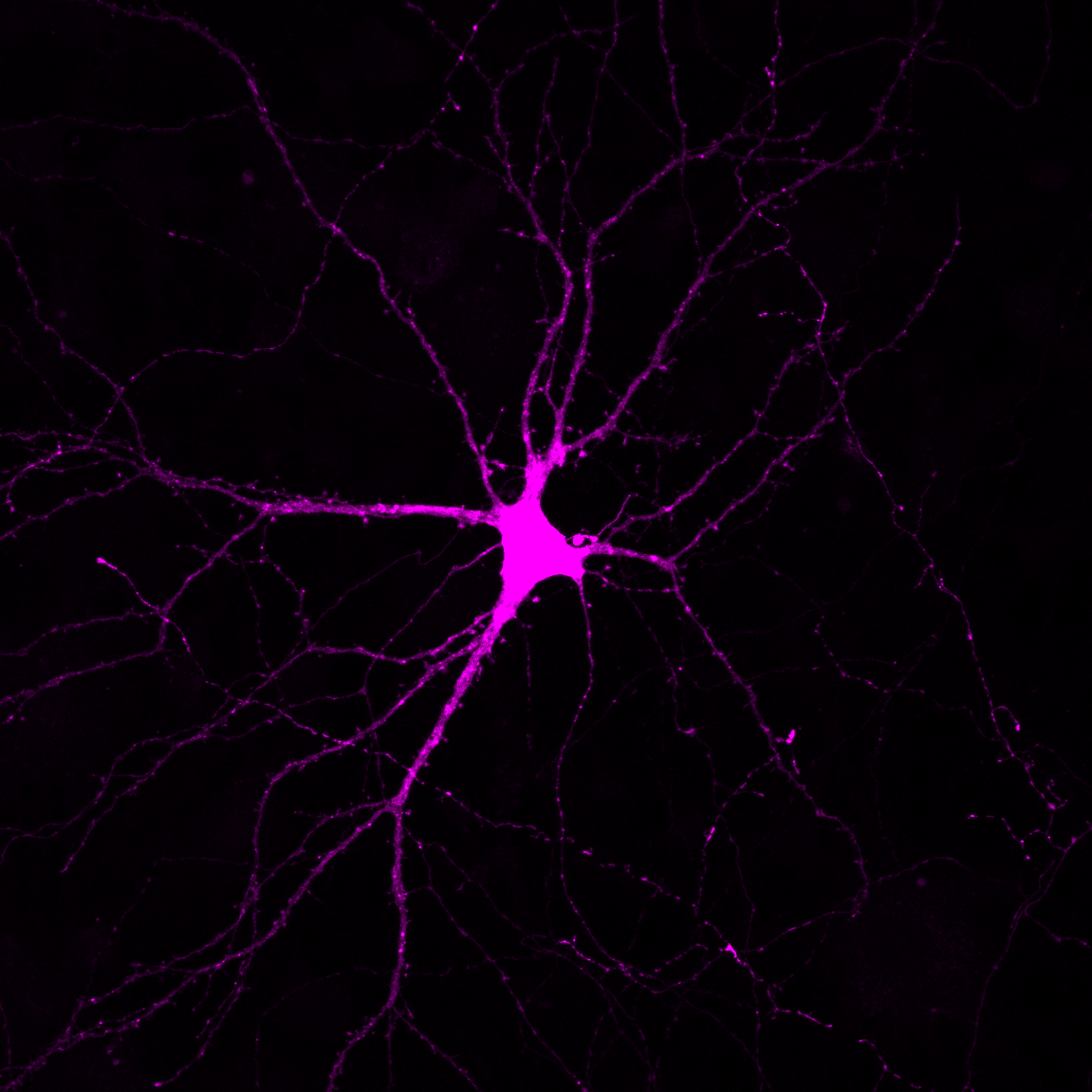

Supplement: Supplementary file 9 — Source Data Fig. 5 [file 44319_2023_48_MOESM9_ESM.zip › Figure 5/Figure 5B/DMSO mcherry.tif]

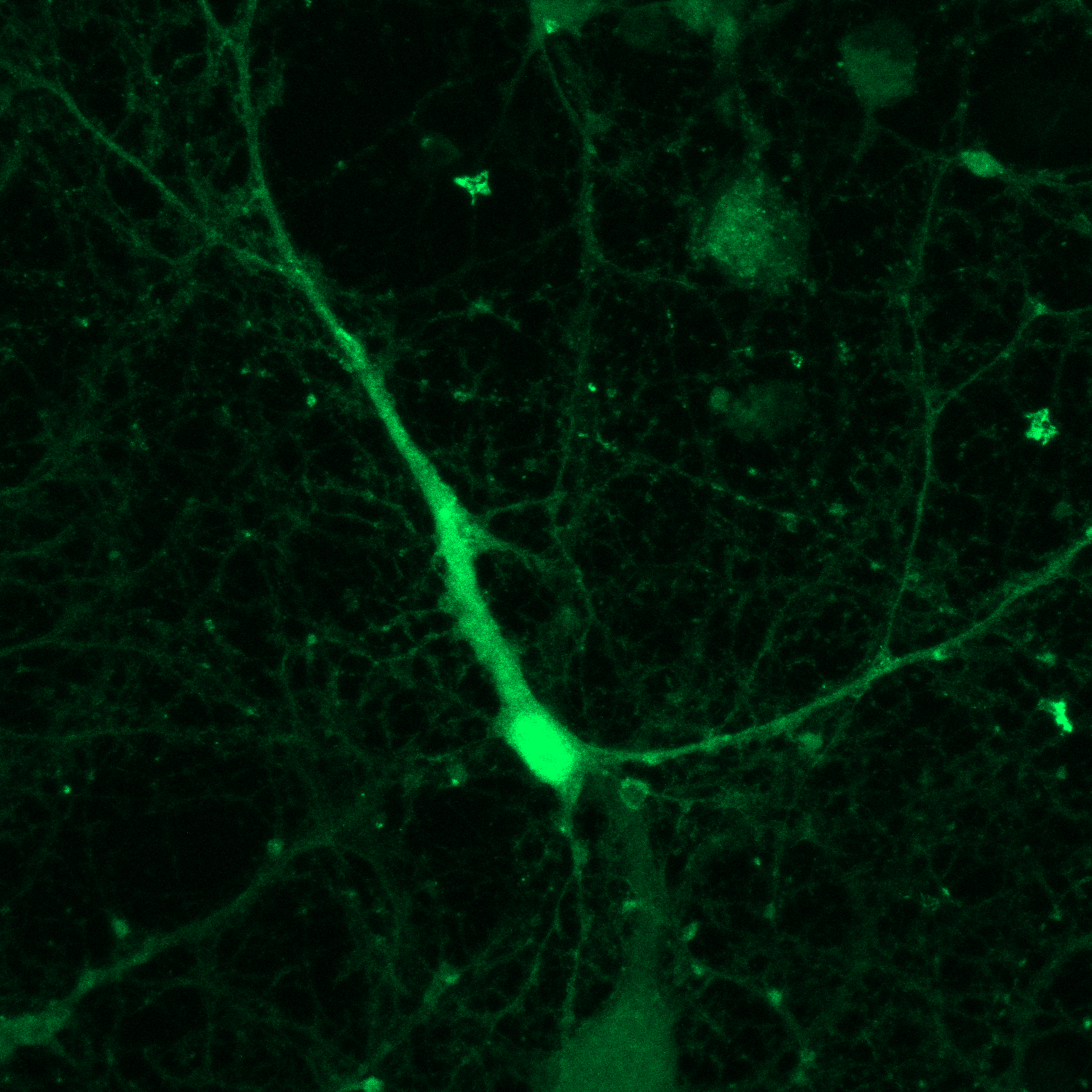

Supplement: Supplementary file 10 — Source Data Fig. 6 [file 44319_2023_48_MOESM10_ESM.zip › Figure 6/Figure 6D/Confocal image Sh MPC1.tif]

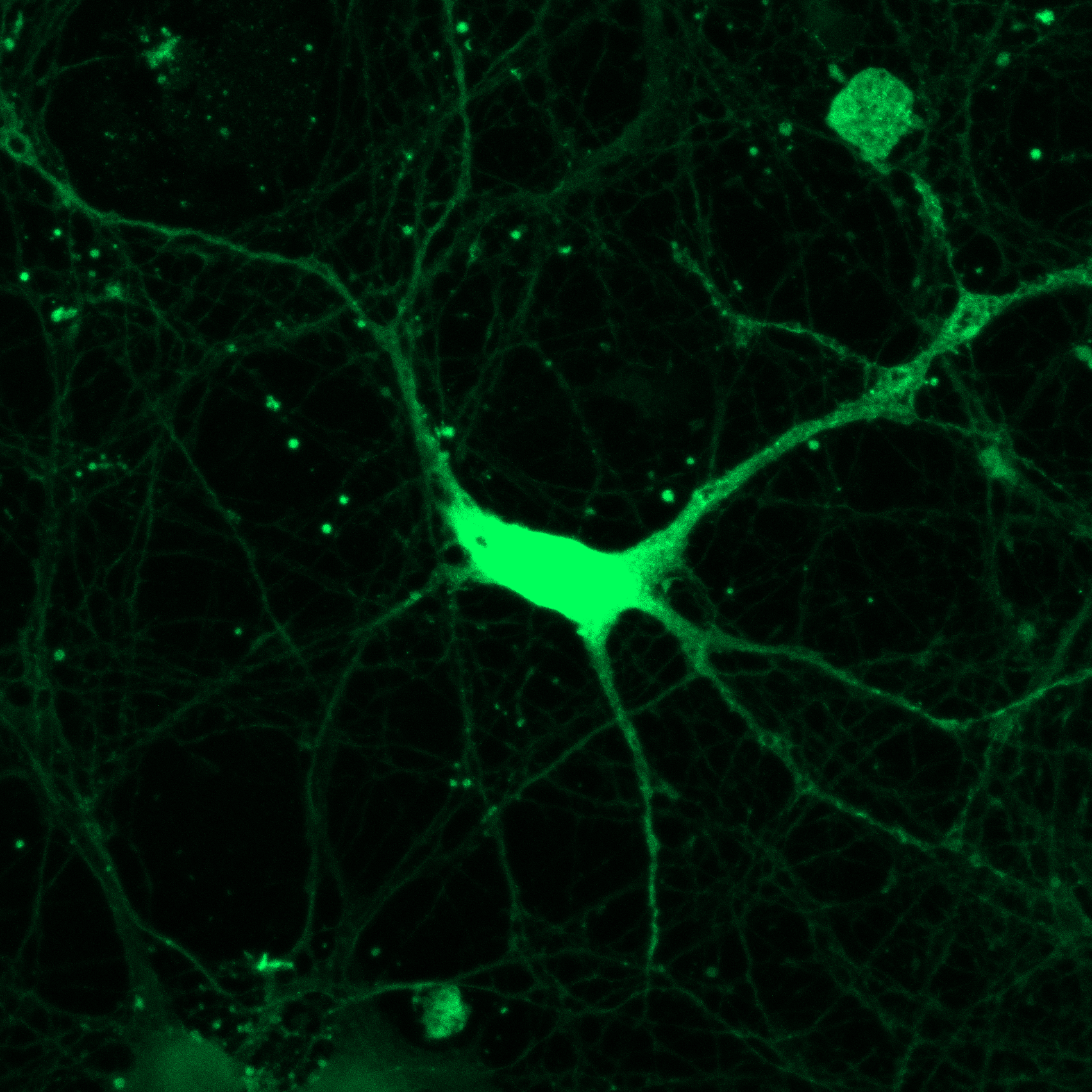

Supplement: Supplementary file 10 — Source Data Fig. 6 [file 44319_2023_48_MOESM10_ESM.zip › Figure 6/Figure 6D/Confocal image SCR.tif]

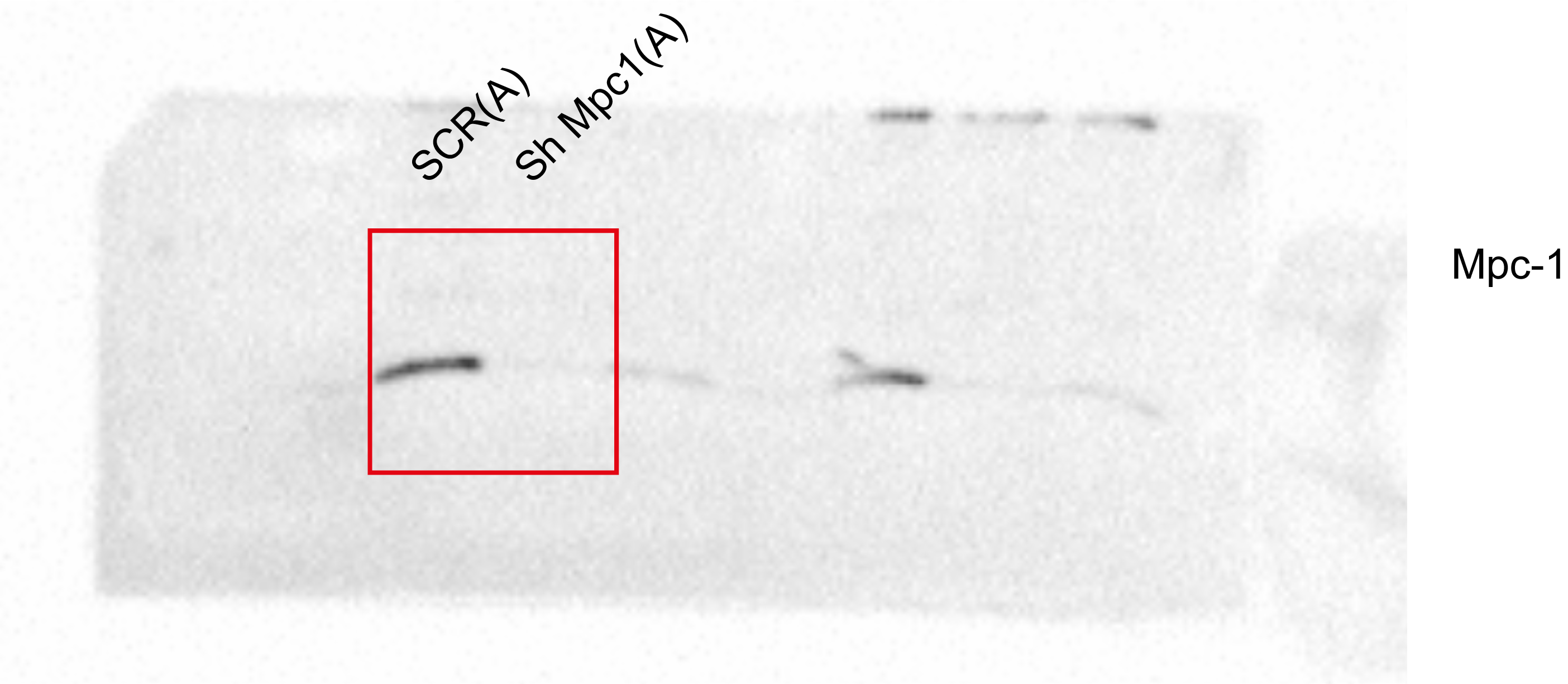

Supplement: Supplementary file 10 — Source Data Fig. 6 [file 44319_2023_48_MOESM10_ESM.zip › Figure 6/Figure 6C/Western Mpc-1.tif]

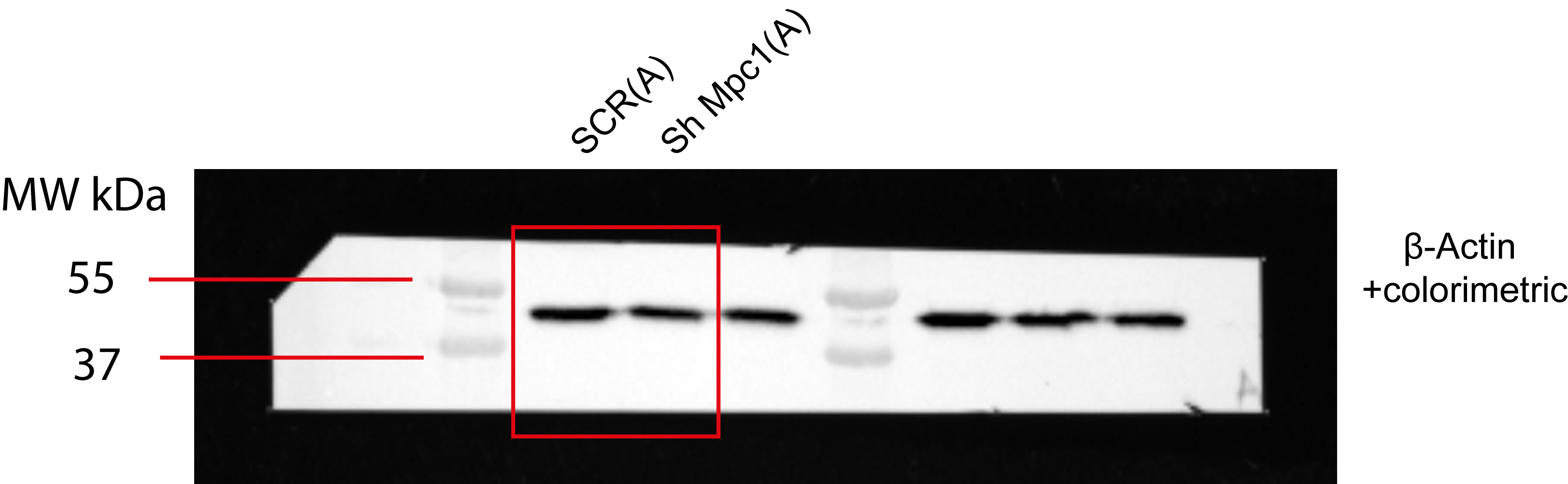

Supplement: Supplementary file 10 — Source Data Fig. 6 [file 44319_2023_48_MOESM10_ESM.zip › Figure 6/Figure 6C/Western Actin.tif]

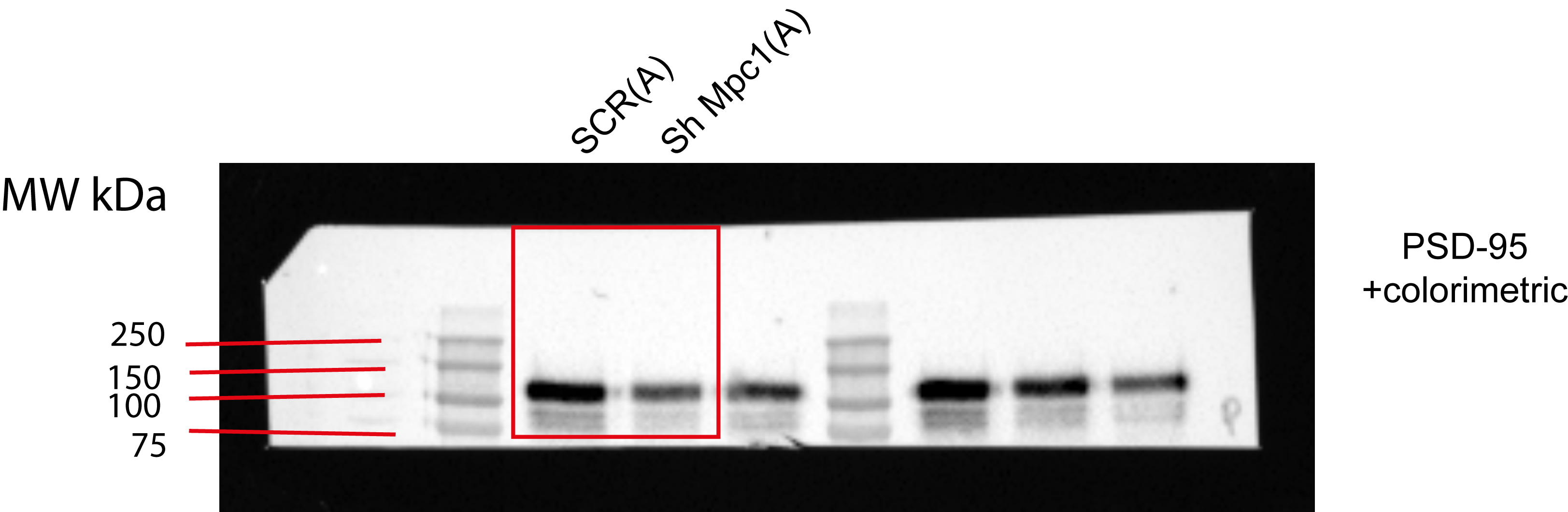

Supplement: Supplementary file 10 — Source Data Fig. 6 [file 44319_2023_48_MOESM10_ESM.zip › Figure 6/Figure 6C/Western PSD95.tif]

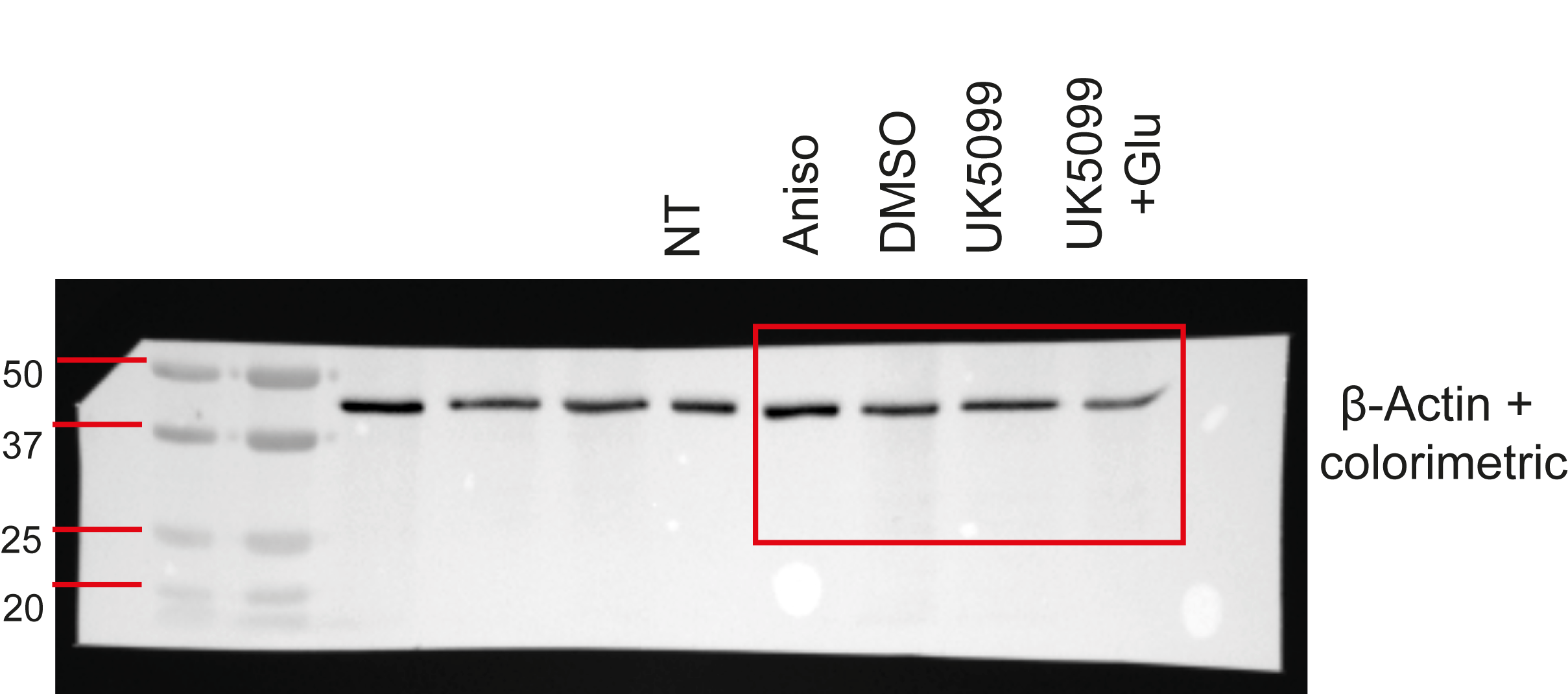

Supplement: Supplementary file 11 — Source Data Fig. 7 [file 44319_2023_48_MOESM11_ESM.zip › Figure 7/Figure 7D/Western Actin.tif]

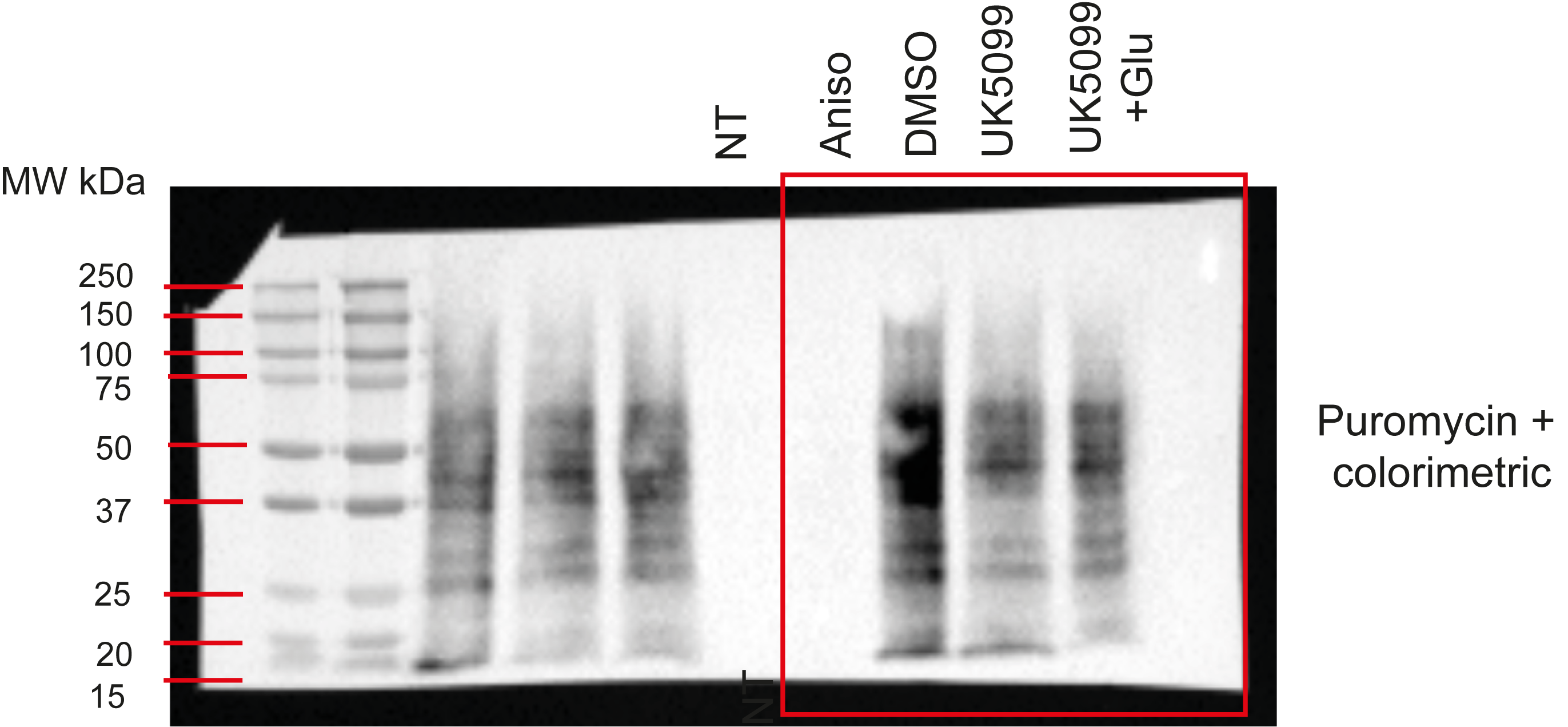

Supplement: Supplementary file 11 — Source Data Fig. 7 [file 44319_2023_48_MOESM11_ESM.zip › Figure 7/Figure 7D/Western Puromycin.tif]

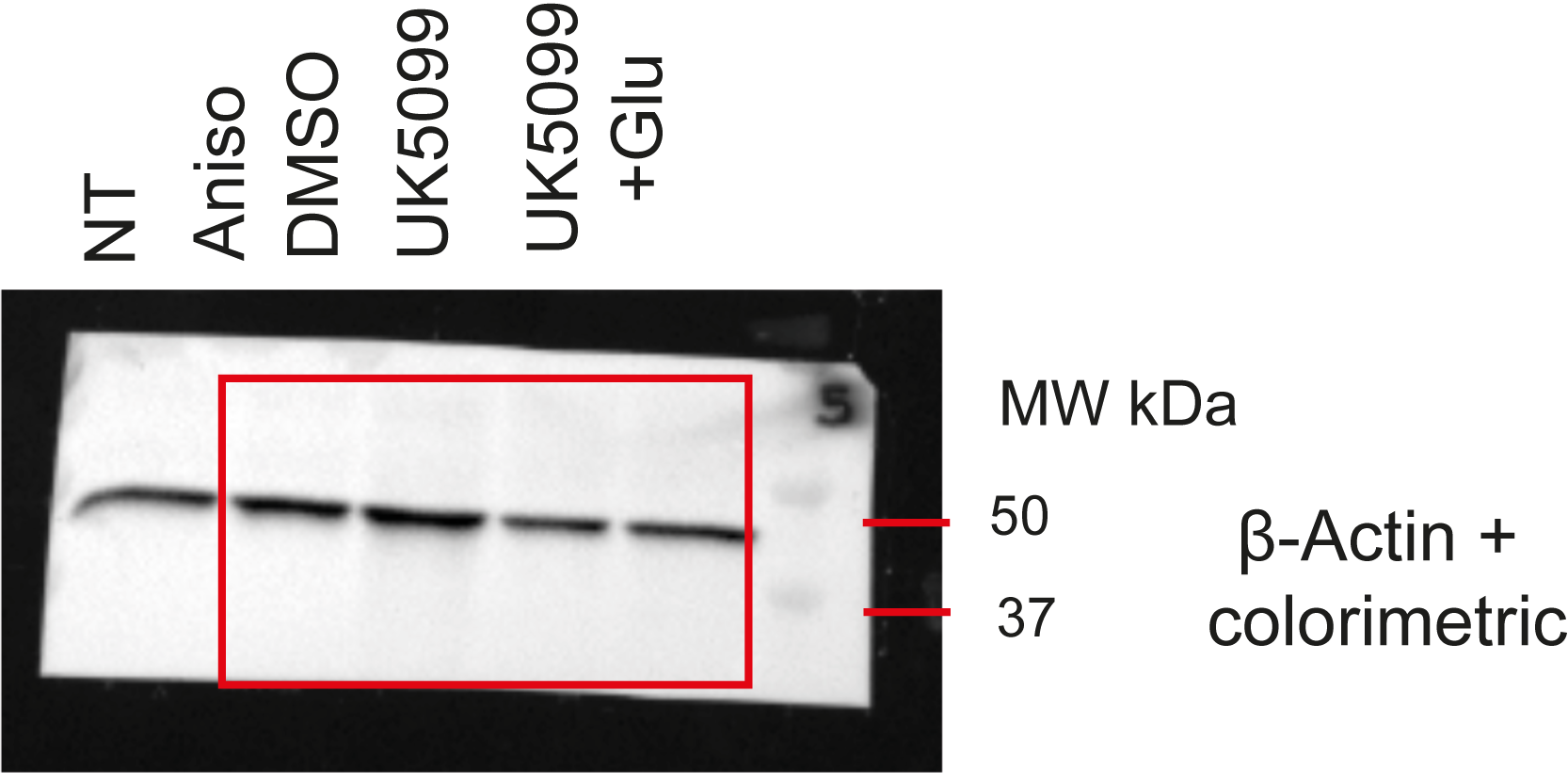

Supplement: Supplementary file 11 — Source Data Fig. 7 [file 44319_2023_48_MOESM11_ESM.zip › Figure 7/Figure 7C/Western Actin.tif]

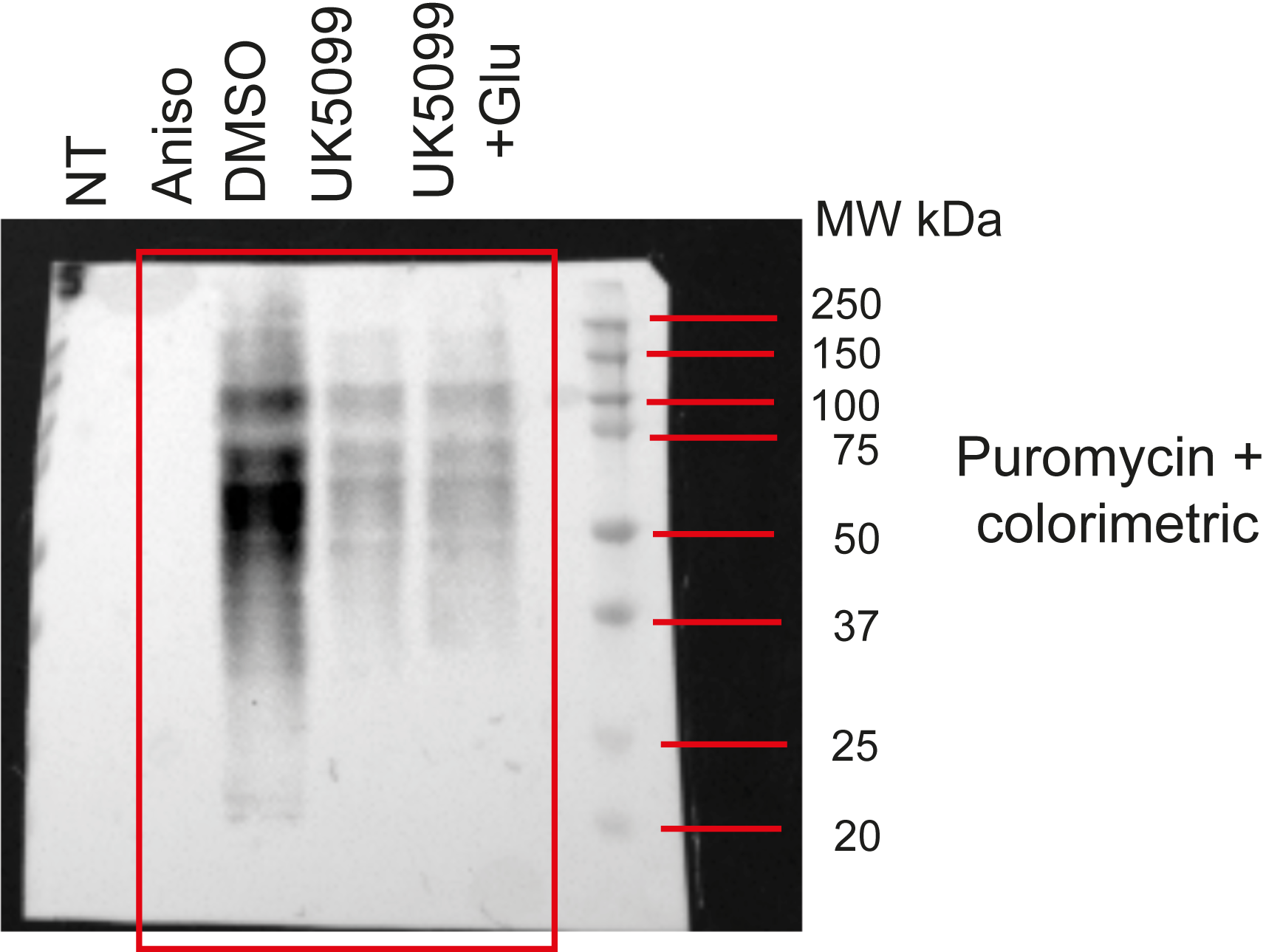

Supplement: Supplementary file 11 — Source Data Fig. 7 [file 44319_2023_48_MOESM11_ESM.zip › Figure 7/Figure 7C/Western puromycin.tif]

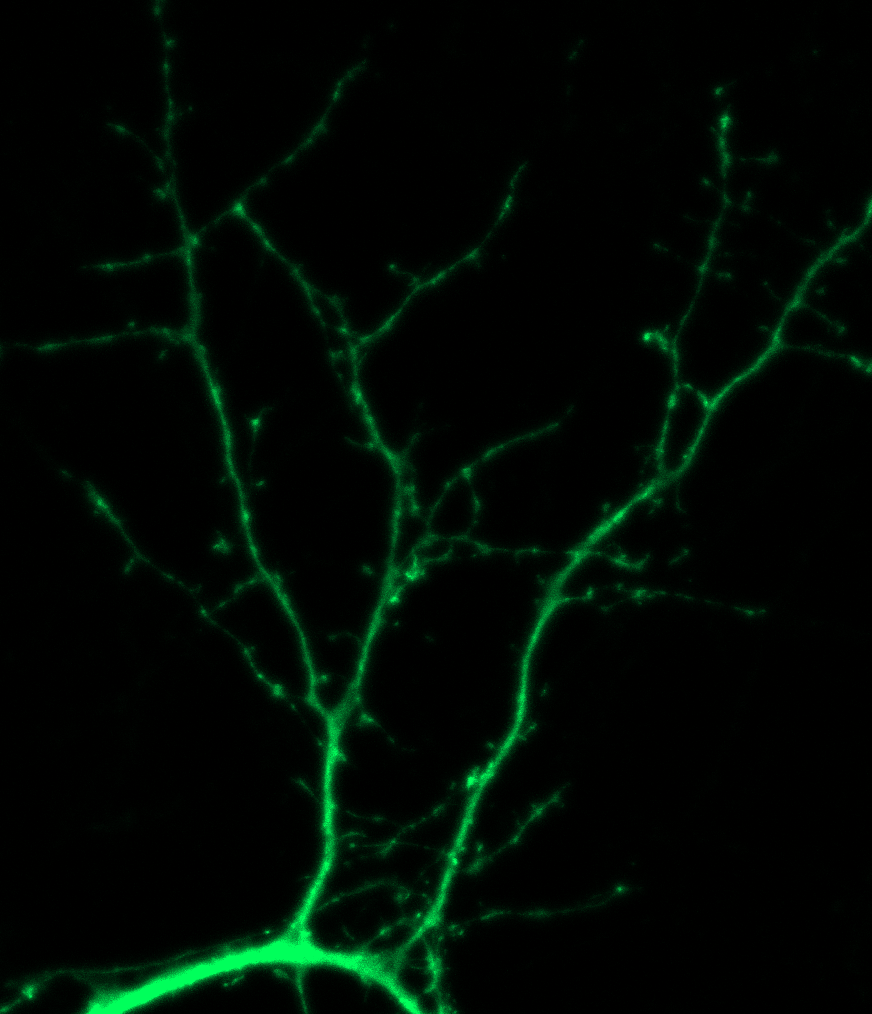

Supplement: Supplementary file 11 — Source Data Fig. 7 [file 44319_2023_48_MOESM11_ESM.zip › Figure 7/Figure 7E/UK GFP.tif]

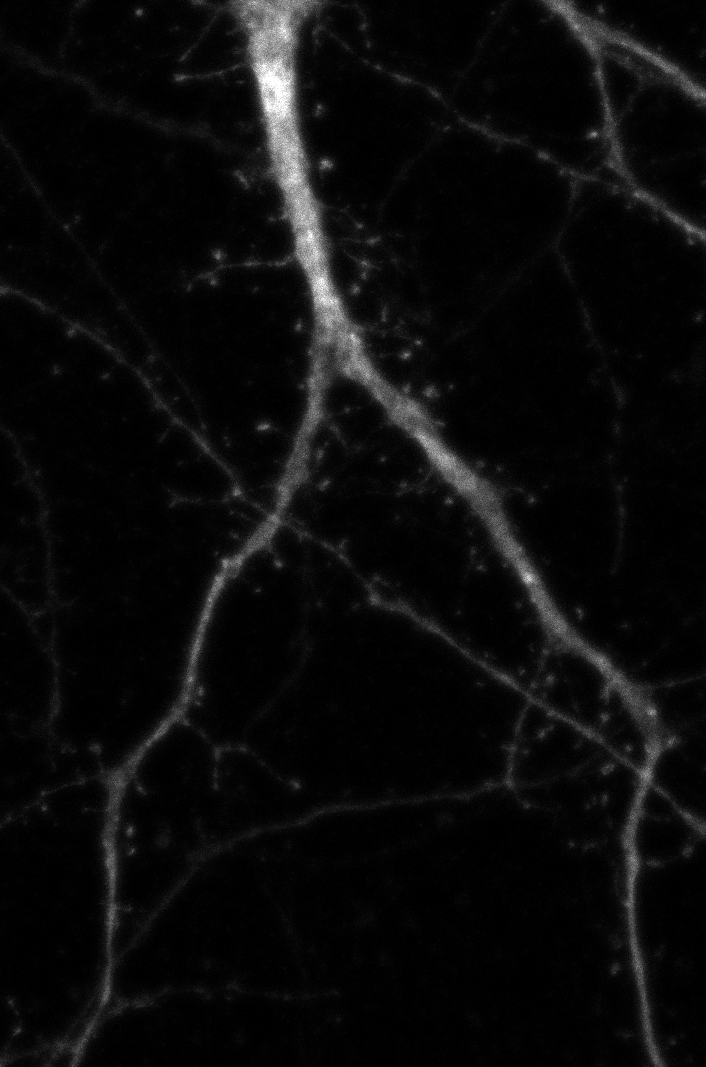

Supplement: Supplementary file 11 — Source Data Fig. 7 [file 44319_2023_48_MOESM11_ESM.zip › Figure 7/Figure 7E/DMSO Puromycin.tif]

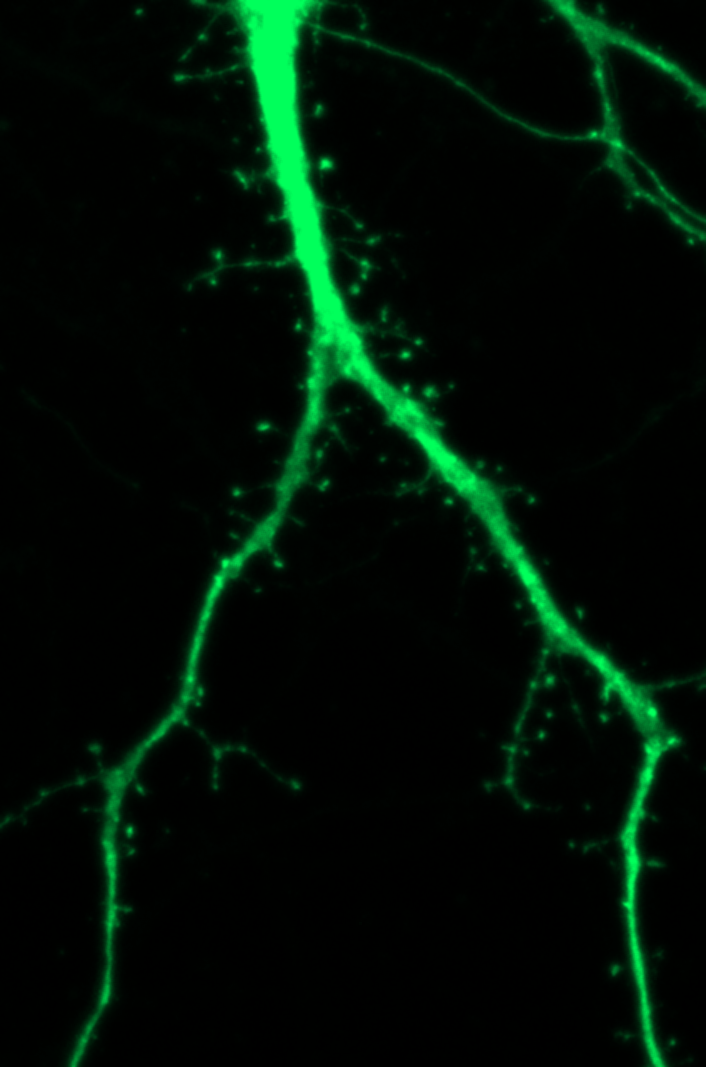

Supplement: Supplementary file 11 — Source Data Fig. 7 [file 44319_2023_48_MOESM11_ESM.zip › Figure 7/Figure 7E/DMSO GFP.tif]

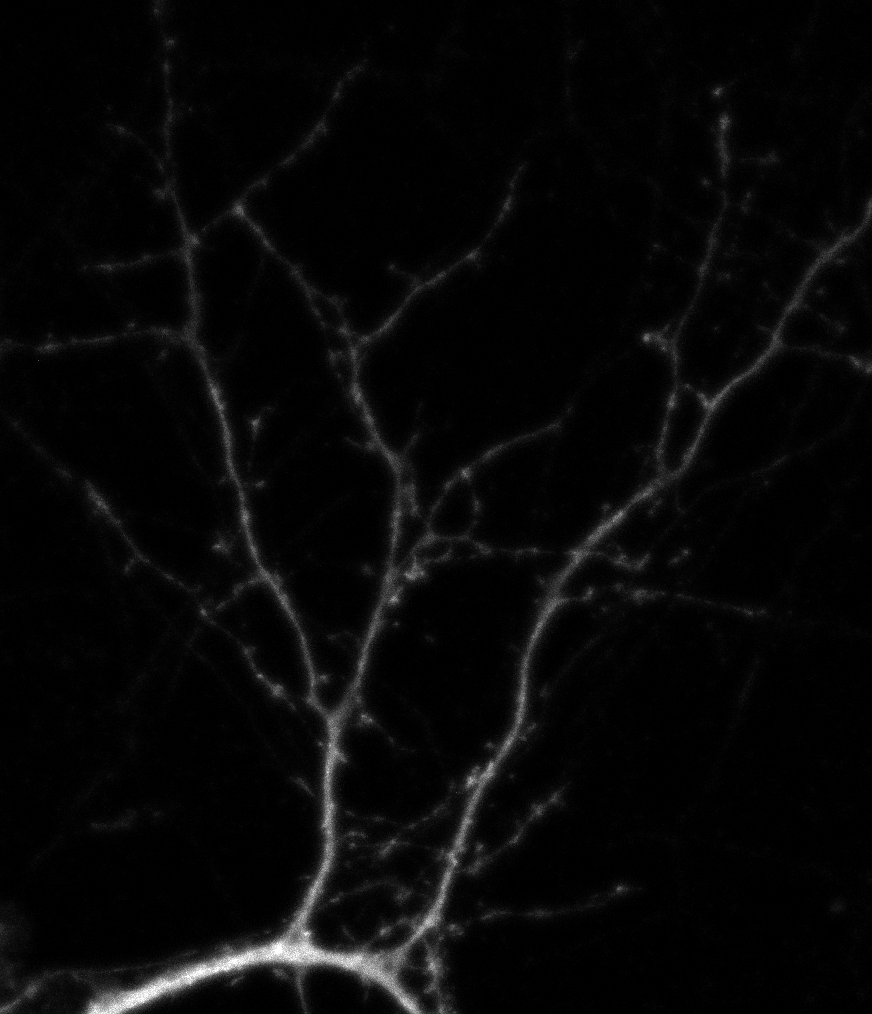

Supplement: Supplementary file 11 — Source Data Fig. 7 [file 44319_2023_48_MOESM11_ESM.zip › Figure 7/Figure 7E/UK puromycin.tif]

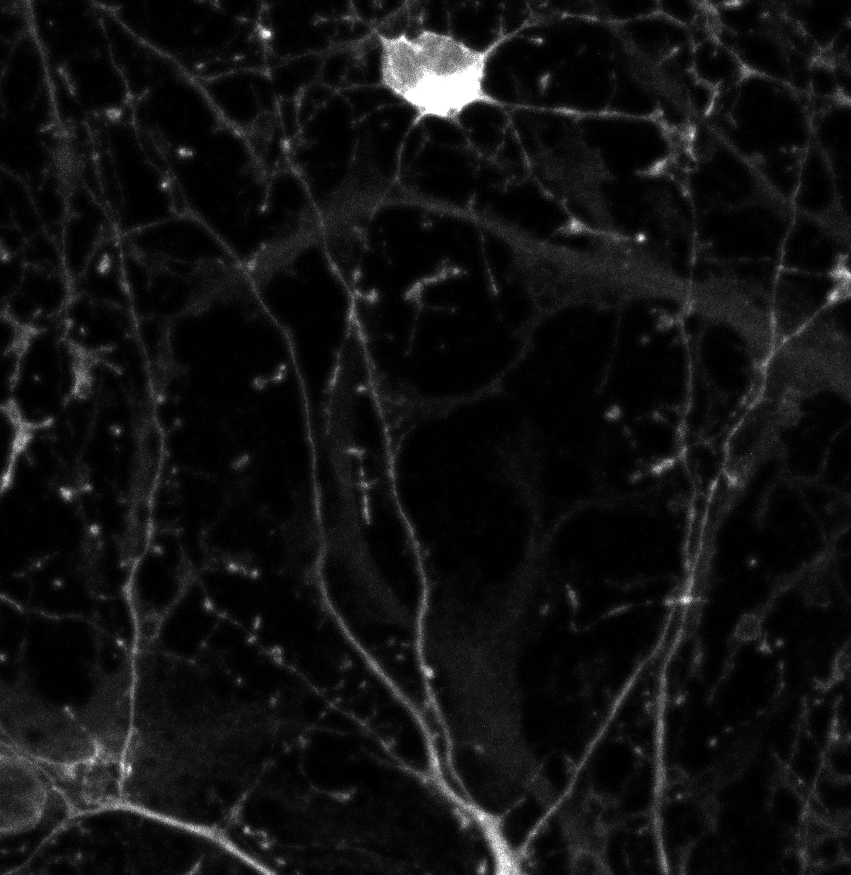

Supplement: Supplementary file 11 — Source Data Fig. 7 [file 44319_2023_48_MOESM11_ESM.zip › Figure 7/Figure 7E/UK+glu puromycin.tif]

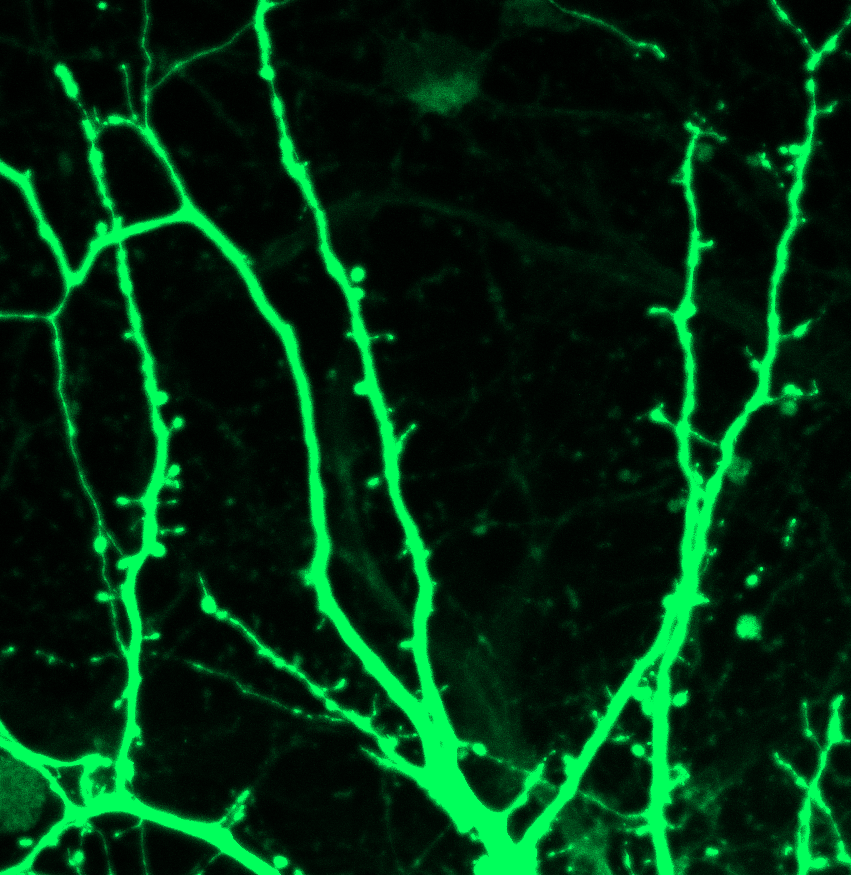

Supplement: Supplementary file 11 — Source Data Fig. 7 [file 44319_2023_48_MOESM11_ESM.zip › Figure 7/Figure 7E/UK+glu GFP.tif]

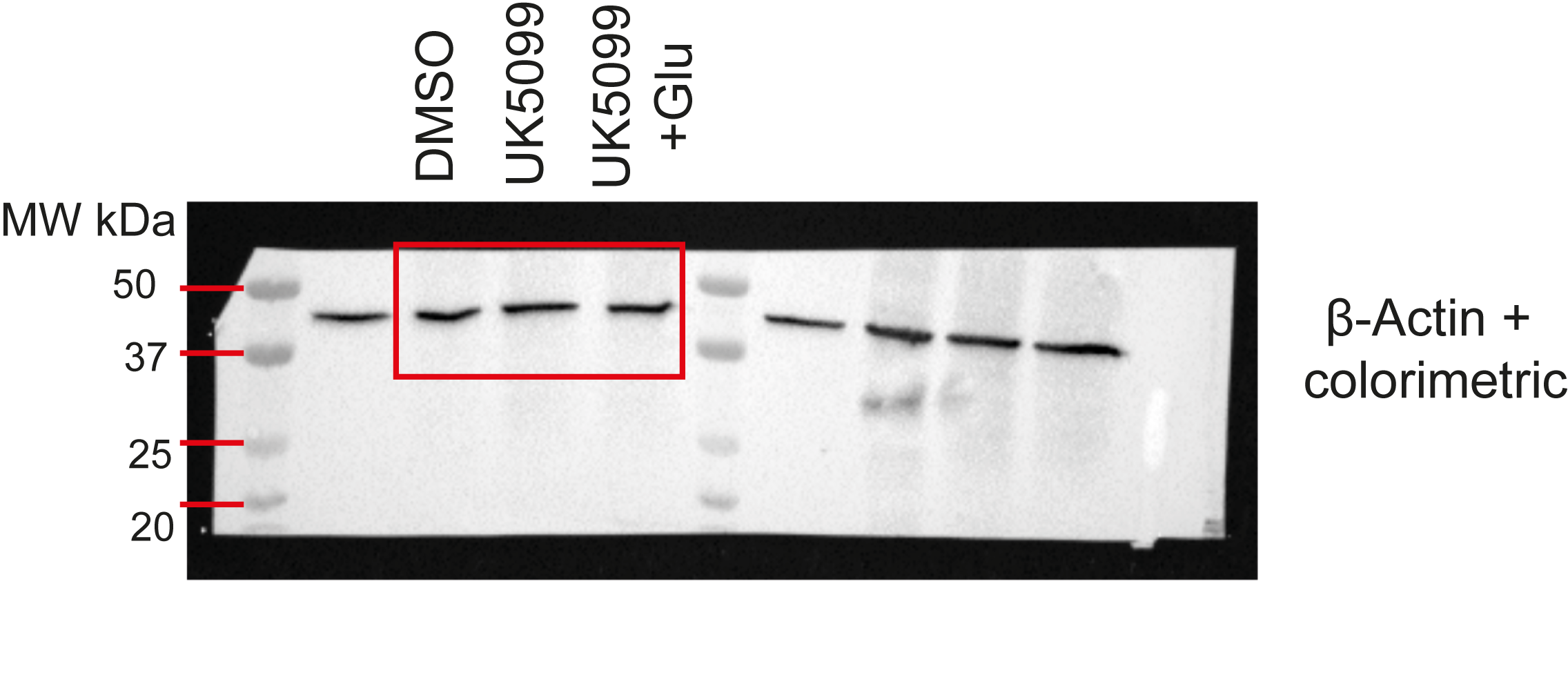

Supplement: Supplementary file 11 — Source Data Fig. 7 [file 44319_2023_48_MOESM11_ESM.zip › Figure 7/Figure 7F/Western Actin.tif]

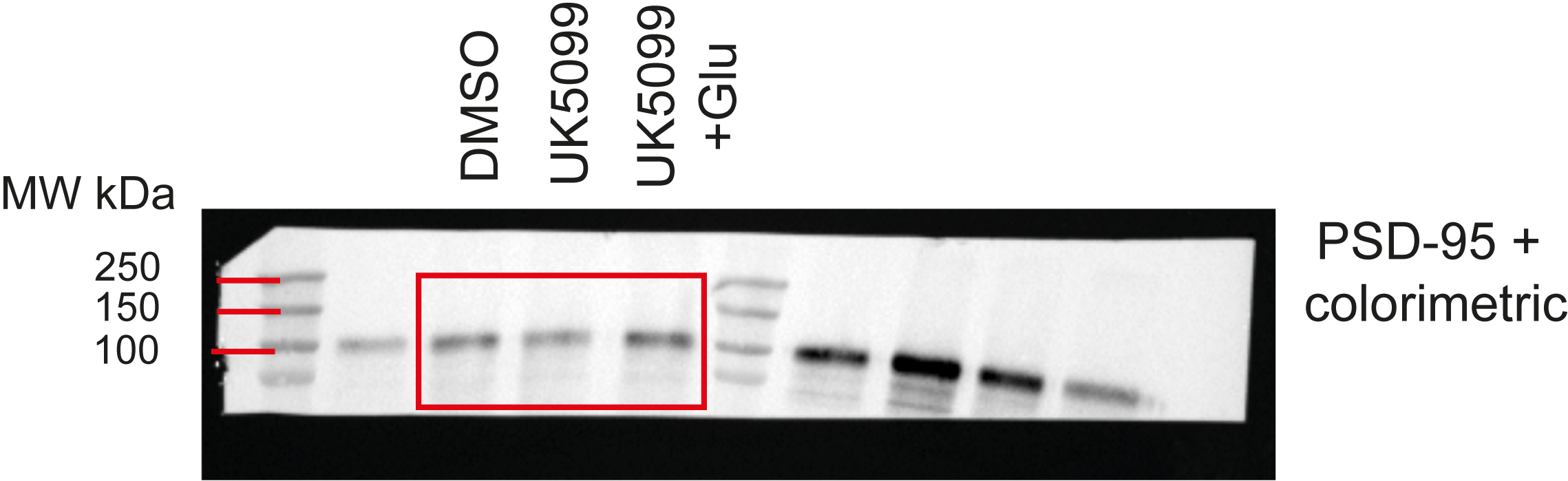

Supplement: Supplementary file 11 — Source Data Fig. 7 [file 44319_2023_48_MOESM11_ESM.zip › Figure 7/Figure 7F/Western PSD-95.tif]

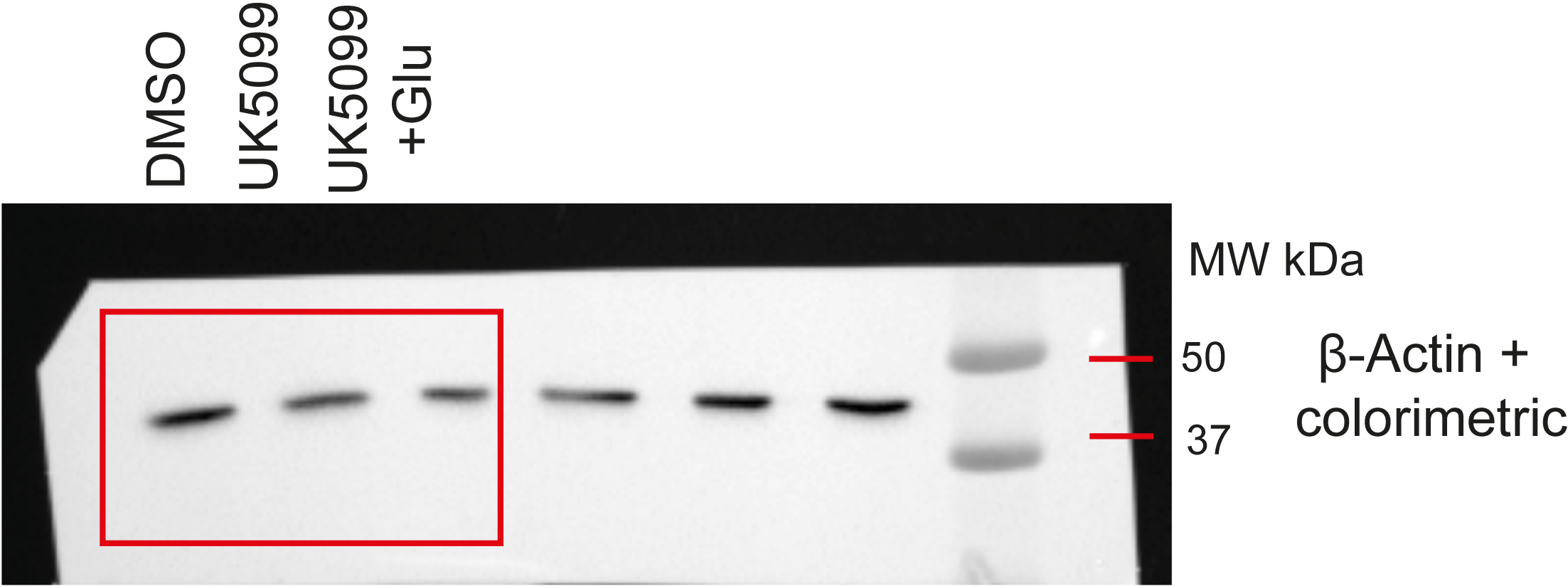

Supplement: Supplementary file 11 — Source Data Fig. 7 [file 44319_2023_48_MOESM11_ESM.zip › Figure 7/Figure 7A/Western Actin.tif]

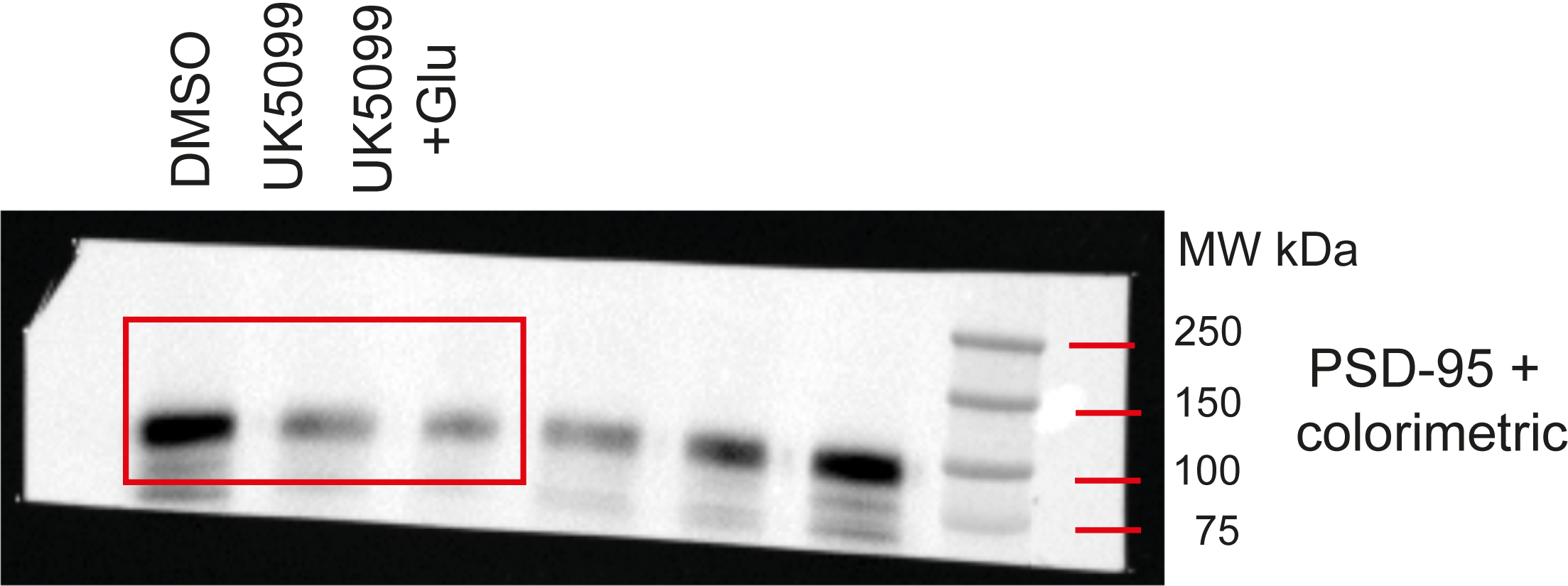

Supplement: Supplementary file 11 — Source Data Fig. 7 [file 44319_2023_48_MOESM11_ESM.zip › Figure 7/Figure 7A/Western PSD95.tif]

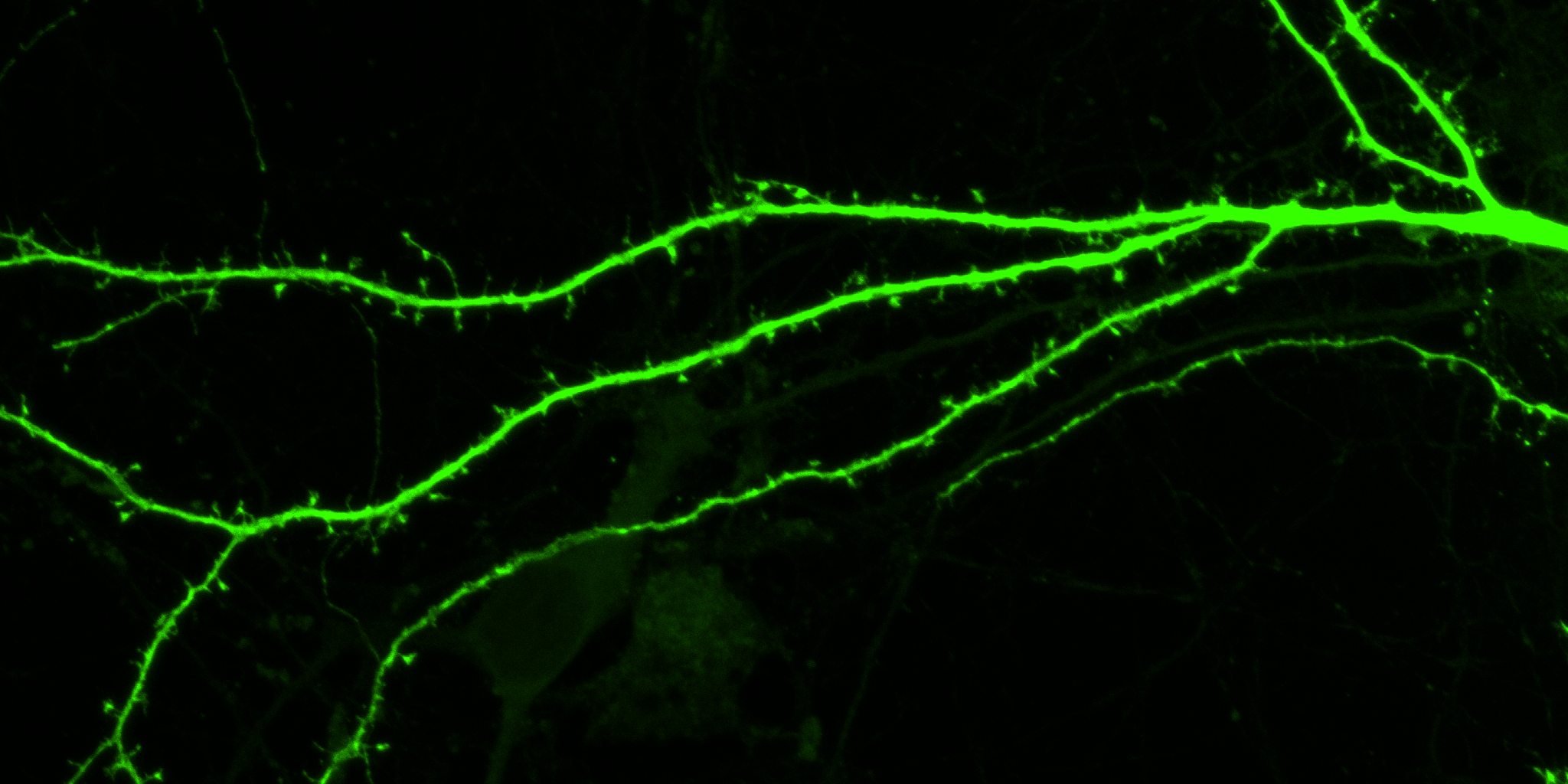

Supplement: Supplementary file 12 — Source Data Fig. 8 [file 44319_2023_48_MOESM12_ESM.zip › Figure 8/Figure 8D/UK+DHPG GFP.tif]

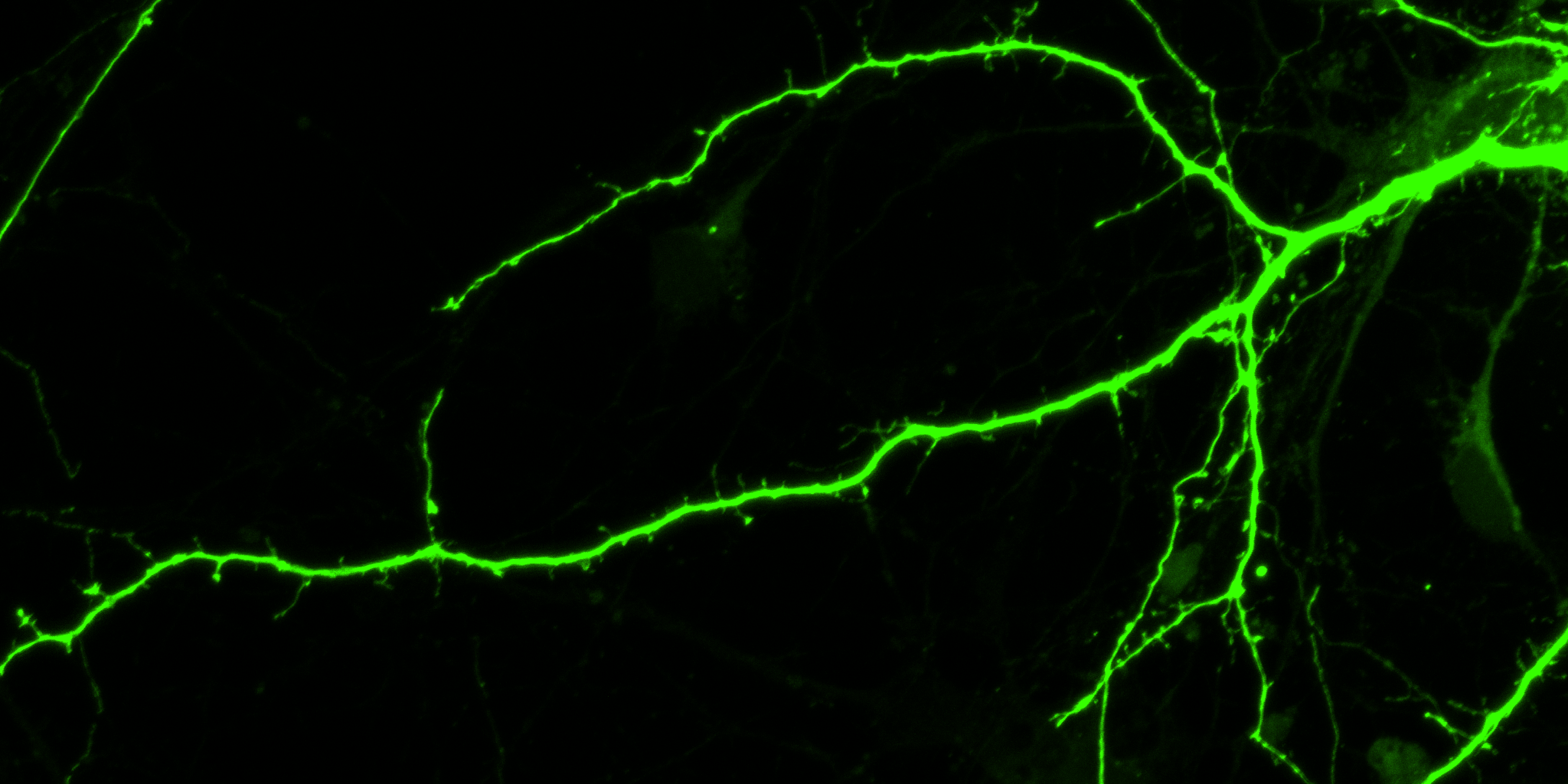

Supplement: Supplementary file 12 — Source Data Fig. 8 [file 44319_2023_48_MOESM12_ESM.zip › Figure 8/Figure 8D/UK GFP.tif]

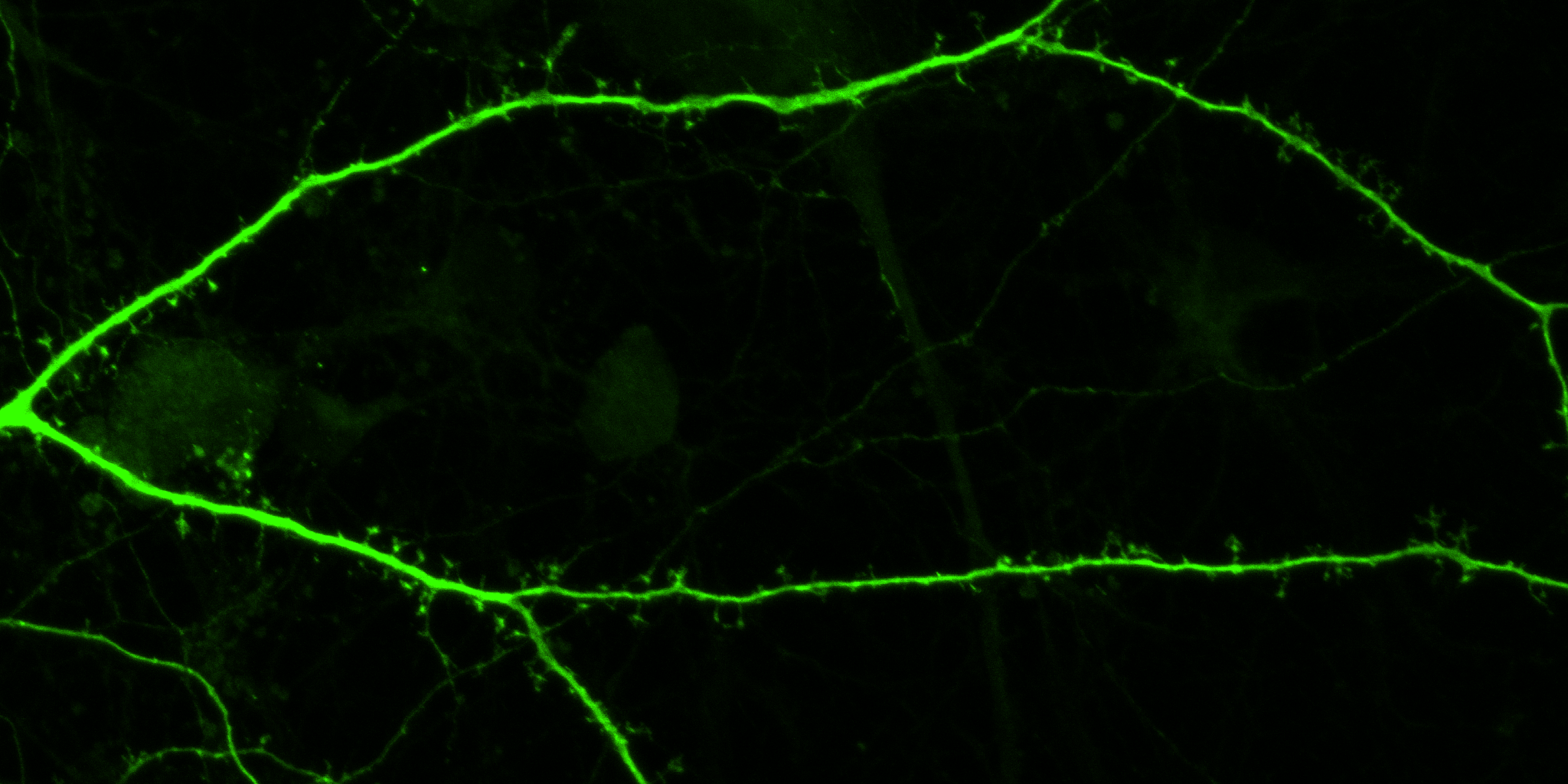

Supplement: Supplementary file 12 — Source Data Fig. 8 [file 44319_2023_48_MOESM12_ESM.zip › Figure 8/Figure 8D/DMSO GFP.tif]

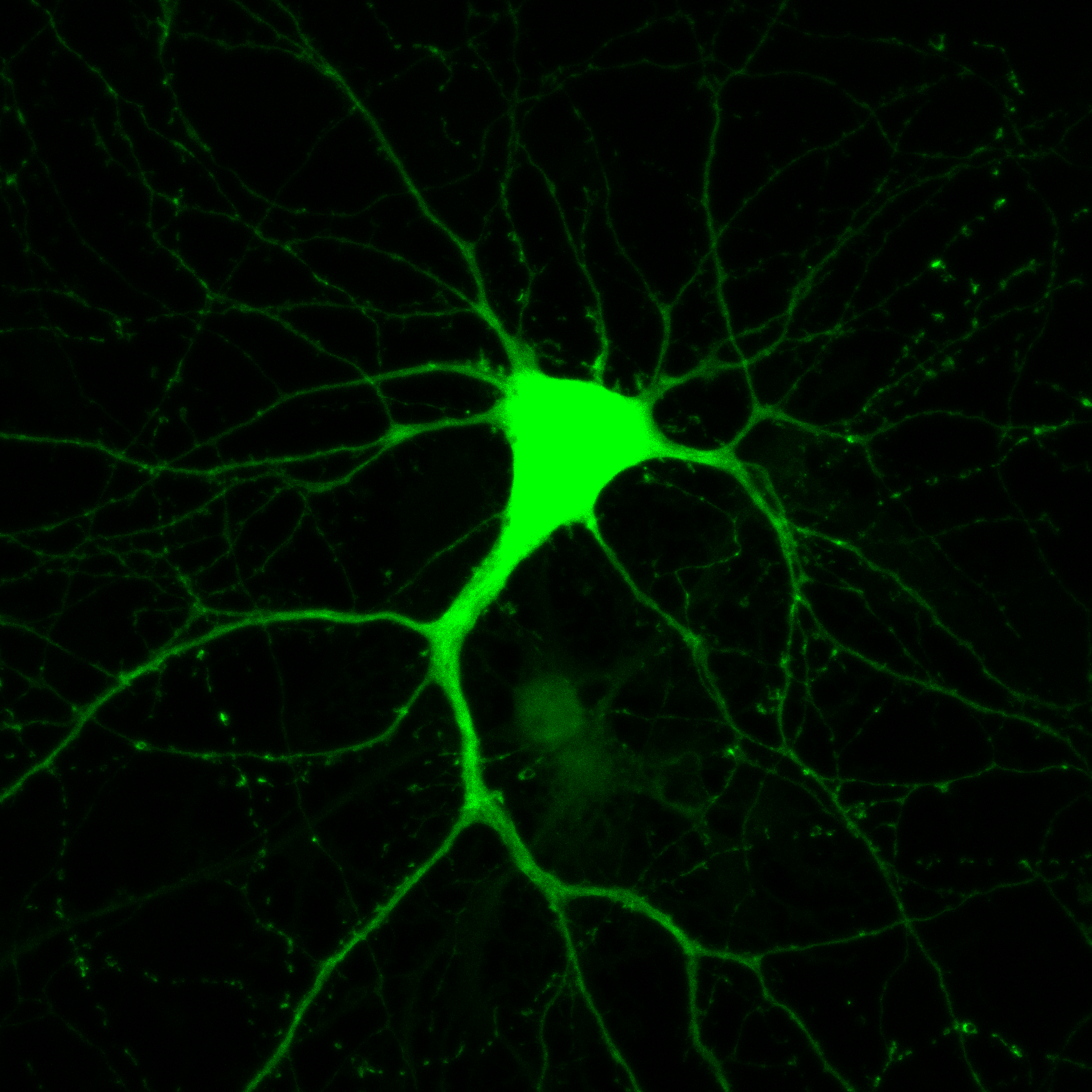

Supplement: Supplementary file 12 — Source Data Fig. 8 [file 44319_2023_48_MOESM12_ESM.zip › Figure 8/Figure 8C/UK5099 + DHPG GFP.tif]

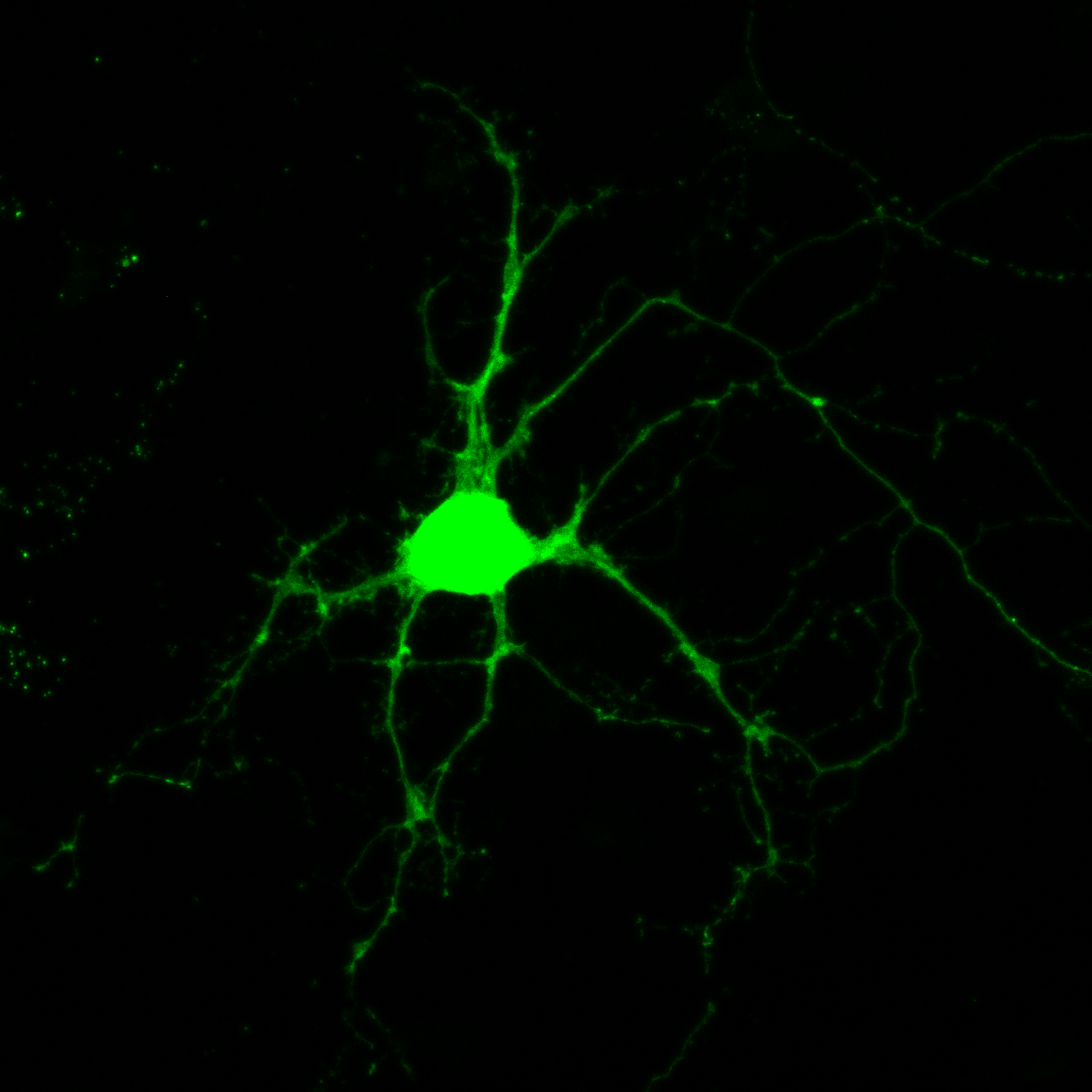

Supplement: Supplementary file 12 — Source Data Fig. 8 [file 44319_2023_48_MOESM12_ESM.zip › Figure 8/Figure 8C/UK5099 GFP.tif]

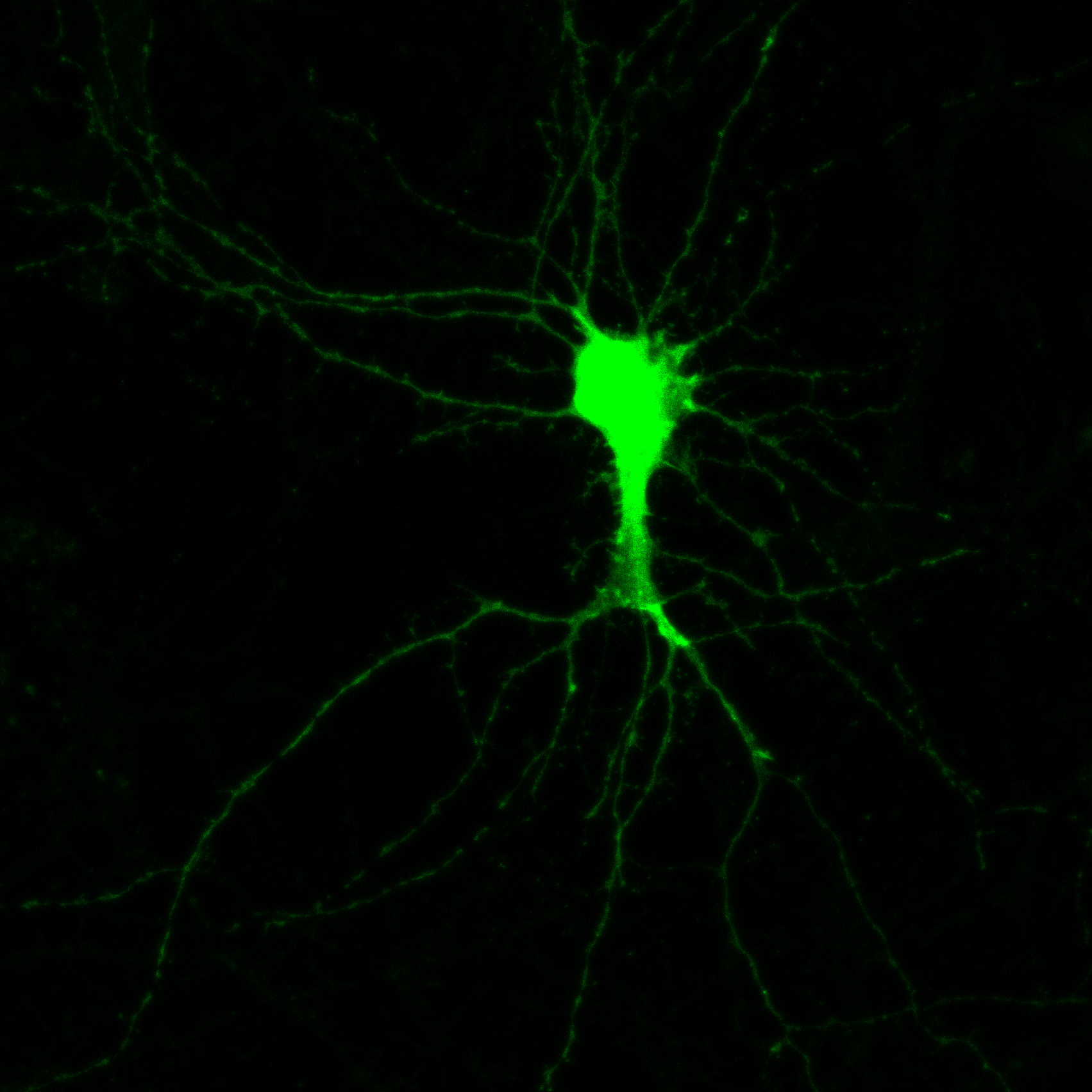

Supplement: Supplementary file 12 — Source Data Fig. 8 [file 44319_2023_48_MOESM12_ESM.zip › Figure 8/Figure 8C/DMSO GFP.tif]

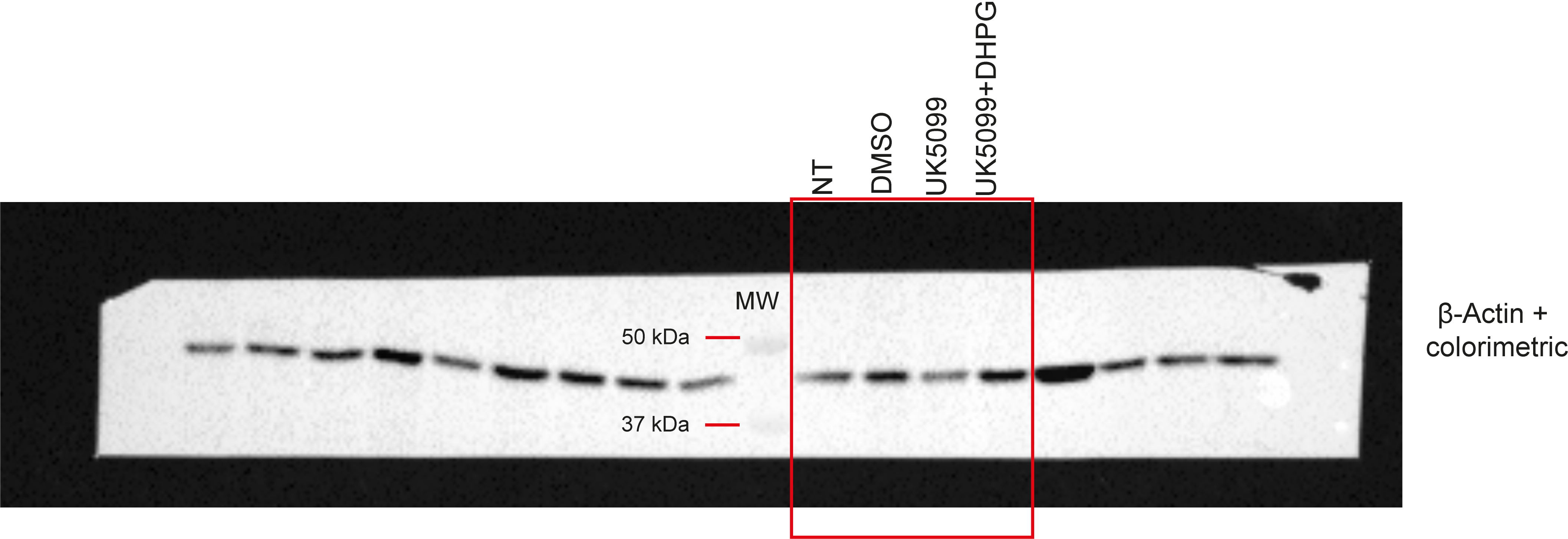

Supplement: Supplementary file 12 — Source Data Fig. 8 [file 44319_2023_48_MOESM12_ESM.zip › Figure 8/Figure 8B/Western Actin.tif]

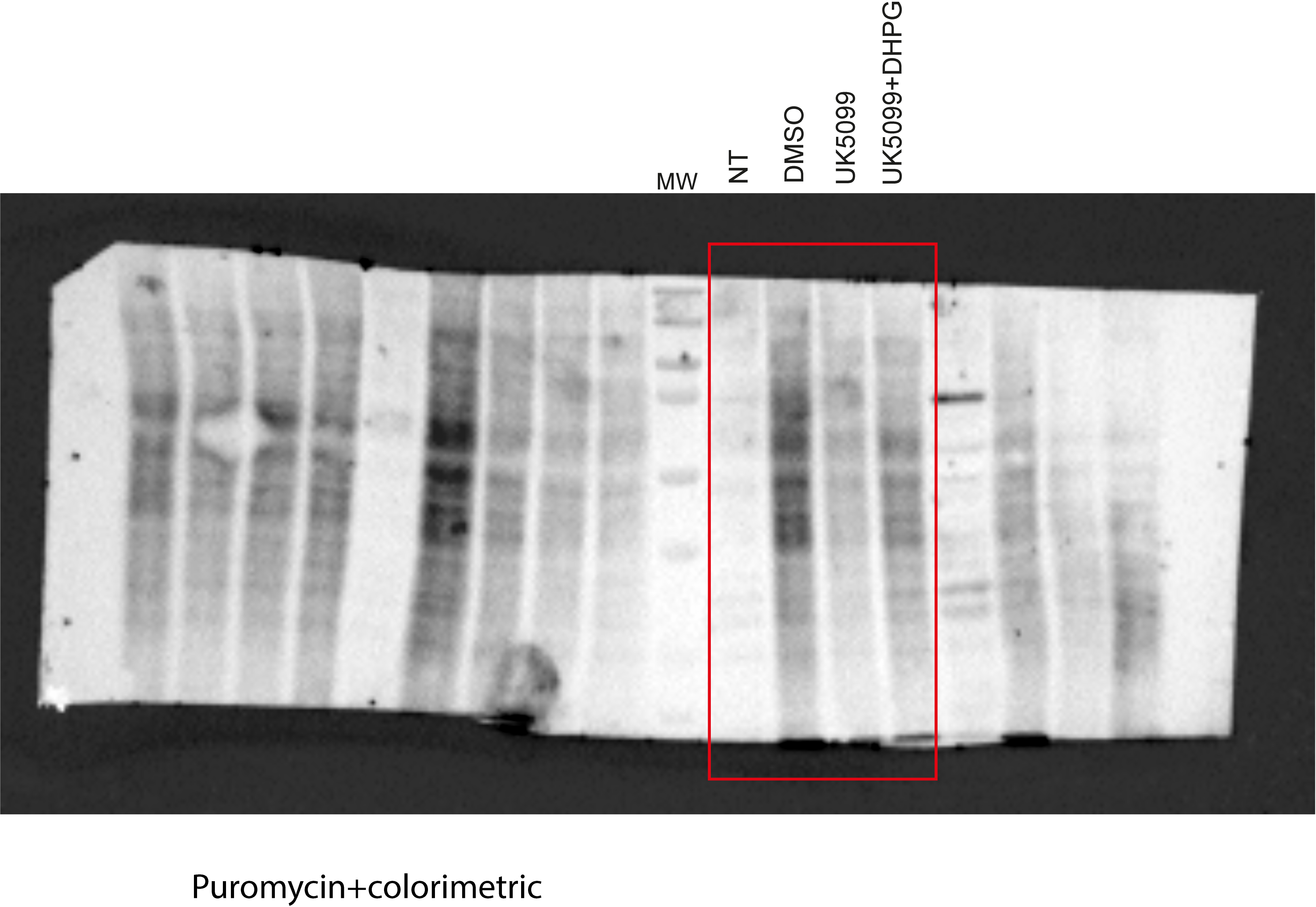

Supplement: Supplementary file 12 — Source Data Fig. 8 [file 44319_2023_48_MOESM12_ESM.zip › Figure 8/Figure 8B/Western DHPG.tif]

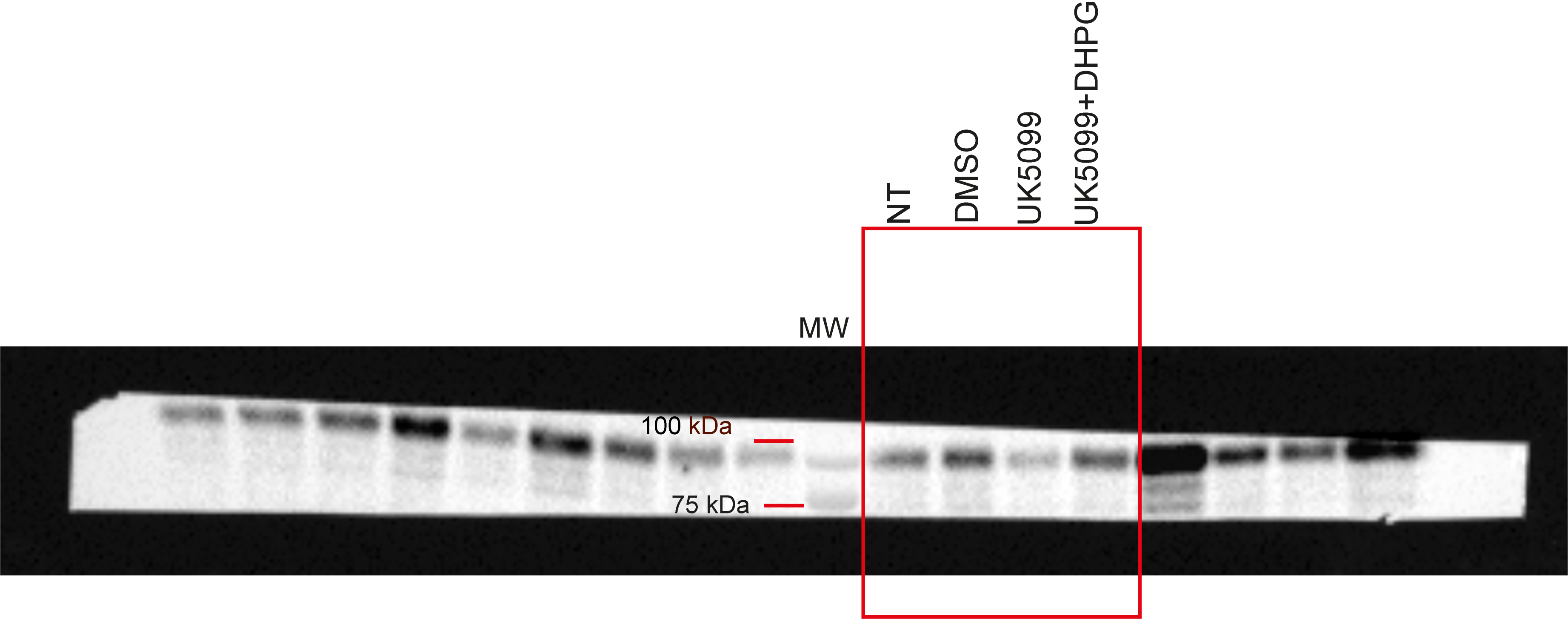

Supplement: Supplementary file 12 — Source Data Fig. 8 [file 44319_2023_48_MOESM12_ESM.zip › Figure 8/Figure 8B/Western PSD95.tif]
