# Supplementary material for: Glucose-derived glutamate drives neuronal terminal differentiation in vitro
Source: EMBO Rep. 2024 Jan 19;25(3):10. doi: 10.1038/s44319-023-00048-8 (PMC10933318; doi:10.1038/s44319-023-00048-8)
Supplement: Supplementary file 14 — Expanded View Figures [file 44319_2023_48_MOESM14_ESM.pdf]

Expanded View Figures

Figure EV1. Gene set enrichment analysis and effect of UK5099 administration to DIV10 hippocampal neurons.

(**A**) Gene set enrichment analysis showing that the respiratory chain was the only metabolic pathway among the most enriched gene clusters during neuronal differentiation from neuroblasts (NBs). Key pathways during differentiation are indicated in red. False discovery rate (FDR) < 0.02. (**B**) Timeline of the experimental procedure. Primary hippocampal neurons were isolated and plated (DIV0) and the transfection with a plasmid to express EGFP was performed on DIV7. UK5099 was added to the medium at DIV10 and dendritic arborization was analyzed at DIV15. (**C**) Representative confocal images (left panels) of DIV15 primary hippocampal neurons transfected with EGFP and treated at DIV10 with UK5099 (UK5099 DIV10-15) or DMSO as a control (scale bar 30 μm). The graphs on the right show the quantification of the dendritic arborization and total dendritic length ($n = 3$ biological replicates, >20 neurons for each condition; Sholl analysis: multiple t-test, df = 4, t = 4.02 *$p = 0.0158$, t = 2.94 *$p = 0.0423$, t = 4.05 *$p = 0.0154$, t = 4.059 *$p = 0.0153$, t = 2.808 *$p = 0.0483$, t = 3.361 *$p = 0.0282$, t = 3.741 *$p = 0.0201$, t = 4.326 *$p = 0.0123$, t = 3.891 *$p = 0.0176$, and t = 3.589 *$p = 0.0229$ at 50, 55, 60, 65, 70, 75, 80, 85, 90, and 100 μm from the soma, respectively. Dendritic length: paired t-test t = 4.838, df = 2, *$p = 0.0402$). (**D**) Comparison of neuronal arborization (left panel) and dendritic length (right panel) in neurons treated with UK5099 at DIV1 (UK5099 DIV1-15) and DIV10 (UK5099 DIV10-15), expressed as the percentage of the corresponding control conditions. UK5099 administration at DIV1 reduces dendritic length by 32% and the number of intersections at 100 μm by 43% compared to control conditions, while exposure to UK5099 at DIV10 leads to a 25% reduction in dendritic length and a 22% decrease in the number of intersections compared to control cells ($n = 3$ biological replicates; >20 neurons for each condition. Sholl analysis: multiple t-test df = 6, t = 2.602 *$p = 0.0405$ and t = 3.222 *$p = 0.0180$ at 95 and 100 μm from the soma, respectively. Dendritic length: paired t-test t = 7.937, df = 2, *$p = 0.015$). Data information: Data are reported as mean ± SE.

© The Author(s)

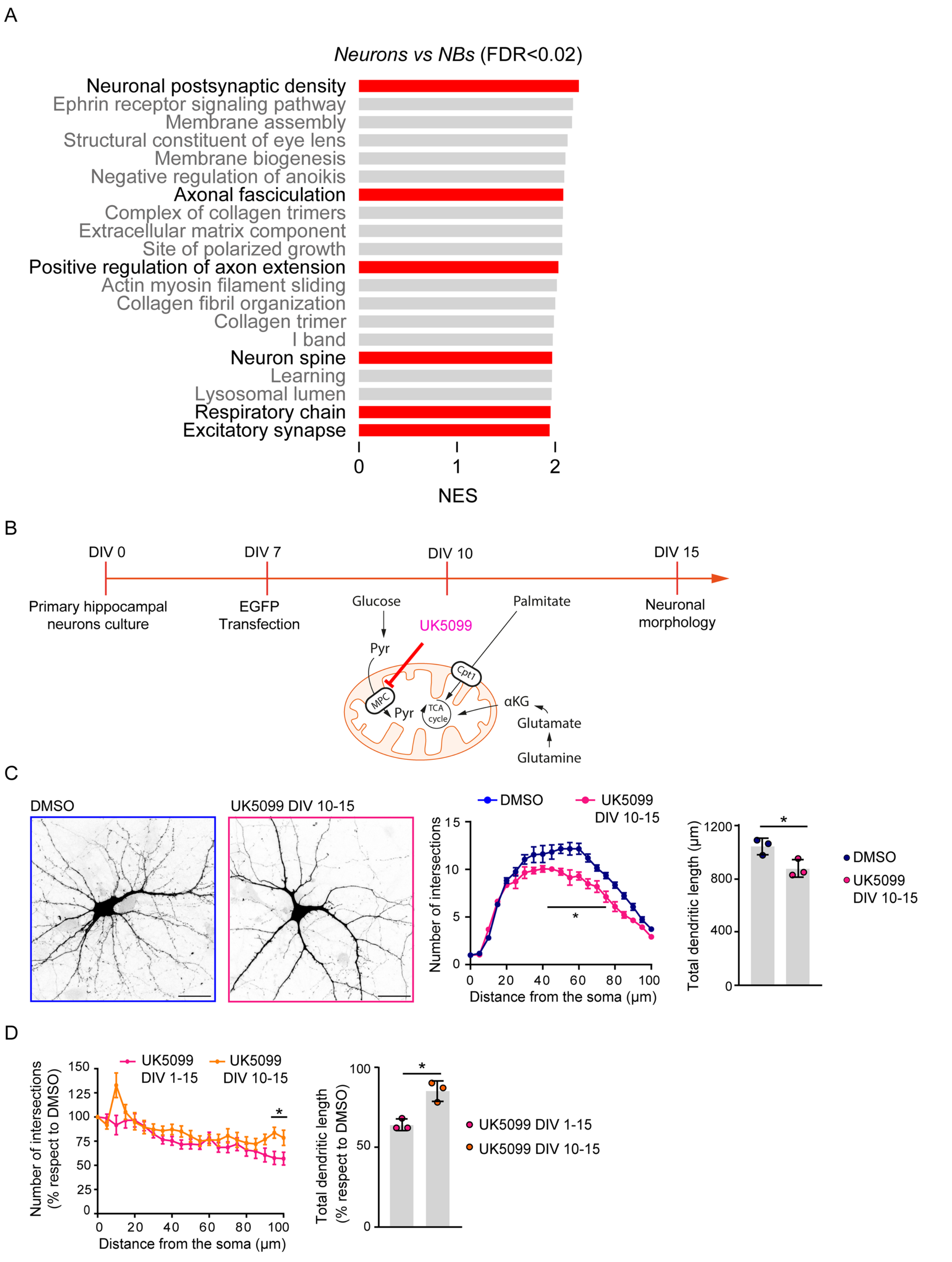
A
Neurons vs NBs (FDR<0.02)
Neuronal postsynaptic density
Ephrin receptor signaling pathway
Membrane assembly
Structural constituent of eye lens
Membrane biogenesis
Negative regulation of anoikis
Axonal fasciculation
Complex of collagen trimers
Extracellular matrix component
Site of polarized growth
Positive regulation of axon extension
Actin myosin filament sliding
Collagen fibril organization
Collagen trimer
I band
Neuron spine
Learning
Lysosomal lumen
Respiratory chain
Excitatory synapse
0 1 2
NES
B
DIV 0
DIV 7
DIV 10
DIV 15
Primary hippocampal neurons culture
EGFP Transfection
Glucose
UK5099
Palmitate
Neuronal morphology
Pyr
MPC
Pyr
TCA cycle
Cpt1
αKG
Glutamate
Glutamine
C
DMSO
UK5099 DIV 10-15
DMSO
UK5099 DIV 10-15
Number of intersections
Distance from the soma (µm)
Total dendritic length (µm)
DMSO
UK5099 DIV 10-15
D
UK5099 DIV 1-15
UK5099 DIV 10-15
Number of intersections (% respect to DMSO)
Distance from the soma (µm)
Total dendritic length (% respect to DMSO)
UK5099 DIV 1-15
UK5099 DIV 10-15

 © The Author(s)

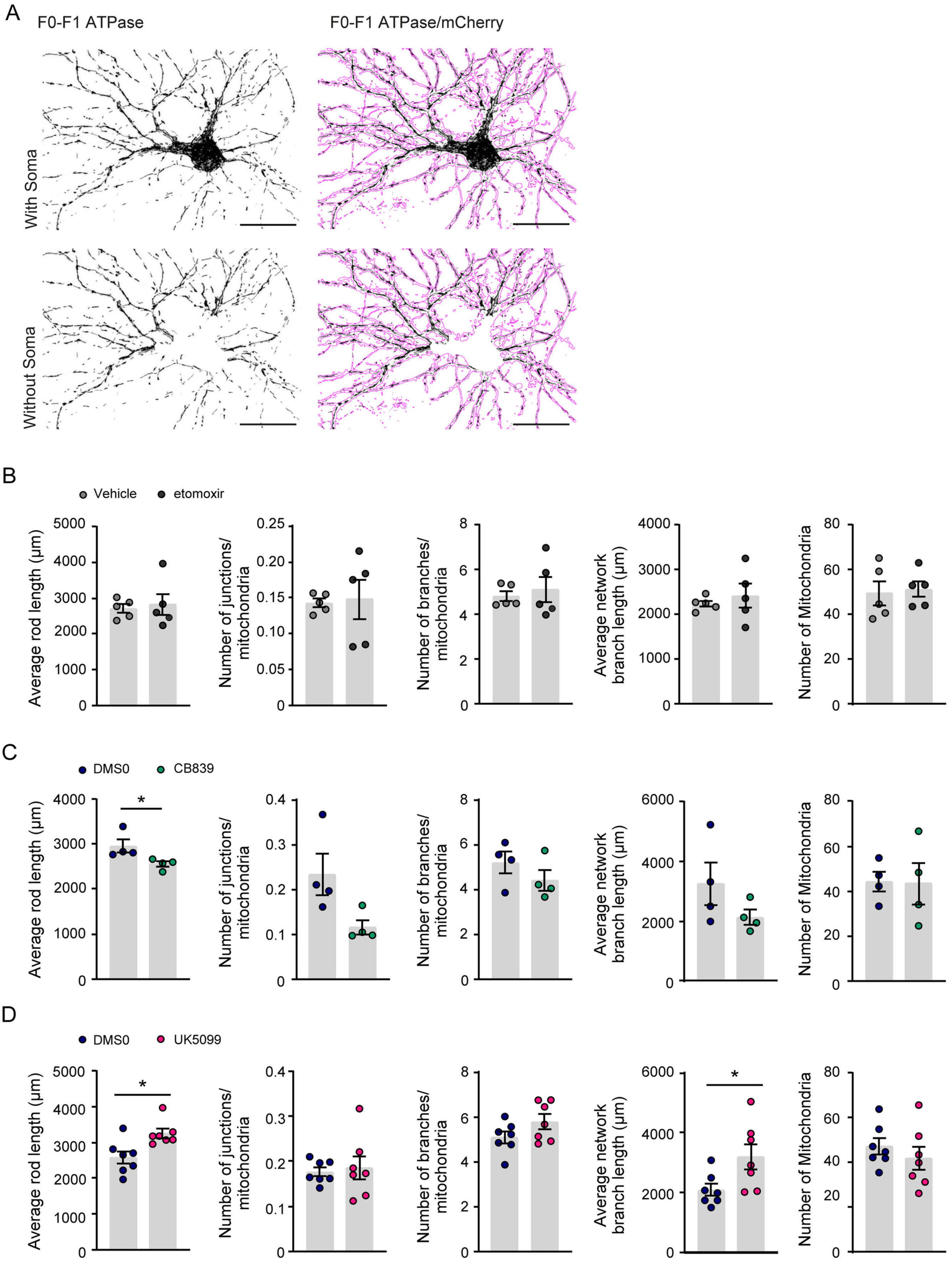
A
F0-F1 ATPase
F0-F1 ATPase/mCherry
With Soma
Without Soma
B
Vehicle
etomoxir
Average rod length (µm)
Number of junctions/ mitochondria
Number of branches/ mitochondria
Average network branch length (µm)
Number of Mitochondria
C
DMS0
CB839
D
DMS0
UK5099

Figure EV2. Effect of etomoxir, CB839, and UK5099 treatments on mitochondrial morphology.

(**A**) Representative confocal images of primary hippocampal neurons transfected with a plasmid to express GFP-tagged subunit 9 of the F0-F1 ATPase protein (to label mitochondria, in black) together with a plasmid encoding the mCherry protein (magenta outline). Analysis was performed along dendrites, excluding the contribution of the soma (scale bar 30 μm). (**B–D**) Analysis of mitochondrial morphology (average rod length, junction and branch number, and average branch length) in neurons treated with etomoxir (**B**), CB839 (**C**), or UK5099 (**D**) and transfected with the plasmids to express mCherry and GFP-tagged subunit 9 of the F0-F1 ATPase (Etomoxir vs. vehicle: 5 biological replicates, >30 neurons were analyzed for each condition. Paired t-test t = 0.3445 df = 4 $p = 0.7479$, t = 0.1928 df = 4 $p = 0.8565$, t = 0.7846 df = 4 $p = 0.4766$, t = 0.2840 df = 4 $p = 0.7905$ for mitochondrial average rod length, junction numbers, network branch length and mitochondria number, respectively; Wilcoxon matched signed rank test $p = 0.8125$ for branch number. CB839 vs. DMSO: 4 biological replicates, >20 neurons were analyzed for each condition. Paired t-test t = 3.389 df = 3 *$p = 0.0428$ for mitochondrial average rod length, t = 1.205, df = 3 $p = 0.3147$ for branch number, t = 1.509, df = 3 $p = 0.2285$ for average network branch length and t = 0.08989 df = 3 $p = 0.9340$ for mitochondria number; Wilcoxon matched signed rank test $p = 0.1250$, for junction number. UK5099 vs. DMSO: $n = 7$ biological replicates, >20 neurons were analyzed for each condition. Wilcoxon matched signed rank test *$p = 0.0156$ for mitochondrial average rod length, $p = 0.9375$ for junction number, Paired t-test t = 1.270 df = 6 $p = 0.2513$, t = 2.669 df = 6 *$p = 0.0371$, and t = 1.780 df = 6, $p = 0.1254$ for branch number, average network branch length, and mitochondria number, respectively). Data information: Data are reported as mean ± SE.

© The Author(s)

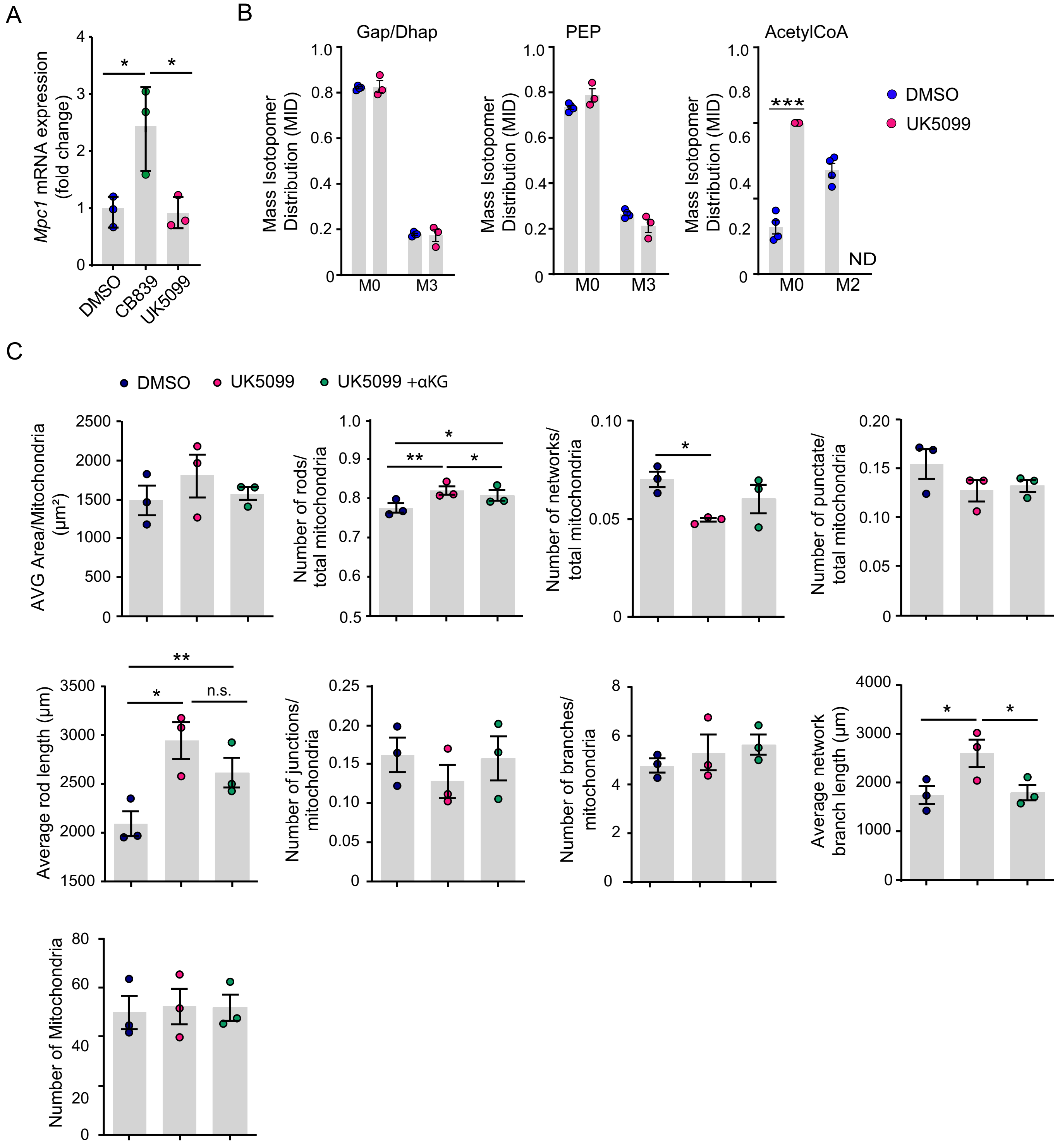
A
B
C
Mpc1 mRNA expression (fold change)
DMSO
CB839
UK5099
Gap/Dhap
PEP
AcetylCoA
Mass Isotopomer Distribution (MID)
M0
M3
M2
ND
DMSO
UK5099
UK5099 +αKG
AVG Area/Mitochondria (μm²)
Number of rods/ total mitochondria
Number of networks/ total mitochondria
Number of punctate/ total mitochondria
Average rod length (μm)
n.s.
Number of junctions/ mitochondria
Number of branches/ mitochondria
Average network branch length (μm)
Number of Mitochondria

Figure EV3. *Mpc1* expression is upregulated upon CB839 treatment; analysis of isotopomers in UK5099-treated cells and effect of α-ketoglutarate supplementation on mitochondrial morphology in neurons exposed to UK5099.

(**A**) RT-qPCR for *Mpc1* mRNA normalized to the housekeeping gene *36B4* in neurons treated with CB839 or UK5099 compared to DMSO. Statistical analysis was performed using one-way ANOVA with Tukey's multiple comparison test ($n = 3$ biological replicates, *$p < 0.05$ vs. DMSO or UK5099). Data are presented as mean ± SD. (**B**) Mass isotopomer distribution (MID) of glyceraldehyde 3-phosphate/dihydroxyacetone phosphate (Gap/Dhap), phosphoenolpyruvate (PEP), and acetyl-CoA labeled with [U-$^{13}C_6$] glucose. $n = 4$ biological replicates for DMSO and $n = 3$ biological replicates for UK5099. Statistical analysis was performed using Student's t-test. ***$p < 0.001$ vs. DMSO. Data are presented as mean ± SD. (**C**) Mitochondrial morphology analysis along dendrites of neurons treated with DMSO and UK5099 alone or in combination with α-ketoglutarate and transfected with plasmids to express mCherry and the GFP-tagged SV9 F0-F1 ATPase protein. Quantification of the average area of mitochondria, rods, and networks and the number of punctate mitochondria (upper panels, from left to right); mitochondrial average rod length, junction and branch numbers, and average branch length (lower graphs from left to right), number of mitochondria ($n = 3$ biological replicates, ≥20 neurons were analyzed for each condition, Friedman with Dunn's multiple comparison test for average area UK5099 vs. DMSO $p = 0.30748$, UK5099 + αKG vs. DMSO and UK5099 vs. UK5099 + αKG > 0.9999 and for punctate mitochondria UK5099 vs. DMSO $p = 0.123$, UK5099 + αKG vs. DMSO $p = 0.3074$, UK5099 vs. UK5099 + αKG $p > 0.9999$; RM one-way ANOVA with Tukey's multiple comparison test: number of rods UK5099 vs. DMSO **$p < 0.01$; UK5099 + αKG vs. DMSO, UK5099 + αKG vs. UK5099 *$p < 0.05$; number of networks, UK5099 vs. DMSO *$p = 0.0419$, UK5099 + αKG vs. DMSO $p = 0.6943$ and UK5099 + αKG vs. UK5099 $p = 0.4916$; average rod length *$p = 0.047$ UK5099 vs. DMSO, **$p = 0.0056$ UK5099 + αKG vs. DMSO, $p = 0.2215$ UK5099 + αKG vs. UK5099; number of junctions UK5099 vs. DMSO, UK5099 + αKG vs. DMSO, and UK5099 + αKG vs. UK5099: not significant; number of branches UK5099 vs. DMSO, UK5099 + αKG vs. DMSO and UK5099 + αKG vs. UK5099: not significant; average network branch length: UK5099 vs. DMSO *$p = 0.0187$, UK5099 + αKG vs. DMSO $p = 0.4021$, UK5099 + αKG vs. UK5099 $p = 0.0418$; number of mitochondria UK5099 vs. DMSO, UK5099 + αKG vs. DMSO, and UK5099 + αKG vs. UK5099: not significant). Data information: Data are reported as mean ± SE.

 © The Author(s)

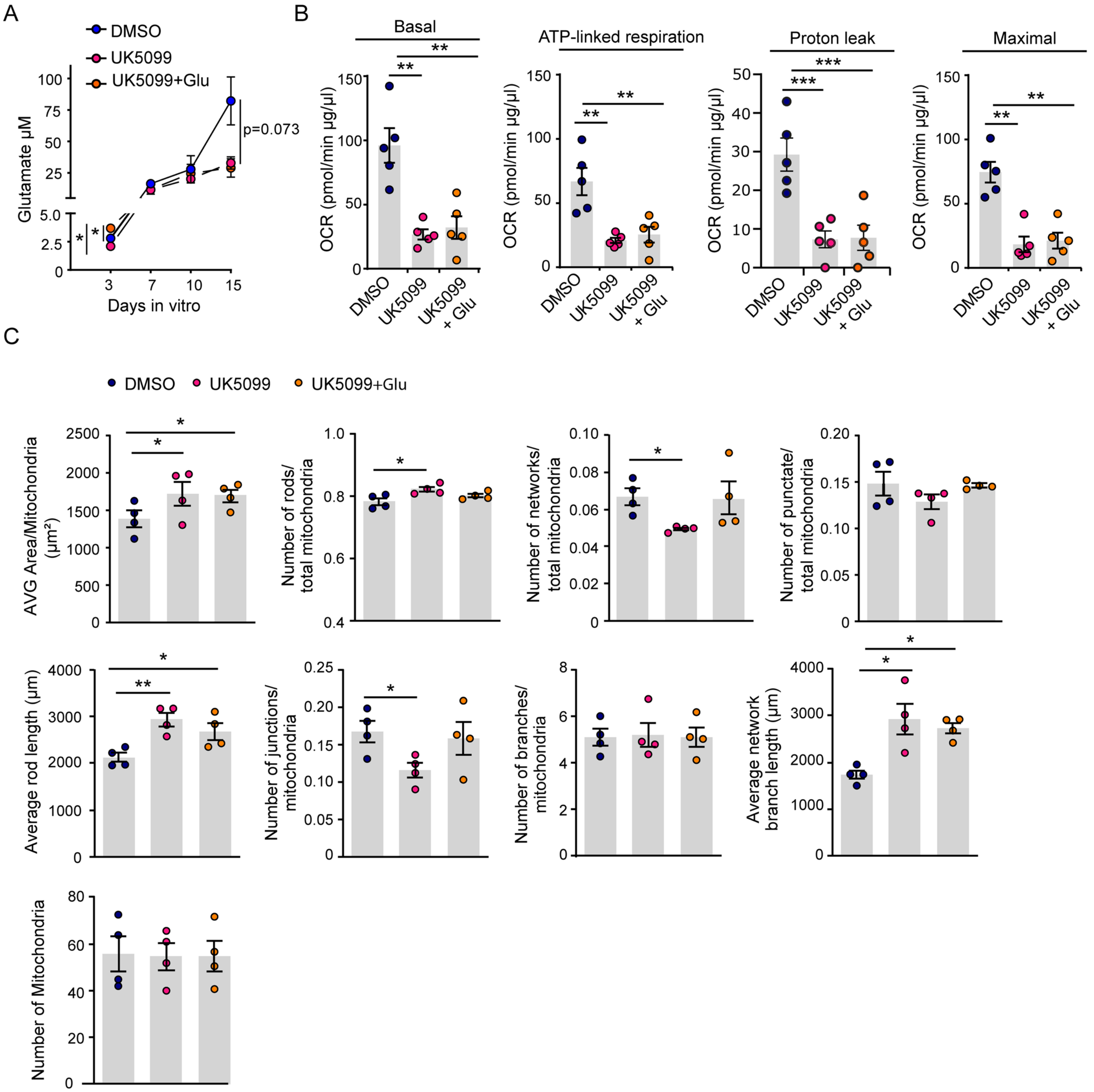
A
DMSO
UK5099
UK5099+Glu
Glutamate μM
Days in vitro
p=0.073
B
Basal
ATP-linked respiration
Proton leak
Maximal
OCR (pmol/min μg/μl)
DMSO
UK5099
UK5099 + Glu
C
DMSO UK5099 UK5099+Glu
AVG Area/Mitochondria (μm²)
Number of rods/ total mitochondria
Number of networks/ total mitochondria
Number of punctate/ total mitochondria
Average rod length (μm)
Number of junctions/ mitochondria
Number of branches/ mitochondria
Average network branch length (μm)
Number of Mitochondria

Figure EV4. Effect of glutamate supplementation on mitochondrial activity and morphology in neurons exposed to UK5099.

(**A**) Glutamate quantification in media from cells treated with DMSO or UK5099 alone or in combination with glutamate at the indicated time points. Statistical analysis was performed using two-way ANOVA with Tukey's multiple comparison test ($n = 3$ biological replicates, $*p < 0.05$ vs. DMSO or vs. UK5099). Data information: Data are presented as mean ± SD. (**B**) Seahorse XFe24 Cell Mito Stress Test for basal, ATP-linked, proton leak, and maximal uncoupled respiration. Oxygen consumption rate analyses were performed on DIV15 neurons treated with DMSO, UK5099, and UK5099 + glutamate (Glu). ($n = 5$ biological replicates, one-way ANOVA with Tukey's multiple comparison test; Basal: $F(2,8) = 20.47$ df = 8, UK5099 vs. DMSO $**p = 0.0011$, UK5099 + Glu vs. DMSO $**p = 0.0018$, UK5099 + Glu vs. UK5099 $p = 0.8991$; ATP-linked respiration: $F(2,8) = 16.04$ df = 8, UK5099 vs. DMSO $**p = 0.0023$, UK5099 + Glu vs. DMSO $**p = 0.0042$, UK5099 + Glu vs. UK5099 p = 0.8734; proton leak: $F(2,8) = 26.14$ df = 8, UK5099 vs. DMSO $***p = 0.0006$, UK5099 + Glu vs. DMSO $***p = 0.0007$, UK5099 + Glu vs. UK5099 $p = 0.9917$; maximal: $F(2,8) = 20.29$ df = 8, UK5099 vs. DMSO $**p = 0.0012$, UK5099 + Glu vs. DMSO $**p = 0.0017$, UK5099 + Glu vs. UK5099 $p = 0.9583$). Data information: Data are presented as mean ± SE. (**C**) Neurons were treated with DMSO, UK5099, or the combination of UK50999 with Glutamate and transfected with the plasmids to express mCherry and GFP-tagged SV9 F0-F1 ATPase. Quantification of the average mitochondrial area; average number of rod, network, and punctate mitochondria; average rod length; junction, and branch numbers; average network branch length; and mitochondria number was performed (4 biological replicates; >25 neurons were analyzed for each condition. RM ANOVA with Tukey's multiple comparison test for average area, UK5099 vs. DMSO $*p = 0.0350$, UK5099 + Glu vs. DMSO $*p = 0.0172$, UK5099 + Glu vs. UK5099 $p = 0.9502$; number of rods UK5099 vs. DMSO $*p = 0.0251$, UK5099 + Glu vs. DMSO $p = 0.3318$ and UK5099 + Glu vs. UK5099 $p = 0.2942$; number of networks UK5099 vs. DMSO $*p = 0.0495$, UK5099 + Glu vs. DMSO $p = 0.9972$ and UK5099 + Glu vs. UK5099 $p = 0.2798$; average rod length UK5099 vs. DMSO $**p = 0.0027$, UK5099 + Glu vs. DMSO $*p = 0.0308$, UK5099 + Glu vs. UK5099 $p = 0.1285$; number of junctions UK5099 vs. DMSO $*p = 0.0391$, UK5099 + Glu vs. DMSO $p = 0.8747$ and UK5099 + Glu vs. UK5099 $p = 0.1391$; number of branches UK5099 vs. DMSO, UK5099 + Glu vs. DMSO and UK5099 + Glu vs. UK5099 not significant; average network branch length UK5099 vs. DMSO $*p = 0.0498$, UK5099 + Glu vs. DMSO $*p = 0.0152$, UK5099 + Glu vs. UK5099 $p = 0.8614$; mitochondria number UK5099 vs. DMSO, UK5099 + Glu vs. DMSO and UK5099 + Glu vs. UK5099 not significant; Friedmann test with Dunn's multiple comparison test for number of punctate mitochondria UK5099 vs. DMSO $p = 0.4719$, UK5099 + Glu vs. DMSO $p > 0.9999$, UK5099 + Glu vs. UK5099 $p = 0.2313$). Data information: Data are reported as mean ± SE.

 © The Author(s)

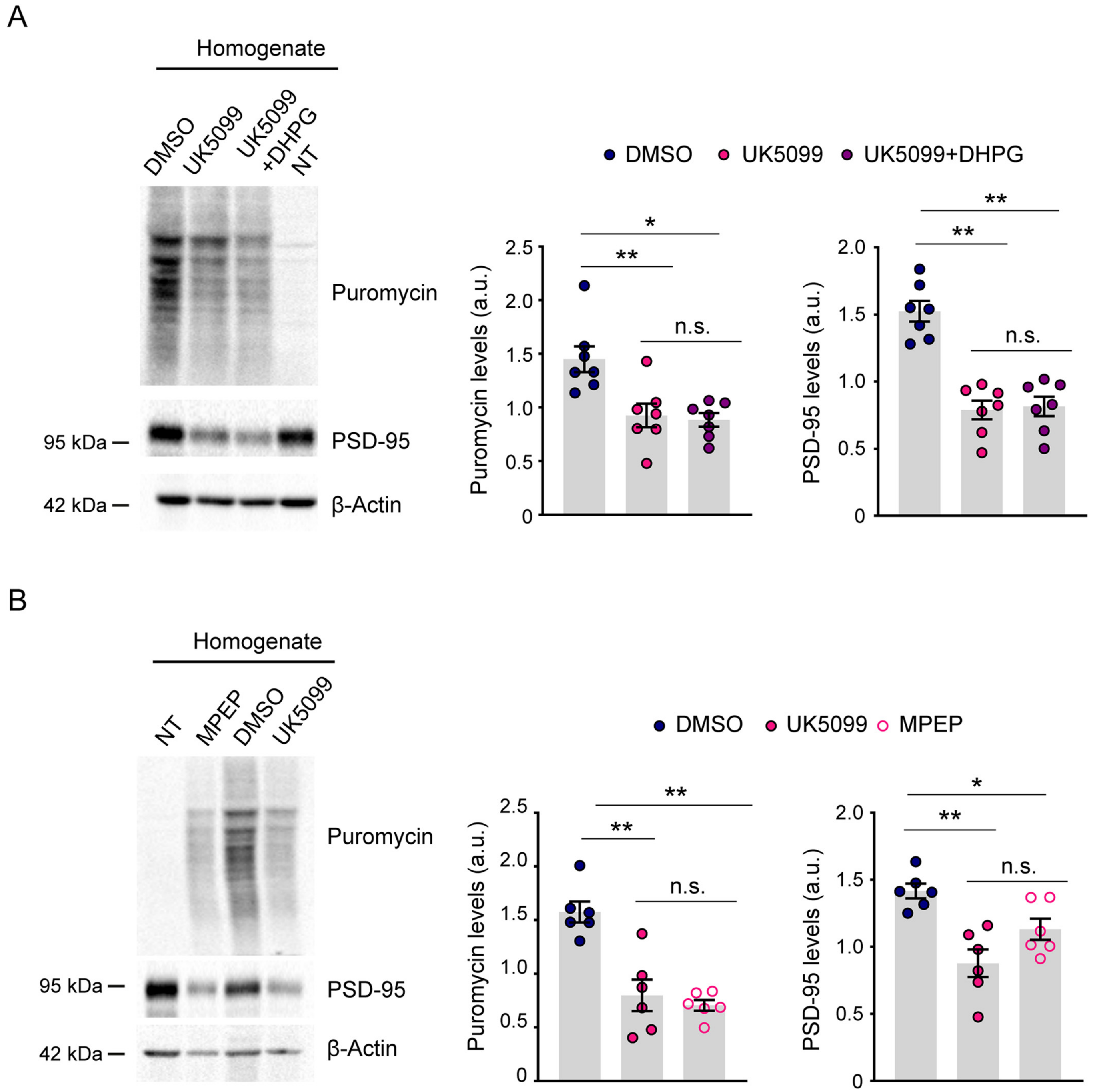

Figure EV5. Effect of DHPG and MPEP on global protein synthesis and PSD-95 levels in total extract of UK5099-treated neurons.

(**A**,**B**) Representative western blot and quantification of puromycin incorporation and PSD-95 expression in total extract of DIV15 rat primary neurons treated at DIV1 with UK5099 in presece/absence of DHPG (**A**) and treated with UK5099 and MPEP (**B**). β-Actin was used as loading control and normalyzer. Data are shown as mean ± SE (Ordinary one-way ANOVA, Tukey's multiple comparison test. A: $n = 7$ biological replicates. Puromycin: UK5099 vs DMSO $^{**}p = 0.0060$, UK5099 + DHPG vs DMSO $^{*}p = 0.0145$, UK5099 + DHPG vs UK5099 $p = 0.9451$; PSD-95: UK5099 vs DMSO $^{**}p = 0.0012$, UK5099 + DHPG vs DMSO $^{**}p = 0.0037$, UK5099 + DHPG vs UK5099 $p = 0.9696$; B: $n = 6$ biological replicates. Puromycin: UK5099 vs DMSO $^{**}p = 0.0043$, MPEP vs DMSO $^{**}p = 0.0017$, UK5099 vs MPEP $p = 0.8467$; PSD-95: UK5099 vs. DMSO $^{**}p = 0.0045$, MPEP vs DMSO $^{*}p = 0.0120$, UK5099 vs MPEP $p = 0.2183$).

© The Author(s)
